# Alkyl tails of cesium and formamidinium precursors control the growth of lead bromide perovskite nanocrystals

**DOI:** 10.1039/d6sc05095f

**Published:** 2026-09-15

**Authors:** Yuliia Berezovska, Valerie Sekula, Nikita Romanyuk, Benjamin Aymoz, Sebastian Sabisch, Maksym V. Kovalenko, Dmitry N. Dirin

**Affiliations:** a Department of Chemistry and Applied Biosciences, ETH Zürich CH-8093 Zürich Switzerland mvkovalenko@eth.ch ddirin@ethz.ch; b Empa-Swiss Federal Laboratories for Materials Science and Technology CH-8600 Dübendorf Switzerland

## Abstract

Access to strongly quantum-confined lead bromide perovskite quantum dots (QDs) remains limited by the rapid formation kinetics of the perovskite phase. A major advance was achieved by introducing tri-*n*-octylphosphine oxide (TOPO) to coordinate PbBr_2_ and slow QD growth, although obtaining sub-5 nm CsPbBr_3_ QDs still required extreme dilution and yielded only tens of micrograms of isolable QDs per milliliter of reaction solution. Here, we introduce molecular design of the A-site cation precursor as a practical handle for controlling QD growth. Using cesium phosphinates, phosphonates, and phosphates with systematically varied aliphatic substituents, we show that the headgroup has only a minor influence on QD growth, whereas the precursor tail length governs the final QD size in a nonmonotonic manner, with a threshold around C_8_ attributed to changes in precursor organization. Steric tuning of the cesium precursor enabled size control of CsPbBr_3_ QDs from 18 to 3 nm with up to 40-fold increase in yield relative to the conventional route. In practical terms, the difference is 0.8 mg mL^−1^*vs.* 18 µg mL^−1^ of isolable 3 nm QDs, with improved size dispersion (26 nm absorption linewidth). Even smaller CsPbBr_3_ species, estimated at 3–4 PbBr_6_ octahedra in width, are resolved by *in situ* absorption spectroscopy, opening the way to interrogate their nucleation and growth. The same strategy is extended to the more challenging FAPbBr_3_ system, enabling monodisperse QDs as small as 3.8 nm and allowing construction of a practical sizing curve relating FAPbBr_3_ QD size to the first excitonic absorption peak over the 3.8–12 nm size range.

## Abstract 2

Molecular design of A-site cation precursors provides a new practical handle for controlling lead bromide perovskite quantum dot growth, unlocking small quantum dots with practical yields and previously inaccessible optical quality.

## Graphical abstract

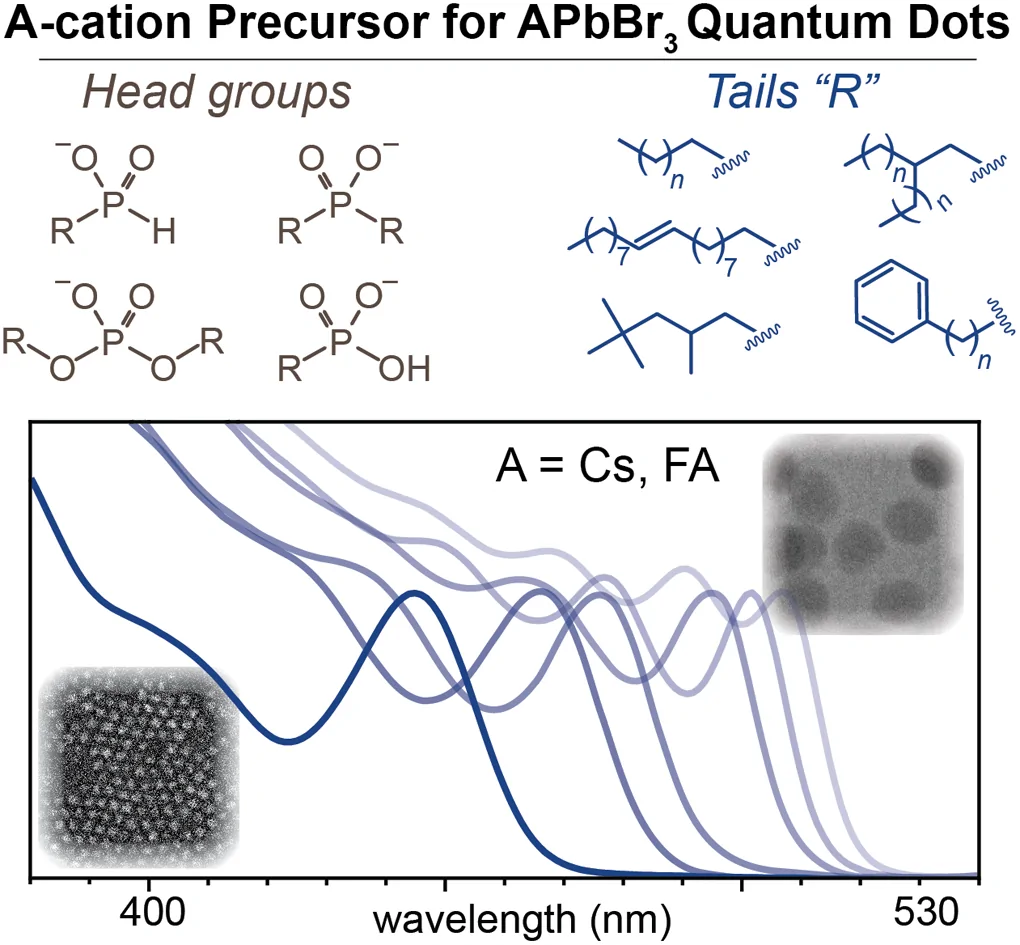

## Introduction

Controlling the size of colloidal quantum dots (QDs) has always been central to the design of conventional chalcogenide and pnictide QD syntheses.^1–3^ Achieving such control generally requires that the reactivity of at least one precursor, typically the least reactive one, be finely adjusted to regulate the monomer formation rate and the number of nuclei formed,^4–11^ as well as their subsequent growth rate.^12,13^ In established syntheses of metal chalcogenide and pnictide QDs, as well as oxide nanocrystals, reaction temperature is typically the primary operational parameter, effectively and conveniently modulating precursor conversion kinetics.^14–17^ This approach requires judicious selection of precursors that allow for broad and controllable reactivity tuning within a reasonable temperature window.^4,14^ For lead halide perovskite (LHP) QDs with the general formula APbX_3_ (A = Cs,^18^ methylammonium MA,^19^ formamidinium FA,^20–22^ aziridinium Az;^23^ X = Cl, Br, I), this temperature-based strategy has proven considerably less effective, especially for sub-5 nm QDs.^24^ Owing to the near-zero formation energy of LHPs,^25^ their precursors readily form the perovskite phase even at ambient temperatures,^26^ leading to rapid nucleation and growth and, consequently, limited control over QD size. A major advance in fine size control was achieved by introducing tri-*n*-octylphosphine oxide (TOPO) into the synthetic solution.^27^ TOPO was proposed to coordinate key intermediate species during the synthesis, ranging from the PbBr_2_ precursor to the proposed monomers and ultimately to QD nuclei, thereby modulating the chemical equilibria among these species. This approach enabled the synthesis of monodisperse CsPbBr_3_ QDs with tunable sizes from 3 to 13 nm, as well as MAPbBr_3_ and FAPbBr_3_ QDs with sizes between 6 and 11 nm, although accessing the smallest QDs required extreme dilution, yielding only tens of µg of isolable QDs per mL of crude solution.^27^

The role of the cesium precursors in the formation and growth of CsPbBr_3_ QDs has received far less attention. Both the A-site cation itself and its surfactant can influence the equilibria in solution^28^ as well as the surface chemistry of the resulting QDs.^29,30^ Hybrid organic–inorganic compositions such as MAPbBr_3_ and FAPbBr_3_ typically form within seconds to a minute – substantially faster than CsPbBr_3_ – even at lower precursor concentrations.^27^ The range of reported A-site cation counterions remains narrow and, beyond the diisooctylphosphinate used in the original report^27^ and its analog bis(2,4,4-trimethylpentyl)phosphinate,^31,32^ is largely limited to a few carboxylates, which have primarily been employed to yield either very large QDs^33^ or more reactive hybrid perovskite compositions.^23,27^ Although the number of such studies remains small, they nevertheless indicate that A-site cation precursor chemistry influences QD growth. We therefore view molecular design of A-site cation precursors as an underexplored yet powerful parameter for controlling the formation of LHP QDs.

Herein, we present cesium salts of phosphinic, phosphoric, and phosphonic acids bearing various mono- or di-substituted aliphatic or aromatic tails. The headgroup structure has only a minor influence on QD formation at practical synthetic concentrations, whereas even subtle changes in tail length alter nucleation and growth of QDs, although in a nonmonotonic manner. The reaction can be controlled *via* the precursor tail length and, for long tails, branching, enabling access to CsPbBr_3_ QDs spanning from strongly quantum-confined sizes (3–4 PbBr_6_ octahedra wide, observable with *in situ* absorption spectroscopy) to weakly confined particles as large as 18 nm. The same strategy was further extended to the more reactive FAPbBr_3_ system, establishing a sizing curve relating FAPbBr_3_ QD size to the position of the excitonic absorption peak and enabling access to monodisperse FAPbBr_3_ QDs as small as 3.8 nm without excessive dilution.

## Results and discussion

We adopt the synthetic approach reported in ref. 27 (Scheme 1a), in which the reaction is conducted at room temperature and slowed by employing tri-*n*-octylphosphine oxide (TOPO). TOPO coordinates to and dissolves the PbBr_2_ precursor, but can also participate in coordination to intermediate species and even the resulting QDs. In this system, the reaction is controlled either by the TOPO : Pb ratio or by lowering the Pb precursor concentration while keeping the Pb : Cs ratio unchanged and maintaining the same Cs precursor, namely cesium bis(6-methylheptyl)phosphinate (Cs-DOPA).^27^ Here, rather than tuning the reactivity of the Pb precursor, we instead systematically vary the chemistry of the Cs precursor under a fixed PbBr_2_ to TOPO ratio. As precursor concentration plays a crucial role in QD growth, all Cs precursors were evaluated over a broad concentration range of 0.3–3.4 mM, while maintaining a constant Pb : Cs molar ratio of 4 : 1, as in ref. 27. For each Cs precursor and concentration, the reaction was considered completed when no further QD growth was observed (see Section 3.3 of the SI for details, and Tables S1 and S2 for the synthesis conditions of each batch studied in this work).

**Scheme 1:**
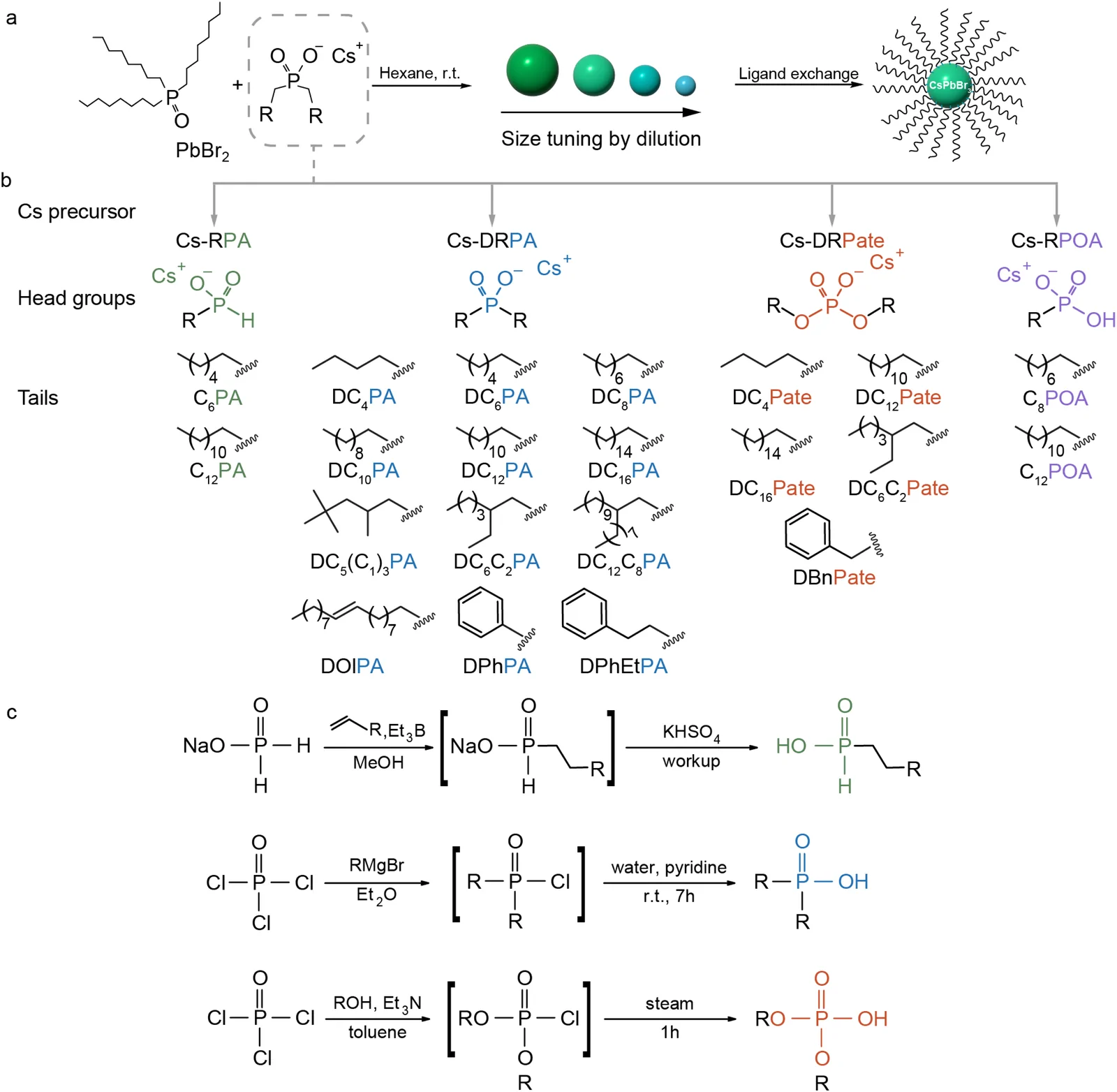
(a) Synthetic scheme for CsPbBr_3_ QDs. (b) Employed Cs monoalkylphosphinates, dialkylphosphinates, dialkylphosphates and monoalkylphosphonates. (c) Synthetic scheme for corresponding monoalkylphosphinic, dialkylphosphinic, and dialkylphosphoric acids. C_6_PA and C_12_PA were courtesy of the group of Prof. Jonathan De Roo, ref. 36.

We envisioned several ways by which the Cs precursor could influence QD synthesis:

1. The headgroup of the surfactant determines its Lewis basicity and chelating capability, thereby affecting its binding strength and potentially the kinetics of Cs release. These properties may also influence interactions with Pb species or with developing QD surfaces.^34,35^

2. The number, length, and branching of the alkyl substituents modulate precursor solubility and QD surface accessibility and, if reverse micelles form, may also affect Cs release kinetics.

3. Introducing aryl and oleyl functionalities on the hydrophobic tails of the anion could alter precursor solubility and modify interactions with both the solvent and the QD surface.

To disentangle these effects, we developed a library of Cs precursors with systematic variation of both headgroups and tails (Scheme 1b). To maintain a coherent chemical framework, we focus exclusively on phosphoxo-based anions, which alone provide access to a wide range of Lewis basicities and coordination motifs owing to the different number and geometry of oxygen donor atoms in the phosphorus coordination sphere. The library spans dialkylphosphinates, which include the closest analogues of the commonly employed Cs-DOPA, as well as dialkylphosphates, monoalkylphosphinates, and monoalkylphosphonates. Organic substituents were varied in length and branching and, in selected cases, functionalized with aryl or oleyl groups. Most dialkylphosphinic, dialkylphosphoric, and monoalkylphosphinic acids were synthesized in-house (Scheme 1c),^36^ whereas monoalkylphosphonic acids were obtained commercially.

Given the large number and structural diversity of the precursors examined in this work, we avoid conventional initial-letter abbreviations and instead opt for a simplified acronym-based nomenclature that directly conveys key structural information. These acronyms specify: (i) the headgroup, where PA denotes phosphinates, Pate – phosphates, and POA – phosphonates; (ii) the number of alkyl substituents R, with prefix D indicating dialkyl-substituted salts and its absence denoting monoalkyl-substituted species; (iii) the length of the longest alkyl tail, encoded by the index *n* in the first alkyl tail C_*n*_; and (iv) alkyl tail branching, indicated by additional C_*n*_ segments within the tail (R) acronym, without specifying the branching position. For example, di-*n*-octylphosphinic acid is denoted DC_8_PA, while the commonly used bis(2,4,4-trimethylpentyl)phosphinic acid^31,32^ becomes DC_5_(C_1_)_3_PA; the corresponding Cs salts are Cs-DC_8_PA and Cs-DC_5_(C_1_)_3_PA. Salts bearing additional organic functionalities are assigned individual acronyms following the same nomenclature logic (*e.g.*, Cs-DPhEtPA for cesium bis(2-phenylethyl)phosphinate). The acronyms of all cesium precursors employed in this study are provided in Scheme 1b alongside the corresponding structures. Reaction conditions for all CsPbBr_3_ QD samples reported here and in SI are summarized in Table S1.

Remarkably, all tested Cs precursors enabled controlled growth of CsPbBr_3_ QDs. With the exception of precursors bearing aryl functionalities, which are discussed below, all systems exhibit a robust positive correlation between precursor concentration and final QD size, consistent with the trend previously observed for DOPA-based Cs precursors.^27^ Furthermore, variations in reaction yield remain modest (typically <30%) across the entire concentration range for each Cs precursor, although the absolute yields vary substantially between precursors (Table S1).

### The headgroup effect

We first examined the effect of the headgroup, as it can be expected to exert a strong influence on Cs release. Fig. 1a–c compare QD sizes obtained using Cs dialkylphosphinates and dialkylphosphates across three different alkyl substituents and several precursor concentrations, while analogous data for monoalkyl-substituted precursors are shown in Fig. S1–S4. Dialkylphosphinates and dialkylphosphates produce nearly identical QD sizes at matched concentrations and tail groups. This correspondence persists across the entire low-concentration regime (Cs precursor concentrations below 1.5 mM). Noticeable deviations and broader size dispersions emerge only at higher concentrations and only for precursors bearing small alkyl substituents, likely reflecting differences in their propensity to form micellar species, as discussed below. Not only the size, but also the size distribution and particle morphology remain largely unchanged at lower concentrations, as confirmed by the corresponding absorption spectra and TEM images (Fig. 1a–c). Consistent with this overall weak headgroup effect, monoalkylphosphinates Cs-RPA and monoalkylphosphonates Cs-RPOA likewise yield QDs of comparable sizes under equivalent conditions, although the difference is more coherent in this case, and Cs-C_12_POA systematically produced 0.3–0.9 nm larger QDs than Cs-C_12_PA (Fig. S1–S4). Nevertheless, across mono and didodecyl-substituted phosphinates (Cs-C_12_PA and Cs-DC_12_PA), phosphonate (Cs-C_12_POA), and phosphate (Cs-DC_12_Pate), the dependence of the obtained QD size on the concentration is nearly identical, as illustrated by the comparison of Fig. 1a, S1e–h, S2b, S3e–h and S4b. Taken together, these results indicate that the headgroup plays a minor role in determining the final QD size under the conditions explored here.

**Fig. 1:**
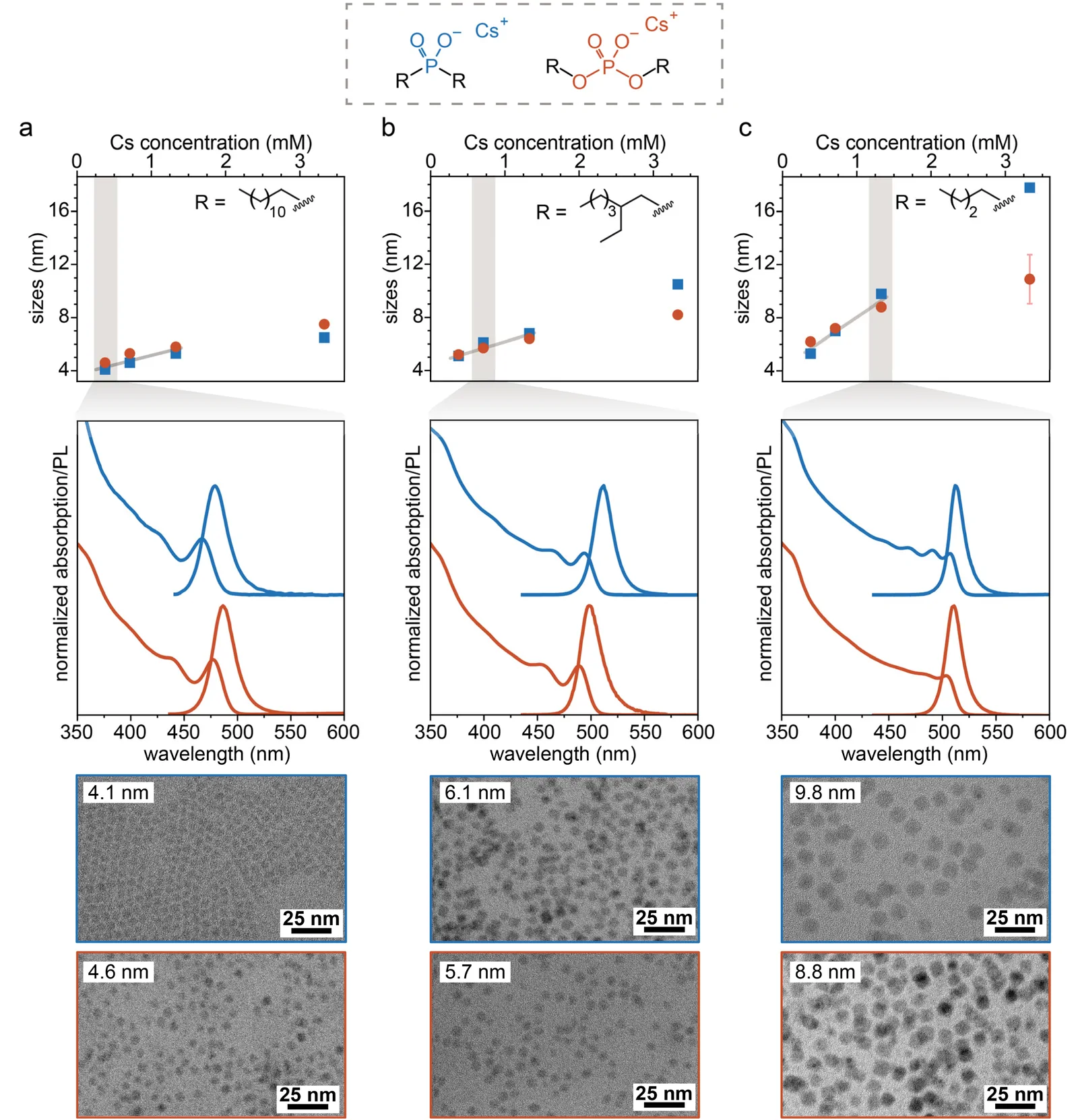
Effect of the headgroup of the Cs precursor on CsPbBr_3_ QDs synthesized with dialkylphosphinates and dialkylphosphates bearing different alkyl substituents: dodecyl (C_12_; a), bis-2-ethylhexyl (C_6_C_2_; b), and butyl (C_4_; c). The upper panels show the corresponding QD size plots as a function of Cs-precursor dilution. The pale brown shading highlights the dilution selected for the representative normalized absorption and photoluminescence (PL) spectra and the corresponding TEM images shown in the middle and bottom panels, respectively. Pale brown lines in the upper panels are included solely as guides to the eye and do not imply linear behavior; rather, they emphasize the similar QD sizes obtained with both headgroups, consistently observed at low precursor concentrations.

### The effect of alkyl tail length

In contrast, the nature of the tail imparts a pronounced and systematic influence on the synthesis. Across the precursors shown in Fig. 1, bulkier substituents consistently produce smaller QDs. They also markedly attenuate the dependence of QD size on precursor concentration: branched Cs-DC_6_C_2_PA/Cs-DC_6_C_2_Pate, and linear Cs-DC_12_PA/Cs-DC_12_Pate reduce the slope of the size-concentration relationship by more than twofold relative to the short-tail Cs-DC_4_PA/Cs-DC_4_Pate. For even longer-tail systems, such as Cs-DC_16_Pate, this dependence becomes effectively concentration-independent, yielding QDs of approximately 4.1–4.2 nm across the entire concentration range tested (Fig. S5 and S6). We therefore decided to separately investigate the effect of alkyl tail length and branching on QD growth.

We first examined a series of unbranched dialkylphosphinates Cs-DRPA with tail R lengths ranging from C_4_ to C_12_. Cesium dialkylphosphinates with longer alkyl tails have insufficient solubility in hexane, but we also included dihexadecylphosphate Cs-DC_16_Pate in the comparison. The earlier-identified trend, namely that longer alkyl tails yield smaller QDs, remains valid for precursors with C_8_ and longer tails (Fig. 2). For example, at a Cs concentration of 0.714 mM, the final QD size decreases systematically from 9.6 nm for DC_8_PA to 4.6 nm for DC_12_PA and 4.1 nm for DC_16_Pate; a similar trend is observed at 3.333 mM, where the QD size decreases from 14.4 nm for DC_8_PA to 6.5 nm for DC_12_PA and 4.2 nm for DC_16_Pate (Fig. 2, S5 and S6).

**Fig. 2:**
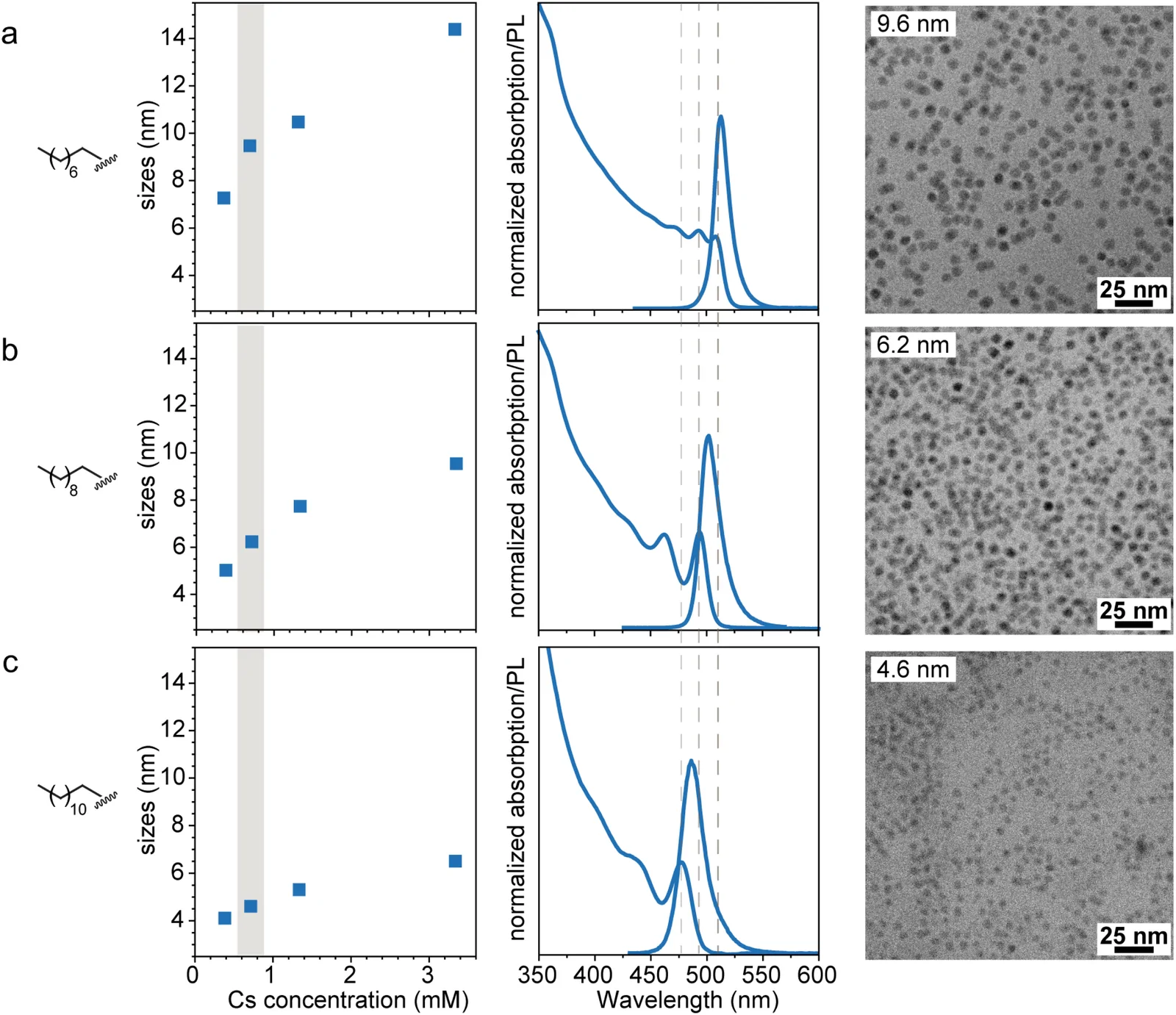
The size charts of CsPbBr_3_ QDs synthesized at different dilutions using dialkylphosphinates with different alkyl tail length: (a) Cs-*n*-dioctylphosphinate (Cs-DC_8_PA), (b) Cs-*n*-didecylphosphinate (Cs-DC_10_PA), and (c) Cs-*n*-didodecylphosphinate (Cs-DC_12_PA). The middle column shows the representative normalized absorption and photoluminescence (PL) spectra of CsPbBr_3_ QDs synthesized at fixed dilution (highlighted with the pale brown shading in the left column). The right column presents the corresponding TEM images.

Several factors could contribute to the observed reduction in QD size with increasing alkyl tail length. First, alkyl tails may alter precursor solubility in hexane or promote the formation of reverse micelles, both of which could influence Cs release kinetics and, in turn, the generation rate of the proposed intermediate.^27^ Second, dialkylphosphinates may act as QD surface ligands that compete with TOPO for QD surface sites. In this scenario, alkyl tail length could affect the nucleation rate or, independently, the subsequent growth kinetics, as tightly packed bulkier ligands can hinder the approach of reactive species to the QD surface.

To distinguish between these scenarios, we performed *in situ* absorption spectroscopy, which provides time-resolved information on nucleation, growth, QD concentration, and total reaction yield before QD purification. Heat map representations of the evolving absorption spectra for reactions employing Cs-DC_8_PA, Cs-DC_10_PA, and Cs-DC_12_PA (Fig. 3a) clearly resolve the shift of the excitonic peak, which is converted into a QD size using an established sizing curve.^37^ A selection of corresponding absorption spectra confirms largely featureless absorption at 375 nm (Fig. 3b). Monitoring the absorbance at this wavelength provides an estimate of the total QD volume in solution and, consequently, the QD concentration (see Section 4.4 in the SI for details). The resulting temporal traces of QD size and concentration are shown in Fig. 3c.

**Fig. 3:**
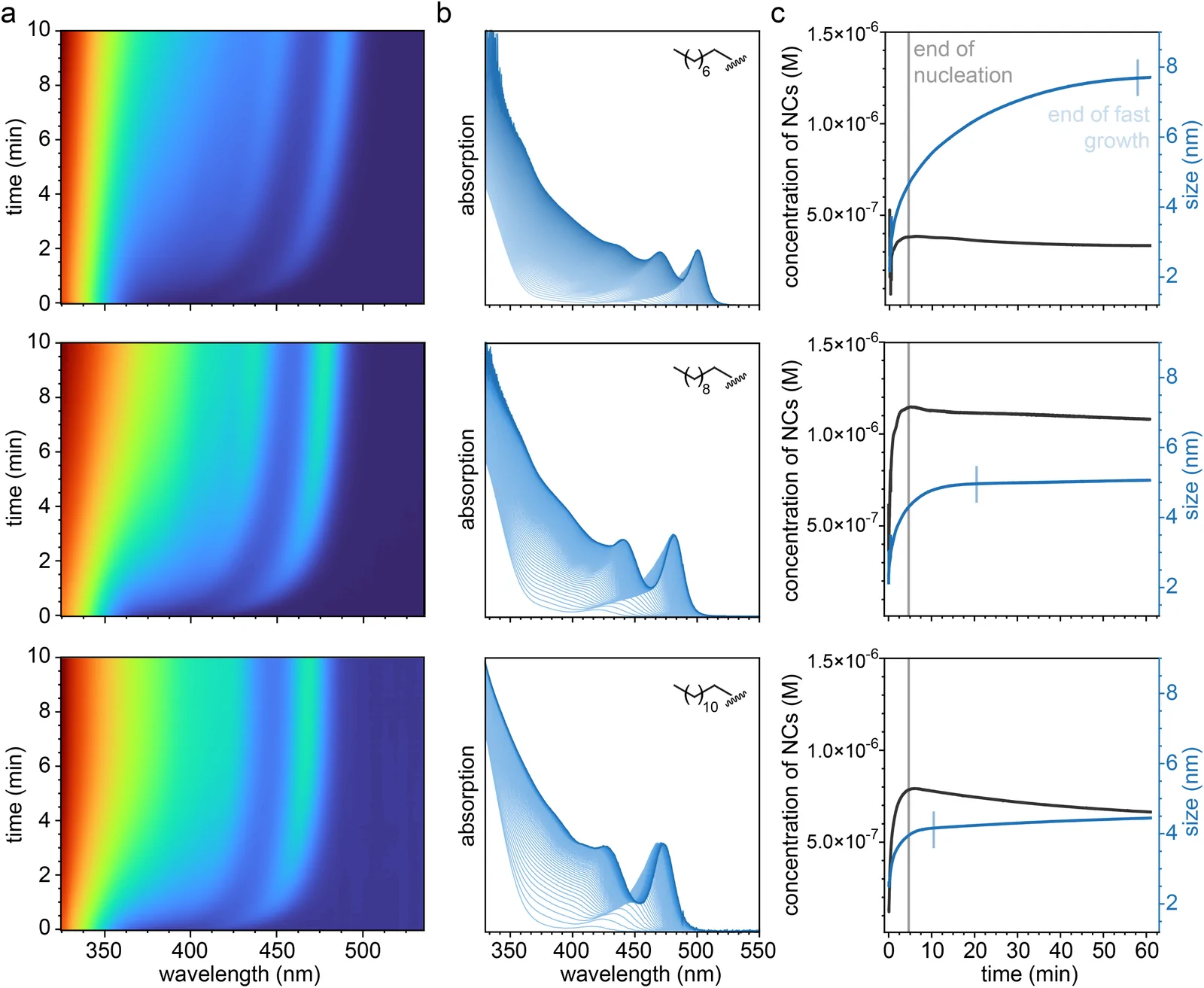
(a) Heat map representation of *in situ* recorded absorption spectra of CsPbBr_3_ QDs synthesized using Cs-*n*-dioctylphosphinate (Cs-DC_8_PA, top panel), Cs-*n*-didecylphosphinate (Cs-DC_10_PA, middle), and Cs-*n*-didodecylphosphinate (Cs-DC_12_PA, bottom panel). (b) Evolution of the absorption spectra of CsPbBr_3_ QDs during the first 10 min of the reaction. (c) Corresponding time-dependent QD concentration (black) and QD size (blue) during the 60 min synthesis of CsPbBr_3_ QDs. The grey vertical line indicates the end of nucleation, while the blue markers denote the end of the fast growth regime and the onset of ripening.

All three systems exhibit very similar nucleation behavior. They initially produce comparable nuclei concentrations of 0.1–0.4 µM, with excitonic peak positions between 400 and 415 nm, corresponding to nearly identical nuclei sizes of 2.2–2.5 nm. The nucleation time is also similar for all three samples, at approximately 5 minutes. Across all three Cs precursors, substantial amounts of precursor – corresponding to 38–75% of the injected quantity – remain unreacted at the end of nucleation.

A clear divergence between the systems emerges during the subsequent fast-growth phase. The end of this phase is defined here as the time at which the growth rate – the first derivative of the size evolution – drops and flattens (Fig. S7). Cs-DC_8_PA allows continued, albeit slow, growth throughout nearly the entire synthesis, whereas Cs-DC_10_PA supports growth for only about 13 min, and Cs-DC_12_PA transitions to Ostwald ripening within about 6 min after the end of nucleation. Consistent with these trends, the overall *in situ* reaction yield decreases from 97–100% for Cs-DC_8_PA and Cs-DC_10_PA to 39% for Cs-DC_12_PA. The combination of nearly identical nucleation behavior and markedly different growth kinetics and time across the Cs-DC_8_PA to Cs-DC_12_PA series – despite substantial precursor remaining in solution – indicates that monomer access to the QD surface differs between these systems. We attribute this effect to active binding of dialkylphosphinates at the QD surface, such that they function not only as precursors but also as surface ligands.^34,35^ To verify the possibility of such binding, the QDs were capped at the end of the synthesis with phosphorus-free ligands – a minimal amount of the strongly binding oleylguanidinium bromide ligand.^38^ This ligand enabled purification of the QDs from reaction byproducts and excess ligands without fully displacing the native surface species. ^31^P NMR of the purified QDs therefore probes only surface-bound species inherited from the reaction mixture and confirms the presence of both DC_12_PA and TOPO (Fig. S8). In this scenario of Cs-precursor serving also as a weakly-bound surface ligand, longer alkyl tails impose increasingly strong steric barriers to monomer incorporation, resulting in progressively smaller QDs and lower overall yields as tail length increases from C_8_ to C_16_ (Table S1).

A markedly different picture emerges for dialkylphosphinates bearing alkyl tails shorter than C_8_ – including those featuring branched tails – across all concentrations (Fig. 4a). Cs-DC_4_PA and Cs-DC_6_PA produce QDs that reach at most half of the size expected from extrapolation of the C_8_–C_16_ trend when synthesized at a Cs concentration of 0.377 mM. A direct comparison of Cs-DC_8_PA and Cs-DC_6_PA shows that the chemical yields remain comparable, at 99.7% and 98.9%, respectively. Despite this similarity, the final QD size decreases from 7.3 to 5.0 nm, accompanied by an almost threefold increase in the number of nuclei, from 5.6 × 10^15^ for Cs-DC_8_PA to 16.2 × 10^15^ for Cs-DC_6_PA, while reaction volume and precursor concentrations remain unchanged.

**Fig. 4:**
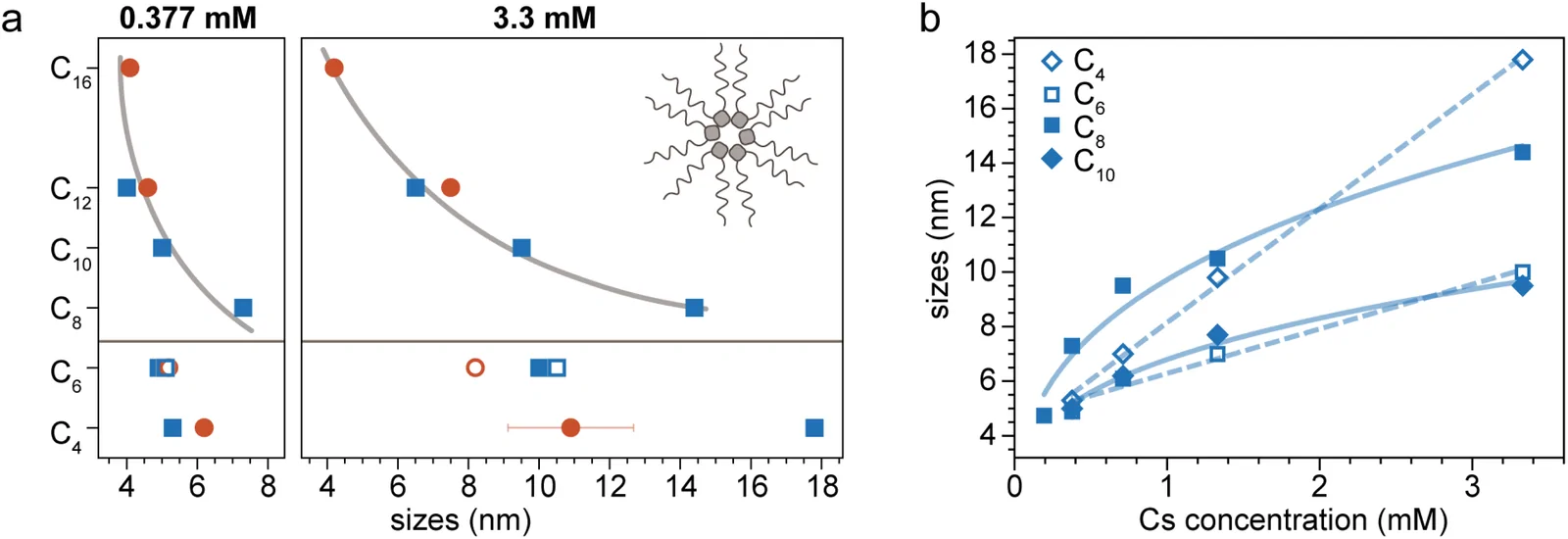
(a) Dependence of QD size on the alkyl chain length of Cs-DRPA (R = C_*n*_; blue squares) and Cs-DRPate (R = C_*n*_; orange circles) precursors at Cs concentrations of 0.377 mM (left) and 3.33 mM (right). Filled markers correspond to linear alkyl chains, while open markers denote branched chains with the same number of carbon atoms (*n*) in the longest chain. Both datasets exhibit a clear threshold in system behavior at a chain length of C_8_ (solid horizontal line). The pale brown curves are included as guides to the eye. (b) Dependence of QD size on Cs precursor concentration for Cs-DRPA with R = C_4_ and C_6_ (open symbols) and R = C_8_ and C_10_ (filled symbols). Pale blue lines are included as guides to the eye (dashed for C_4_ and C_6_; solid for C_8_ and C_10_). Nearly linear increase in QD size is observed for C_4_ and C_6_, whereas strong-to-mildly sublinear size scaling is found for longer alkyl substituents.

We attribute the threshold shown in Fig. 4a to changes in precursor organization in solution when transitioning from alkyl tails longer than C_8_ to shorter ones, potentially reflecting their reduced propensity to form reverse micelles. Alkylphosphinic and alkylphosphonic acids bearing tails longer than C_8_ carbons are known to form micelles in aqueous media.^39^ In apolar solvents, the double-tailed architecture of dialkyl-substituted salts gives rise to a wedge-shaped molecular geometry and an increased packing parameter, favoring reverse micelle formation.^40^ For example, Na-DC_8_PA has been reported to form reverse micelles at approximately 100 mM solution in isooctane.^41^ For Cs-DC_4_PA to Cs-DC_10_PA precursor solutions at synthetically relevant concentrations (3.3 mM), ^31^P diffusion-ordered NMR spectroscopy (DOSY NMR) confirms assembly into species with solvodynamic diameters 2–3 times larger compared to TOPO (Fig. S9). However, while precursors with longer alkyl tails mostly exist as reverse micelles, shorter-tailed analogues may coexist in equilibrium between assembled and molecularly dispersed forms. The latter are expected to improve Cs availability, promoting more intense nucleation and leaving fewer precursors available for subsequent QD growth – fully consistent with the observed behavior.

Short- and long-tail dialkylphosphinates show a different dependence of QD size on Cs precursor concentration. For tails shorter than C_8_, QD size scales linearly with Cs precursor concentration (Fig. 4b). In contrast, for longer alkyl tails, this dependence is markedly shallower, close to *d* ∼ *c*^1/3^, corresponding to volume rather than diameter scaling linearly with Cs precursor concentration. This difference demonstrates that precursors with alkyl tails shorter and longer than C_8_ operate under qualitatively different growth regimes, although the mechanistic origin of the observed scaling trends remains to be elucidated.

### The effect of alkyl tail branching

We next examined the influence of alkyl tail branching on the synthesis of CsPbBr_3_ QDs. Fig. 5a–c compare three Cs precursors with different branching patterns of the tail: Cs-DC_6_PA without branching, Cs-DC_6_C_2_PA bearing a single ethyl substituent, and Cs-DC_5_(C_1_)_3_PA bearing methyl and *tert*-butyl substituents. Remarkably, all three precursors yield nearly identical QDs and display the same near-linear size-concentration dependence. These observations indicate that the length of the longest alkyl segment, rather than the number and position of branching substituents, governs reaction kinetics in this regime.

**Fig. 5:**
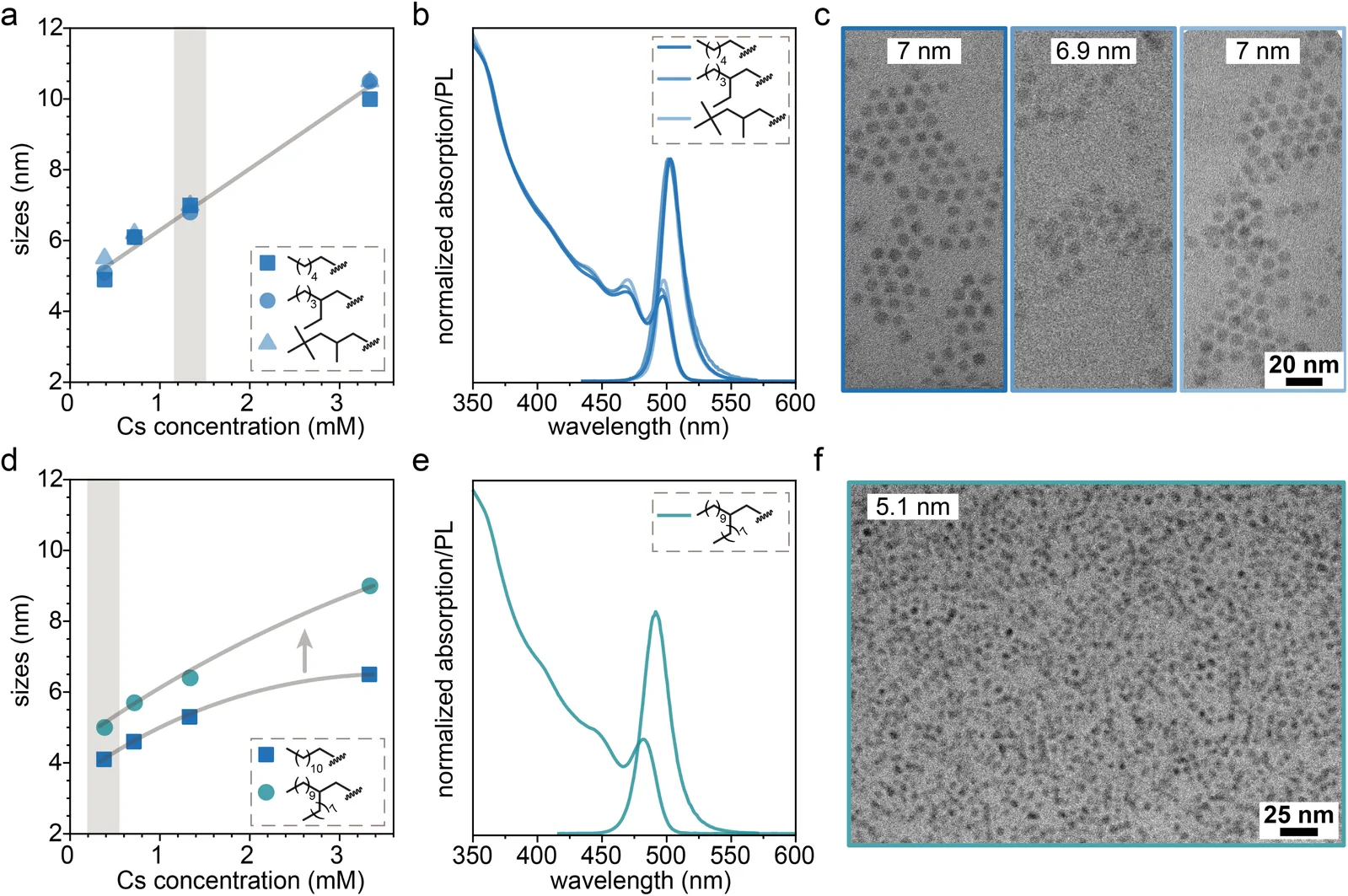
(a) The size charts of CsPbBr_3_ QDs synthesized at different dilutions using Cs dihexylphosphinate (Cs-DC_6_PA; dark blue square), Cs bis-2-ethylhexylphosphinate (Cs-DC_6_C_2_PA, turquoise circles), and Cs diisooctylphosphinate (Cs-DC_5_(C_1_)_3_PA, blue triangles). The pale brown line is included solely as a guide to the eye to highlight the similar QD size trends obtained with all three Cs precursors. The pale brown shading highlights the dilution used for the displayed spectra. (b) The representative normalized absorption and photoluminescence (PL) spectra of CsPbBr_3_ QDs synthesized at the same dilution (1.33 mM) using Cs-DC_6_PA (dark blue), Cs-DC_6_C_2_PA (turquoise), and Cs-DC_5_(C_1_)_3_PA (blue). (c) Corresponding TEM images of CsPbBr_3_ QDs synthesized at the same dilution (1.33 mM) using Cs-DC_6_PA, Cs-DC_6_C_2_PA, Cs-DC_5_(C_1_)_3_PA, respectively. (d) The size charts of CsPbBr_3_ QDs synthesized at different dilutions using Cs-DC_12_PA (dark blue square) and Cs bis-2-octyldodecylphosphinate (Cs-DC_12_C_8_PA; aquamarine circles). The pale brown shading highlights the dilution used for the displayed spectra. (e) The representative normalized absorption and PL spectra of CsPbBr_3_ QDs synthesized using Cs-DC_12_C_8_PA at 0.377 mM dilution. (f) Corresponding TEM image of CsPbBr_3_ QDs synthesized using Cs-DC_12_C_8_PA at 0.377 mM dilution.

For precursors bearing longer alkyl tails, branching exerts a pronounced influence. Introducing additional branching into the DC_12_PA backbone, as in Cs-DC_12_C_8_PA, results in notably larger QDs (Fig. 5d–f). Following the earlier observation of DC_12_PA tails crowding the surface of QDs, we attribute increased QD size in the case of DC_12_C_8_PA to more loose ligand packing at the QD surface in the regime where surface coverage governs both QD size and overall reaction yield. Accordingly, synthesis with Cs-DC_12_C_8_PA exhibits a substantially higher chemical yield than Cs-DC_12_PA (Table S1), indicating a diminished ability to arrest QD growth. In Table 1, we summarize the key distinctions in QD growth behavior for Cs-DRPA precursors bearing alkyl tails shorter and longer than C_8_.

**Table 1:** The key qualitative distinctions in QD growth with Cs dialkylphosphinate precursors Cs-DRPA

R shorter than C_8_	R equal to or longer than C_8_
QD growth is limited by the amount of precursor	QD growth is limited by the Cs precursor access to the QD surface
The yield does not change substantially as the tail R length increases	The yield decreases as the tail R length increases
QD size does not change substantially with introducing branching into the tail R	QD size increases with introducing branching into the tail R
QD size scales with [Cs]	QD volume scales with [Cs]

### Alkyl tails with aryl and oleyl groups

The correlations discussed above apply exclusively to Cs precursors bearing saturated alkyl substituents. Incorporation of additional functional groups into the tail R alters QD growth behavior.

One example is the oleyl-substituted precursor Cs-DOlPA (Fig. S10–S12). Despite possessing the longest tails among the precursors investigated in this study, the presence of the *Z*-unsaturated oleyl chains promotes loose and permeable packing of the ligand shell due to increased conformational disorder.^42^ Accordingly, Cs-DOlPA yields QDs that are substantially larger and obtained in a higher overall yield compared to saturated long-tail Cs-DRPAs. Still, a well-pronounced positive correlation between QD size and precursor concentration is observed, and this correlation remains sublinear, consistent with the behavior characteristic of long-chain Cs-DRPAs.

Another example is the incorporation of aromatic groups into the ligand tail. Such precursors were soluble only in aromatic solvents (*e.g*. toluene and mesitylene), thereby drastically altering precursor solubility and the overall reaction environment. Although QD synthesis remained feasible with these precursors, it consistently yielded more polydisperse QDs with sizes of 6.4–10 nm, largely independent of substituent, headgroup, or even the tested precursor concentration (Fig. S13–S16).

### Synthesis of small CsPbBr_3_ and FAPbBr_3_ QDs

Having established that the tail length of alkyl-substituted phosphoxo Cs salts provides a powerful handle for controlling the size of CsPbBr_3_ QDs, we next explore whether this strategy can be transferred to hybrid FAPbBr_3_ QDs and to push the lower size limits for CsPbBr_3_.

Although Cs-DOPA, the established Cs precursor in TOPO-modulated syntheses, can yield CsPbBr_3_ QDs as small as 3.1 nm,^27^ such sizes are reached only under extreme dilution. This renders the synthesis highly sensitive to small variations in reaction conditions and limits productivity to approximately 20 µg of QDs per mL of solvent, so that preparing practical quantities requires scale-up to the liter scale.

To achieve similar QD sizes without relying on extreme dilution, we use the Cs precursor with the longest purely alkyl tail examined in this work which exhibits adequate solubility – Cs-DC_16_Pate. Using this precursor, we obtained CsPbBr_3_ QDs with a size of 3.0 nm (438 nm excitonic peak position) at an 87% chemical yield after 10 s of reaction, producing approximately 10 mg of QDs in a 15 mL reaction batch (Fig. S17). This corresponds to nearly 40 times as much material per solution volume as using Cs-DOPA. Furthermore, even such small QDs exhibit bright (up to 90%) photoluminescence quantum yields (PL QY) and have a narrow size distribution with a half-width at half maximum (HWHM) of 13 nm (82 meV). Those HWHM values are not achievable for such small QDs when using a conventional Cs-DOPA-based precursor and relying on extreme dilutions. Even smaller sizes, with the excitonic peak position down to approximately 380 nm (3.26 eV), are accessible with *in situ* absorption spectroscopy using the Cs-DC_16_Pate precursor (Fig. S18). None of the existing sizing curves cover such a high-energy range for CsPbBr_3_ QDs. Instead, we extrapolate the semi-empirical relation reported by Aubert *et al.*,^43^ towards smaller sizes and estimate QD size at the earliest stage of growth to be about 2.0–2.5 nm, *i.e.* as small as 3–4 PbBr_6_ octahedra in width. Shorter-tailed precursor Cs-DC_12_PA can also provide access to small isolable QDs (3.1 nm, excitonic peak at 444 nm), with a similarly narrow size distribution, albeit at approximately twofold higher dilution and with lower chemical yield (Fig. S19).

Next, we transferred our precursor library to the synthesis of hybrid FAPbBr_3_ QDs (Fig. 6a). This hybrid composition exhibits faster growth than its Cs-based counterpart. Despite substantial advances achieved with the TOPO-modulated approach, the preparation of small FAPbBr_3_ QDs still requires substantial scale-up, precise early reaction quenching, and the addition of a secondary ligand such as oleic acid to obtain practical quantities, which – similar to the CsPbBr_3_ QDs – remain limited to ∼18 µg of QDs per mL of solvent. As a result, the reproducible synthesis of monodisperse FAPbBr_3_ QDs below 6 nm in practical amounts remains particularly challenging.

**Fig. 6:**
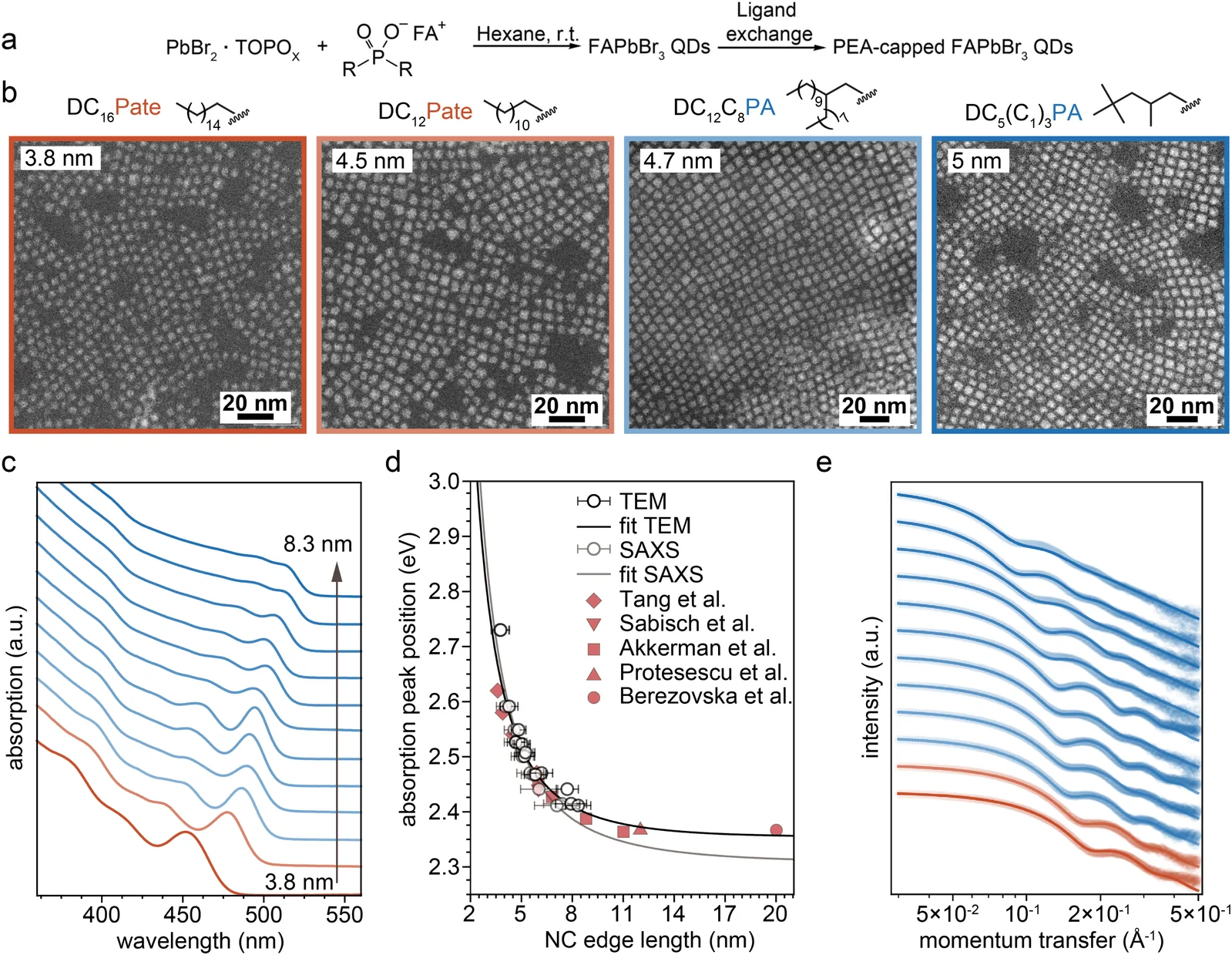
(a) Reaction scheme for the synthesis of FAPbBr_3_ QDs. (b) Dark-field STEM images of FAPbBr_3_ QDs synthesized at the same dilution using FA-DC_16_Pate, FA-DC_12_Pate, FA-DC_12_C_8_PA and FA-DC_5_(C_1_)_3_PA. (c) Optical absorption spectra of FAPbBr_3_ QDs with sizes ranging from 3.8 to 8.3 nm. For clarity, a cumulative vertical offset was applied to successive spectra. (d) Size-dependent absorption peak position for FAPbBr_3_ QDs as a function of QD size determined by TEM (black) and SAXS (pale brown). Solid lines represent the fit to the semi-empirical expression from ref. 43 using both experimental data (open circles, with error bars indicating standard deviations) and literature data (orange filled markers)^20,27,38,45,46^ for the TEM dataset and experimental data (open circles, with error bars indicating standard deviations) for the SAXS dataset. (e) Experimental SAXS patterns of FAPbBr_3_ QDs (semi-transparent circles) together with fits based on a Gaussian monomodal size distribution (solid lines). A cumulative vertical offset was applied to the successive patterns.

We found that dialkylphosphinates and dialkylphosphates provide effective size control for FAPbBr_3_ QDs, even in the absence of oleic acid. As expected from the CsPbBr_3_ case, precursors bearing longer alkyl tails produce smaller QDs (Table S2 and Fig. S20, S21). Introducing the longest-tail precursors FA-DC_16_Pate and FA-DC_12_Pate yields monodisperse FAPbBr_3_ QDs with sizes of 3.8 nm and 4.5 nm, respectively (Fig. 6b and S20), with an approximate yield of 0.15 mg QDs per mL solvent (as estimated using the extinction coefficient of CsPbBr_3_ QDs).

The extended range of FAPbBr_3_ QD sizes, accessible within a unified synthetic framework in sufficient quantities, with similarly narrow size distribution and morphologies, enabled the construction of a sizing curve, analogous to those established for other QD compositions.^44^ To this end, we synthesized a series of FAPbBr_3_ QDs with edge lengths ranging from 3.8 to 8.3 nm. For each sample, QD size was determined from TEM images and independently assessed in solution by small-angle X-ray scattering (SAXS). These values were correlated with the position of the first excitonic absorption peak, defined as the minimum in the second derivative of the absorption spectrum (Fig. 6c–e, S22–S26 and Table S3). For comparison, TEM sizing data from ref. 20, 27, 38, 45 and 46 are included in Fig. 6d, showing good agreement across datasets. The full TEM and SAXS datasets were fitted using the recently proposed semi-empirical relation.^43^ As our FAPbBr_3_ QDs adopt a cuboidal morphology, we apply a semi-empirical fitting model tailored for this shape, which can be readily converted to the spherical case using the scaling factor reported in ref. 43 (Fig. S27). Only the bandgap and Bohr diameter were treated as fitting parameters, whereas the dielectric constant was fixed during the fitting procedure. We note that the reported dielectric constant varies slightly depending on the experimental technique, with values ranging from 6.0 to 8.6.^47,48^ Fitting performed using both limiting values yielded nearly identical results, indicating that the fitting outcome is largely insensitive to the exact dielectric constant within this range. The resulting fitting parameters for both datasets (TEM and SAXS) are summarized in Table S4 and are in good agreement with literature values.^47,49^ This semi-empirical relation thus provides a reliable and practically useful means of directly extracting QD size from the energy of the first excitonic absorption peak over a broad size range and opens the way for *in situ* absorption spectroscopy studies of FAPbBr_3_ QDs nucleation and growth.

## Conclusions

This study established the molecular design of A-site cation precursors as a decisive parameter in the synthesis of lead bromide perovskite quantum dots (QDs). Through systematic variation of phosphoxo anion headgroups and the identity of the tails of dialkylphosphinate and dialkylphosphate Cs salts, we show that the headgroup has no systematic influence on the QD size, while precursor aggregation – likely in the form of reverse micelles – significantly influences growth of QDs. An additional factor emerges for precursors bearing alkyl tails longer than C_8_: increasing tail length modulates precursor access to the QD surface, enabling the synthesis of progressively smaller QDs. This trend persists across dilution regimes and headgroup types, enabling the synthesis of isolable CsPbBr_3_ QDs as small as 3.0 nm at 1 mg for 1 mL of the reaction solution, while maintaining a narrow size distribution (with excitonic absorption HWHM 13 nm) and optical quality (90% PLQY). Even smaller species, down to approximately 3–4 PbBr_6_ octahedra in width, are accessible with *in situ* absorption spectroscopy, opening opportunities to interrogate their formation mechanism directly.

The same steric modulation of Cs precursor structure was successfully transferred to hybrid FAPbBr_3_ QDs, enabling access to isolable QDs with sizes as small as 3.8 nm without extreme dilution or secondary ligands. The extended size range achieved within a unified synthetic framework enabled the construction of a reliable sizing curve, allowing FAPbBr_3_ QD sizes to be determined from the excitonic absorption peak over the 3.8–20 nm range.

## Author contributions

Conceptualization: Y. B., D. N. D., M. V. K.; methodology: Y. B., D. N. D.; investigation: Y. B., V. S., N. R., B. A., S. S.; visualization: Y. B., B. A., D. N. D.; funding acquisition: D. N. D., M. V. K.; resources: M. V. K.; supervision: D. N. D., M. V. K.; writing – original draft: Y. B., D. N. D.; writing – review & editing: Y. B., D. N. D., M. V. K.

## Conflicts of interest

There are no conflicts to declare.

## Supplementary Material

SC-017-D6SC05095F-s001

## Data Availability

Materials, synthesis details, experimental methods, and supplementary figures are available in the supplementary information (SI). Raw data, including unprocessed spectra, characterization files, and analysis data sets, are available at https://doi.org/10.5281/zenodo.21458682. Supplementary information is available. See DOI: https://doi.org/10.1039/d6sc05095f.
