# Supplementary material for: Alkyl tails of cesium and formamidinium precursors control the growth of lead bromide perovskite nanocrystals
Source: Chem Sci. 2026 Sep 15;17(43):20998–1009. doi: 10.1039/d6sc05095f (PMC13635994; doi:10.1039/d6sc05095f)
Supplement: Supplementary file 1 [file SC-017-D6SC05095F-s001.pdf]

Supplementary Information (SI) for Chemical Science.
This journal is © The Royal Society of Chemistry 2026

Supplementary Information for

Alkyl Tails of Cesium and Formamidinium Precursors Control the Growth of Lead Bromide Perovskite Nanocrystals

Yuliia Berezovska,[a, b] Valerie Sekula,[a] Nikita Romanyuk,[a] Benjamin Aymoz,[a, b] Sebastian Sabisch,[a, b] Maksym V. Kovalenko*[a, b] and Dmitry N. Dirin*[a, b]

[a] Laboratory of Inorganic Chemistry, Department of Chemistry and Applied Biosciences, ETH Zürich, CH-8093 Zürich, Switzerland

[b] Laboratory for Thin Films and Photovoltaics, Empa−Swiss Federal Laboratories for Materials Science and Technology, CH-8600 Dübendorf, Switzerland

Contents

Contents 2

1. Chemicals 3

2. Synthesis of alkylphosphinic and alkylphosphonic acids 4

  2.1. Synthesis of monoalkylphosphinic acids 4

  2.2 Synthesis of dialkylphosphinic acids 4

  2.3. Synthesis of didodecyl hydrogen phosphate ($DC_{12}Pate$) 7

3. Synthesis of lead bromide perovskite QDs 8

  3.1. Synthesis of C_8C_{12}-PEA ligand 8

  3.2. Synthesis of OGB ligand 8

  3.3. Synthesis of $CsPbBr_3$ QDs 8

  3.4. Synthesis of $CsPbBr_3$ QDs capped with oleylguanidinium bromide (OGB) 10

  3.5. Synthesis of $FAPbBr_3$ QDs 10

4. Characterization methods 11

  4.1. UV-Vis optical absorption spectroscopy 11

  4.2. Photoluminescence (PL) and absolute PL Quantum Yield (QY) measurements 11

  4.3. Electron Microscopy 11

  4.4. *In situ* Absorption spectroscopy 11

  4.5. Mass Spectrometry (MS) 12

  4.6. Nuclear Magnetic Resonance Spectroscopy (NMR) 12

  4.7. Small-angle X-ray scattering (SAXS) and wide-angle X-ray scattering (WAXS) 12

5. Supporting figures 14

6. Supporting tables 33

7. Supporting information references 39

1. Chemicals

All chemicals were used as supplied without further purification.

1-Bromobutane (99%), 1-dodecanol (98%),1-dodecene (>=99.0%), 2-aminoethan-1-ol (>=99.0%), acetic acid (>99.8%), acetone (>99.8%), acetonitrile (**MeCN**, >99.9% HPLC LiChrosolve gradient grade), bis(2-ethylhexyl) phosphate (**DC_6C_2Pate**; 97%), cesium carbonate (Cs_2CO_3; 99.9%), cyclohexane, dichloromethane (>99.8% HPLC+Amylene), diphenylphosphinic acid (**DPhPA**; >=98.0%), ethanol absolut (>99.8%), ethyl acetate (>99.7%), hexane (>97% HPLC), magnesium turnings (>=99.5% for Grignard reactions), methanol (>99.9% HPLC gradient grade), oleyl alcohol (85%), phosphorus (V) oxychloride (**$POCl_3$**; 99%), potassium hydroxide (KOH; 90%), sodium hypophosphite monohydrate (99%), sodium sulfate (**Na_2SO_4**; ACS reagent, >=99.0%, anhydrous, powder), tetrabromomethane (99%), tetrahydrofuran (**THF**; >=99.9%, suitable for HPLC, inhibitor-free), toluene anhydrous (99.8%), triethylamine (**Et_3N**; 99%), dihexadecyl hydrogen phosphate (**DC_{16}Pate**; 97%), and triphenylphosphine oxide (98%) were purchased from **Sigma – Aldrich**; 2-ethylhexylbromide (>97.0%) and bis(2,4,4-trimethylpentyl) phosphonic acid (**DOPA** or **$DC_5(C_1)_3$PA**) were purchased from **Fluorochem**; 1-hexene (99%), potassium hydrogen sulfate (**$KHSO_4$**, 99%), triethylborane (1 M in THF), 1-bromohexane (99+%) were purchased from **Acros**; formamidine acetate (**FAOAc**; 99%) was purchased from **abcr**; 1-bromododecane (>98.0% (GC)), Dibenzyl Phosphate (**DBnPate**, >98.0%(T)(HPLC)) and Dibutyl Phosphate (**DC_4Pate**, >97.0% (T)) were purchased from **TCI**; pyridine was purchased from **Merck**; (2-bromoethyl)benzene (98%), 1,2-dibromoethane, 1-bromohexadecane (97%), 1-bromooctane (99%) and 2-octyl-1-dodecanol (97%) were purchased from **Aldrich**; 1-octylphosphonic acid (**C_8POA**, 98%) and oleic acid (**OAc**, 90%) were purchased from **AlfaAesar**; n-dodecylphosphonic acid (**C_{12}POA**, 99%) was purchased from **BLDpharm**; hydrochloric acid (HCl, fuming >37% EMSURE) from **VWR**; trioctylphosphine oxide (**TOPO**, 90%) and oleylamine (OAm, 95%) from **Strem Chemicals**; 9-(Bromomethyl)nonadecane, 98%, benzene-d_6 (C_6D_6, 99.5 atom% D), and dimethylsulfoxide-d_6 (**DMSO-d_6**, 99.8 atom% D) were purchased from **Apollo Scientific**; chloroform-d (**$CDCl_3$**, 99.8 atom% D), dichloromethane (**DCM**, 99.8% Extra Dry over MS), diethyl ether (**Et_2O**, 99.5%, Extra Dry over Molecular Sieve, Stabilized, AcroSeal®) and tetrahydrofuran (**THF**, 99.5%, Extra Dry over Molecular Sieve, Stabilized, AcroSeal®) were purchased from **Fisher Scientific;** lecithin (from soy, >97%) and n-octane (≥99%) was purchased from **Roth**; ethanol absolute and 1-bromodecane purum (>=97.0% GC) were purchased from **Fluka**; S-ethylisothiourea hydrobromide was purchased from **TCI;.** $PbBr_2$ was synthesized in house.

2. Synthesis of alkylphosphinic and alkylphosphonic acids

2.1. Synthesis of monoalkylphosphinic acids

Synthesis was performed as described elsewhere.[1]

Sodium hypophosphite hydrate (0.09 mol, 2.5 equiv.), the corresponding 1-alkene (0.036 mol, 1.0 equiv.), methanol (125 mL), and a 1 M solution of triethylborane in tetrahydrofuran (0.036 mol, 1.0 equiv.) were mixed in a round-bottom flask and stirred vigorously for 3 h under ambient conditions. The reaction mixture was subsequently transferred to a separatory funnel, and DCM (125 mL) together with a 1 M aqueous solution of $KHSO_4$ (125 mL) was added. The aqueous phase was extracted once more with DCM (125 mL). The combined organic layers were dried over Na_2SO_4, filtered, and evaporated on a rotary evaporator at 40°C.

For the liquid n-hexylphosphinic acid (**C_6PA**), the crude product was first converted into the potassium salt by adding a stoichiometric amount of potassium hydroxide in ethanol (0.5 M). After stirring for 1 h, the solvent was removed under reduced pressure. The resulting salt was recrystallized twice from a hot mixture of hexane (10 mL), tetrahydrofuran (10 mL), and ethanol (1 mL). The solution was allowed to cool slowly to room temperature, then refrigerated overnight in a freezer. The precipitated solid was collected by filtration. To obtain the final purified *n-hexylphosphinic acid*, the purified potassium *n*-hexylphosphinate salt was acidified with concentrated HCl (2 mL, 37%) in water (5 mL) and extracted three times with DCM (5 mL). The combined organic extracts were dried over Na_2SO_4, filtered, and evaporated on a rotary evaporator at 40°C. The resulting product was dried overnight under a vacuum (5 mbar) at room temperature.

n-Hexylphosphinic Acid (**C_6PA**). Yield: 42%. Appearance: colorless liquid. ^{1}H NMR (300 MHz, $CDCl_3$) δ, ppm (J, Hz): 9.45 (s, 1H), 8.00 (t, J = 1.8 Hz, 0.5H), 6.20 (t, J = 1.8 Hz, 0.5H), 1.75 (quin, J = 8.5 Hz, 2H), 1.65–1.45 (m, 2H), 1.35–0.95 (m, 6H), 0.85 (t, J = 7.0 Hz, 3H). ^{31}P NMR (75 MHz, $CDCl_3$): δ 38.7 ppm. Mass spectrum (m/z): $[M+H]^+$ calcd for $C_6H_{16}O_2P$, 151.09; found, 151.1.

For the solid n-dodecylphosphinic acid (**C_{12}PA**), the resulting crude product was recrystallized twice from hot hexane (25 mL) to obtain the purified product as a white solid.

n-Dodecylphosphinic Acid (**C_{12}PA**). Yield: 72%. Appearance: white powder. ^{1}H NMR (300 MHz, $CDCl_3$) δ, ppm (J, Hz): 9.45 (s, 1H), 8.00 (t, J = 1.8 Hz, 0.5H), 6.20 (t, J = 1.8 Hz, 0.5H), 1.75 (quin, J = 8.5 Hz, 2H), 1.60–1.35 (m, 2H), 1.41–0.95 (m, 18H), 0.85 (t, J = 7.0 Hz, 3H). ^{31}P NMR (75 MHz, $CDCl_3$): δ 39.3 ppm. Mass spectrum (m/z): $[M+H]^+$ calcd for $C_{12}H_{27}O_2P$, 235.182; found 235.181

2.2 Synthesis of dialkylphosphinic acids

Preparation of Grignard reagent. Synthesis was performed as described elsewhere.[2]

In an oven-dried three-necked round-bottom flask equipped with a magnetic stirrer, reflux condenser, and constant-pressure dropping funnel, magnesium turnings (0.041 mol, 1.5 equiv) were placed and covered with anhydrous Et_2O (5 mL). A solution of alkyl bromide (0.027 mol, 1 equiv) in anhydrous Et_2O (20 mL) was prepared separately and transferred to the dropping funnel. Approximately one-tenth of this solution was added to the magnesium suspension to initiate the reaction. The mixture was gently heated to 45°C until initiation of the Grignard reaction. After approximately 10 minutes, the remaining alkyl bromide solution was added dropwise under continuous reflux. Upon completion of the addition, the reaction mixture was maintained under reflux for an additional 2 hours to ensure complete conversion. The resulting solution was then transferred to the subsequent reaction step via cannula transfer.

Preparation of dialkylphosphinic acids. Synthesis was performed as described elsewhere.[3]

To an oven-dried two-neck flask, fitted with a reflux condenser, were added anhydrous Et_2O (30 mL) and $POCl_3$ (0.0135 mol, 0.5 equiv). The solution was cooled in an ice-salt mixture, and Grignard reagent (1 equiv) was slowly added. After stirring with cooling for 30 min, the reaction mixture was brought to r.t. and stirred overnight. The resulting mixture was then cooled again in an ice-salt mixture, and H_2O (10 mL) and pyridine (0.03 mol, 1.1 equiv) were slowly added. Stirring with cooling for 5 min was followed by reaction at r.t. for 7 h. The crude mixture was then poured into CH_2Cl_2 (50 mL), washed three times with diluted HCl (1–2%, 100 mL in total), dried over Na_2SO_4, filtered, and evaporated to dryness. The resulting crude product was purified by recrystallization from hot hexane (10 mL), filtered, and washed with hexane, providing the pure compound as a white solid. The resulting product was dried overnight under a vacuum (5 mbar) at 50 °C.

The following acids were prepared according to this method.

Dibutylphosphinic acid (**DC$_4$PA**). Yield: 63%. Appearance: white powder. ^{1}H NMR (300 MHz, $CDCl_3$) δ, ppm (J, Hz): 10.74 (s, 1H), 1.74 – 1.50 (m, 8H), 1.41 (h, *J* = 7.1 Hz, 4H), 0.92 (t, *J* = 7.3 Hz, 6H). ^{31}P NMR (121 MHz, $CDCl_3$) δ, ppm: 60.20. Mass spectrum (m/z): $[M+H]^+$ calcd for $C_8H_{20}O_2P$, 179.1195; found 179.1193.

Dihexylphosphinic acid (**DC$_6$PA**). Yield: 92%. Appearance: white powder. ^{1}H NMR (300 MHz, $CDCl_3$) δ, ppm: 9.68 (s, 1H), 1.75 – 1.50 (m, 8H), 1.46 – 1.21 (m, 12H), 0.94 – 0.83 (m, 6H). ^{31}P NMR (121 MHz, $CDCl_3$) δ, ppm: 60.51. Mass spectrum (m/z): $[M+H]^+$ calcd for $C_{12}H_{28}O_2P$, 235.1814; found 235.1813.

Dioctylphosphinic acid (**DC$_8$PA**). Yield: 89%. Appearance: white powder. ^{1}H NMR (300 MHz, $CDCl_3$) δ, ppm: 8.86 (s, 1H), 1.75 – 1.49 (m, 8H), 1.43 – 1.19 (m, 20H), 0.93 – 0.82 (m, 6H). ^{31}P NMR (121 MHz, $CDCl_3$) δ, ppm: 60.65. Mass spectrum (m/z): $[M+H]^+$ calcd for $C_{16}H_{36}O_2P$, 291.2447; found 291.2443.

Didecylphosphinic acid (**DC$_{10}$PA**). Yield: 74%. Appearance: white powder. ^{1}H NMR (300 MHz, $CDCl_3$) δ, ppm: 9.78 (s, 1H), 1.75 – 1.50 (m, 8H), 1.44 – 1.18 (s, 28H), 0.93 – 0.83 (m, 6H). ^{31}P NMR (121 MHz, $CDCl_3$) δ, ppm: 60.66. Mass spectrum (m/z): $[M+H]^+$ calcd for $C_{20}H_{44}O_2P$, 347.3073; found 347.3072

Didodecylphosphinic acid (**DC$_{12}$PA**). Yield: 58%. Appearance: white powder. ^{1}H NMR (300 MHz, $CDCl_3$) δ, ppm: 9.88 (s, 1H), 1.74 – 1.49 (m, 8H), 1.42 – 1.20 (m, 36H), 0.93 – 0.83 (m, 6H). ^{31}P NMR (121 MHz, $CDCl_3$) δ, ppm: 60.83. Mass spectrum (m/z): $[M+H]^+$ calcd for $C_{24}H_{52}O_2P$, 403.3699; found 403.3689.

Dihexadecylphosphinic acid (**DC$_{16}$PA**). Yield: 35%. Appearance: white powder. ^{1}H NMR (300 MHz, $CDCl_3$) δ, ppm: 10.23 (s, 1H), 1.80 – 1.50 (m, 8H), 1.44 – 1.06 (m, 52H), 0.95 – 0.81 (m, 6H). ^{31}P NMR (121 MHz, $CDCl_3$) δ, ppm: 61.35. Mass spectrum (m/z): $[M+Na]^+$ calcd for $C_{32}H_{67}NaO_2P$, 537.4771; found 537.4770.

Bis(2-ethylhexyl)phosphinic acid (**DC$_6$C$_2$PA**). The residue was purified by column chromatography using hexane/EtOAc (2:1, v/v), followed by elution of the product with EtOAc. Yield: 56%. Appearance: colorless liquid. ^{1}H NMR (300 MHz, $CDCl_3$) δ, ppm: 10.11 (br s, 1H), 1.94 – 1.10 (m, 22H), 1.02 – 0.75 (m, 12H). ^{31}P NMR (121 MHz, $CDCl_3$) δ, ppm: 63.09. Mass spectrum (m/z): $[M+H]^+$ calcd for $C_{16}H_{36}O_2P$, 291.2447; found 291.2446.

Bis(2-octyldodecyl)phosphinic acid (**DC$_{12}$C$_8$PA**). The residue was purified by column chromatography using hexane/EtOAc (2:1, v/v), followed by elution of the product with EtOAc. Yield: 64%. Appearance: colorless liquid. ^{1}H NMR (300 MHz, $CDCl_3$) δ, ppm: 9.78 (s, 1H), 1.93 – 1.14 (m, 70H), 0.97 – 0.81 (m, 12H). ^{31}P NMR (121 MHz, $CDCl_3$) δ, ppm: 61.03. Mass spectrum (m/z): $[M+H]^+$ calcd for $C_{40}H_{84}O_2P$, 627.6203; found 627.6205.

Bis(2-phenylethyl)phosphinic acid (**DPhEtPA**). Yield: 74%. Appearance: white powder. ^{1}H NMR (300 MHz, $CDCl_3$) δ, ppm: 8.55 (s, 1H), 7.36 – 7.12 (m, 10H), 3.01 – 2.87 (m, 4H), 2.11 – 1.96 (m, 4H). ^{31}P NMR (121 MHz, $CDCl_3$) δ, ppm: 58.07. Mass spectrum (m/z): $[M+H]^+$ calcd for $C_{16}H_{20}O_2P$, 275.1195; found 275.1194.

Preparation of bis[(Z)-octadec-9-enyl]phosphinic acid (dioleylphosphinic acid, DOlPA). Synthesis was performed in multiple steps according to the Scheme S1.

Scheme S1. Synthesis of bis[(Z)-octadec-9-enyl]phosphinic acid (dioleylphosphinic acid, DOlPA)

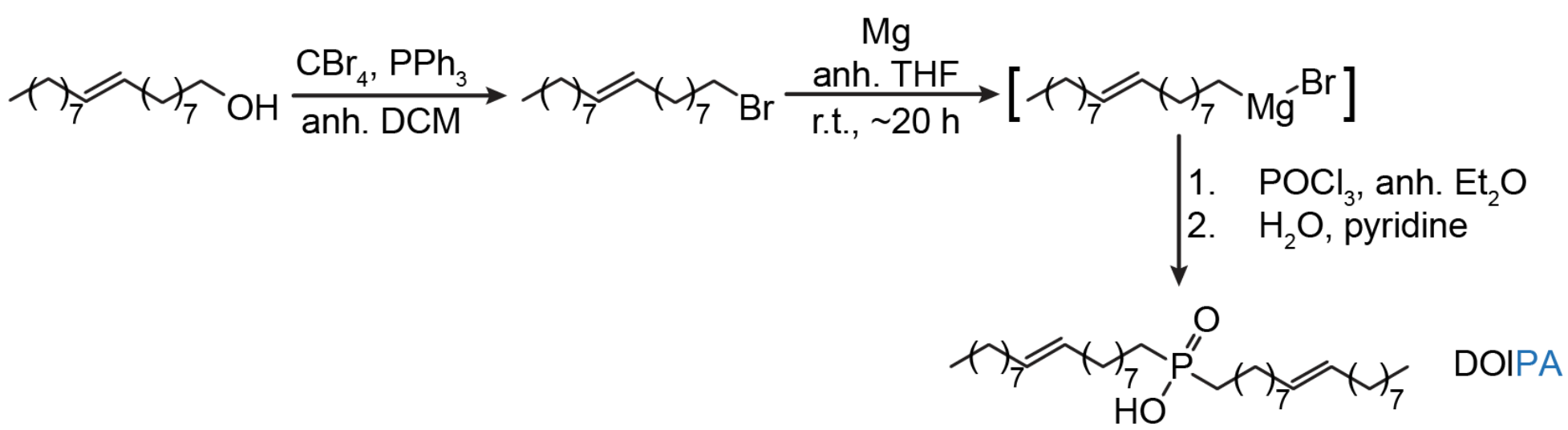

Step1. Synthesis of (Z)-octadec-9-en-1-yl bromide (Oleyl Bromide). Oleyl bromide was synthesized according to the procedure described elsewhere.[4]

A 250 mL flask was charged with oleyl alcohol (50 mmol, 1.0 equiv.), tetrabromomethane (55 mmol, 1.1 equiv.), and anhydrous DCM (50 mL), and the mixture was cooled to 0 °C using an ice bath. Triphenylphosphine (60 mmol, 1.2 equiv.) was then added portionwise over 15 min. After the addition was complete, the reaction mixture was allowed to warm to room temperature and stirred for 1 h. Subsequently, hexane (50 mL) was added, and the mixture was placed in a freezer to promote precipitation of triphenylphosphine oxide. The precipitate was removed by filtration, and the filtrate was concentrated under reduced pressure. This precipitation and filtration procedure was repeated several times (until no triphenylphosphine oxide was detectable by ^{31}P NMR) to ensure complete removal of the byproduct. The resulting product was obtained as a colorless liquid, which was dried under vacuum (5 mbar) overnight at room temperature and stored in a refrigerator.

Oleyl bromide. Yield: 98%. Appearance: colorless liquid. ^{1}H NMR (300 MHz, $CDCl_3$) δ, ppm: 5.36-5.32 (m, 2H), 3.41 (t, *J* = 7.5 Hz, 2H), 2.01-1.99 (m, 4H), 1.87-1.82 (m, 2H), 1.44-1.26 (m, 22H), 0.87 (t, *J* = 6.6 Hz, 3H). Mass spectrum (m/z): $[M+H]^+$ calcd for $C_{18}H_{36}Br$, 331.2000; found 331.1995.

Step 2. Preparation of Grignard reagent Oleyl MgBr. Oleyl MgBr was synthesized following the procedure described elsewhere.[4]

An oven-dried Schlenk flask was charged with magnesium turnings (52.5 mmol, 2.15 equiv.), anhydrous THF (50 mL), oleyl bromide (25 mmol, 1.00 equiv.), and a few drops of dry 1,2-dibromoethane. The mixture was stirred at room temperature overnight. The resulting solution was then transferred to the subsequent reaction step via cannula transfer.

Step 3. The synthesis of DOlPA was performed according to the general procedure for the preparation of dialkyl phosphinic acids described above, using the Grignard reagent obtained in Step 2.

Bis[(Z)-octadec-9-enyl]phosphinic acid **(dioleylphosphinic acid, DOlPA)**. The residue was purified by column chromatography using hexane/EtOAc (2:1, v/v), followed by elution of the product with EtOAc. Yield: 38%. Appearance: colorless liquid. ^{1}H NMR (300 MHz, $CDCl_3$) δ, ppm: 9.32 (s, 1H), 5.43 – 5.26 (m, 4H), 2.07 – 1.90 (m, 8H), 1.74 – 1.49 (m, 8H), 1.46 – 1.16 (m, 44H), 0.96 – 0.83 (m, 6H). ^{31}P NMR (121 MHz, $CDCl_3$) δ, ppm: 60.40. Mass spectrum (m/z): $[M+H]^+$ calcd for $C_{36}H_{72}O_2P$, 567.5264; found 567.5257

2.3. Synthesis of didodecyl hydrogen phosphate ($DC_{12}Pate$)

Synthesis was performed as described elsewhere.[5]

A solution of $POCl_3$ (0.017 mol, 1 equiv.) in anhydrous toluene (20 mL) was stirred at room temperature while a mixture of *n*-dodecanol (0.03 mol, 1.8 equiv.) and Et_3N (0.03 mol, 1.8 equiv.) was added slowly, ensuring that the reaction temperature did not exceed 60°C. Upon completion of the addition, the mixture was filtered to remove the $Et_3N{\cdot}HCl$ byproduct. The filtrate, containing didodecyl chlorophosphate dissolved in toluene, was then stirred vigorously while steam was bubbled through the solution for 1h. The organic layer was separated, dried over Na_2SO_4, filtered, and the solvent was removed under reduced pressure to yield pure didodecylphosphoric acid as white needle-like crystals. The resulting product was dried overnight under a vacuum (5 mbar) at 50 °C.

Didodecyl hydrogen phosphate (**$DC_{12}Pate$**). Yield: 70%. Appearance: white crystals. ^{1}H NMR (300 MHz, $CDCl_3$) δ, ppm (J, Hz): 10.42 (s, 1H), 4.01 (q, *J* = 6.7 Hz, 4H), 1.74 – 1.61 (m, 4H), 1.42 – 1.20 (m, 36H), 0.93 – 0.83 (m, 6H). ^{31}P NMR (121 MHz, $CDCl_3$) δ, ppm: 1.2. Mass spectrum (m/z): $[M+Na]^+$ calcd for $C_{24}H_{51}NaO_4P$, 457.3417; found 457.3420

3. Synthesis of lead bromide perovskite QDs

3.1. Synthesis of C_8C_{12}-PEA ligand

The 2-ammonioethyl 2-octyl-1-dodecyl phosphate (C_8C_{12}-PEA) ligand was synthesized according to the procedure described in earlier report.[6]

Solution of 2-octyl-1-dodecanol (0.025 mol; 1 equiv) dissolved in dry tetrahydrofuran (THF) (25 mL) along with triethylamine (0.275 mol; 1.1 equiv; 3.83 mL) was added dropwise with vigorous stirring to a solution of phosphorous oxychloride (0.03 mol; 1.2 equiv; 2.78 mL) in THF (2.5 mL) previously cooled down with an ice water bath. The reaction mixture was subsequently kept at 20 °C for 15 min to complete the reaction. Next, ethanolamine (0.03 mol; 1.2 equiv; 1.81 mL) and triethylamine (0.06 mol; 2.4 equiv) in THF (37.5 mL) were added dropwise under vigorous stirring to the reaction mixture kept in a room-temperature water bath. Subsequently, the mixture was heated to 40 °C for 15 min to complete the ring closure. Finally, the reaction mixture was filtered to remove precipitated triethylamine hydrochloride, and the filtrate solution was dried. An oily residue of alkyl-2-oxo-1,2,3-oxazaphospholane was dissolved in a mixture of acetic acid (5.7 mL) and distilled water (2.6 mL) at 70 °C. After 30 min, ring scission at the P–N bond is complete, and the product is separated by shaking with acetone (125–150 mL). After cooling to 10 °C, 2-ammonioethyl 2-octyl-1-dodecyl phosphate is collected and dried overnight under a vacuum at 40–50 °C. NMR spectrum of the product is in agreement with the literature.[6]

3.2. Synthesis of OGB ligand

N-(octadec-9-en-1-yl) guanidinium bromide (OGB) ligand was synthesized according to the procedure described in earlier report.[7]

Respective S-ethylisothiourea hydrobromide (1.2 eq) was suspended in THF (0.1 g/mL), and oleylamine (1 eq) was added. The resulting mixture was stirred at room temperature overnight. Then, THF was evaporated under reduced pressure, and the resulting residue was recrystallized with Et_2O. The precipitate obtained was washed a couple of times with Et_2O and dried under reduced pressure overnight at 50°C. NMR spectrum of OGB is in agreement with the literature.[7]

3.3. Synthesis of $CsPbBr_3$ QDs

$CsPbBr_3$ QDs capped with zwitterionic ligands were synthesized according to the modified procedure described earlier.[8]

$PbBr_2$ precursor solution (0.04 M) was prepared from $PbBr_2$ (1 mmol, 367 mg) and TOPO (90%) (5 mmol, 1.93 g) dissolved in n-octane (5 ml) at 110 °C on a hotplate in the air. Once all the $PbBr_2$ was dissolved, the vial was cooled to room temperature, and 20 mL of hexane was added. The resulting solution was filtered through a 0.22 µm PTFE filter and stored under ambient conditions.

Cs precursor solution (0.02 M) was prepared by loading Cs_2CO_3 (100 mg, 0.3069 mmol) together with the acid of choice (3.154 mmol) and 2 mL of octane at 110°C in a vial. Once all the Cs_2CO_3 was dissolved by reacting with the corresponding acid, the stock solution was cooled to room temperature, and 27 mL of octane was added. The resulting solution was filtered through a 0.22 µm PTFE filter and stored under ambient conditions. In some cases, precipitation of the precursor was observed upon cooling; such solutions were reheated before injection to ensure complete redissolution.

Cs precursors using acids with incorporation of aromatic groups into the tail (DPhPA/DPhEtPA/DBnPate) were prepared in mesitylene instead of octane.

Lecithin ligand solution (0.13 M) was prepared by dissolving 0.5 g of lecithin in 10 mL of hexane. The solution was sonicated until the complete dissolution of lecithin, filtered through a 0.22 μm PTFE filter, and stored under ambient conditions.

C_8C_{12}-PEA ligand solution (0.1 mg/μL) was prepared by dissolving 100 mg of C_8C_{12}-PEA ligand in 1 mL of mesitylene. The solution was stirred until the compound had completely dissolved.

OGB ligand solution (1.3 M) was prepared by dissolving 252.5 mg of OGB ligand in 0.5 mL of toluene. The solution was stirred on a hotplate until the compound had completely dissolved.

For the synthesis of $CsPbBr_3$ QDs, Cs precursor was swiftly injected into the mixture of hexane and $PbBr_2$ precursor under vigorous stirring at room temperature and left for a defined amount of time to nucleate and grow QDs. The synthetic details are summarized in the table below and in the Table S1.

dilution	n-hexane	$PbBr_2$ precursor	Cs precursor	reaction time	ligand exchange
3.3 mM	3 mL	2 mL	1 mL	10 min	1 mL lecithin
1.33 mM	12 mL	2 mL	1 mL	15 min	1 mL lecithin
0.71 mM	25 mL	2 mL	1 mL	30 min	1 mL lecithin
0.377 mM	50 mL	2 mL	1 mL	60 min	1 mL lecithin
0.15 mM	100 mL	2 mL	1 mL	5 min	100 μL br-PEA

For Cs precursors bearing aromatic groups (Cs-DPhPA/Cs-DPhEtPA/Cs-DBnPate), the synthesis was conducted in mesitylene instead of hexane.

Upon growth completion, the ligand solution was swiftly injected, and the crude mixture was stirred for another 1 minute to ensure ligand exchange.

Purification. QDs synthesized at dilutions of 3.3-0.71 mM were purified without prior concentration. For synthesis at higher dilutions (0.377 mM and 0.15 mM), the crude solution was first concentrated to ~5 mL under reduced pressure.

To purify QDs *capped with lecithin*, acetone was added to the crude solution in a 2:1 ratio, followed by centrifugation for 2 min at 12.1 krpm (20133 × g). The precipitate was redispersed in 1 mL of cyclohexane. The resulting colloidal solution was centrifuged for 2 min at 12.1 krpm (20133 × g) and used for further investigations.

To purify QDs *capped with C_8C_{12}-PEA ligand*, EtOAc/MeCN antisolvent mixture (2:1) was added to the crude solution in a 2:1 ratio, and the resulting solution was centrifuged for 2 min at 12.1 krpm (20133 × g). The precipitate was redispersed in 1 mL of cyclohexane. The resulting colloidal solution was centrifuged for 2 min at 12.1 krpm (20133 × g) and used for further investigations.

3.4. Synthesis of $CsPbBr_3$ QDs capped with oleylguanidinium bromide (OGB)

To verify that, beyond serving as Cs precursors, dialkylphosphinates also act as weakly bound QD surface ligands, $CsPbBr_3$ QDs were capped with a small amount (one-fifth of that used in the reported procedure, ref[7]) of the dynamic guanidinium-based OGB ligand (Figure S8). This amount is insufficient to fully cover the QD surface, yet sufficient to enable purification of the QDs from the crude solution. Such purification removes unreacted precursors and by-products, leaving only surface-bound species, which can then be probed by ^{31}P NMR.

This experiment was performed on $CsPbBr_3$ QDs synthesized at 1.33 M dilution in the presence of Cs-$DC_{12}PA$ precursor. Upon growth completion, 20 µL of OGB solution (1.3 M) was swiftly injected, and the crude mixture was stirred for another 1 minute to ensure ligand exchange.

__Purification__. The crude solution was concentrated to ~4 mL under reduced pressure. Subsequently, 4.5 mL of an EtOAc/MeCN antisolvent mixture (2:1) was added, and the resulting solution was centrifuged for 2 min at 12.1 krpm (20133 × g). The precipitate was redispersed in 0.5 mL of C_6D_6. To the resulting colloidal solution, 2.5 mL of EtOAc/MeCN mixture was added, followed by centrifugation for 2 min at 12.1 krpm (20133 × g). The precipitate was redispersed in 0.5 mL of C_6D_6. The final solution was centrifuged for 1 min at 12.1 krpm (20133 × g), and 0.5 mL of the resulting colloidal solution was directly used for further ^{31}P NMR investigations.

3.5. Synthesis of $FAPbBr_3$ QDs

$FAPbBr_3$ QDs capped with C_8C_{12}-PEA ligand were synthesized according to the modified procedure described earlier.[6]

__$PbBr_2$ precursor and C_8C_{12}-PEA ligand solutions__ were prepared as described above for the synthesis of $CsPbBr_3$ QDs.

__FA-DOPA/OAc precursor solution__ (0.061 M) was prepared from FAOAc (64 mg, 0.61 mmol), DOPA (3 mL, 9.462 mmol), and OAc (2 mL, 6.34 mmol) dissolved in n-octane (5 mL) at 110°C on a hotplate under ambient conditions until fully dissolved. The resulting solution was filtered through a 0.22 µm PTFE filter and stored under ambient conditions.

__All other FA-DRPA and FA-DRPate precursor solutions__ (0.061 M) were prepared by dissolving FAOAc (64 mg, 0.61 mmol) together with the acid of choice (6.265 mmol) in *n*-octane (5 mL) at 110 °C on a hotplate under ambient conditions until fully dissolved. While still warm, the solution was diluted with *n*-octane to a final volume of 10 mL. In some cases, precipitation of the precursor was observed upon cooling; such solutions were reheated prior to injection to ensure complete redissolution.

__For the synthesis of $FAPbBr_3$ QDs__, FA precursor was swiftly injected into the mixture of hexane and $PbBr_2$ precursor under vigorous stirring at room temperature and left for 10 seconds to nucleate and grow QDs. After that, C_8C_{12}-PEA ligand solution was added, and the reaction mixture was stirred for another 1 minute to ensure ligand exchange (see Table S2 for details).

__Purification__. The crude solution was concentrated to ~3 mL under reduced pressure. Subsequently, 6 mL of an EtOAc/MeCN antisolvent mixture (2:1) was added, and the resulting solution was centrifuged for 2 min at 12.1 krpm (20133 × g). The precipitate was redispersed in 1 mL of cyclohexane. To the resulting colloidal solution, 5 mL of EtOAc/MeCN mixture was added, followed by centrifugation for 2 min at 12.1 krpm (20133 × g). The precipitate was redispersed in 0.5 mL of cyclohexane. The final colloidal solution was centrifuged for 3 min at 12.1 krpm (20133 × g) and used for further investigations.

4. Characterization methods

4.1. UV-Vis optical absorption spectroscopy

UV-Vis absorption spectra for colloidal solutions were collected using a Jasco V670 spectrometer in transmission mode. Samples were diluted in hexane. The 1st excitonic transition was determined by taking the minimum of the 2nd derivative of the absorption spectrum.

4.2. Photoluminescence (PL) and absolute PL Quantum Yield (QY) measurements

A Fluorolog iHR 320 Horiba Jobin Yvon spectrofluorometer equipped with a PMT detector was used to acquire steady-state PL spectra from solutions. Samples were excited at 385 nm. QD solutions were measured using the same dilutions and solvents as in the absorption measurements.

The QY of the samples was measured in Hamamatsu Quantaurus-QY Plus UV-NIR absolute PL spectrometer (C13534-11) equipped with an integrating sphere. The films for QY measurements were prepared by drop-casting 10 µl of the sample mixed with 50 µl of the cyclohexane on the square 10mm x 10mm cleaned glass substrates.

4.3. Electron Microscopy

Transmission electron microscopy (TEM) and scanning transmission electron microscopy (STEM) images were captured using a JEOL JEM-F200 microscope operated at 200 kV. Samples were prepared by drop casting diluted colloidal solution of NCs (~1 mg/mL) on 200 mesh copper grid coated with Formvar/Carbon. The sizes of representative QDs were measured for monodisperse samples. Full size distributions were determined only for polydisperse $CsPbBr_3$ QD samples and for $FAPbBr_3$ QDs.

4.4. *In situ* Absorption spectroscopy

In situ absorption spectroscopy studies were performed on homemade reactor with solution pumped through a 1 cm quartz (UV transparent) cuvette and spectra collected with a wide-band spectrometer (Ocean HDX-UV-VIS) and a deuterium light source (Thorlabs SLS204). Integration time was set to 30 ms and every 10 spectra were averaged to get a 300 ms time resolution. Custom-developed Python scripts were employed for the batch data analysis and fitting.

4.4.1. QD size and concentration calculation

The time evolution of $CsPbBr_3$ QD size was derived from the 1st excitonic transition energy, determined as the minimum of the 2nd derivative of the absorption spectrum. The extracted transition energies were converted to QD sizes using the sizing curve reported by Akkerman *et al.*[8]

$$d = a \cdot \left(E_{\mathrm{QD}} - E_{\mathrm{bulk}}\right)^{c}$$

with the following fitting parameters: $a = 1.90$, $E_{\mathrm{bulk}} = 2.38$ and $c = -0.588$.

The concentration of $CsPbBr_3$ was estimated from the solution absorbance within the size-independent wavelength range. Absorbance at 400 nm and 335 nm is commonly used for this purpose, as corresponding extinction coefficients are available in the literature.[9] However, in the present case, QD growth proceeds from very small sizes (< 4 nm), for which absorption at 400 nm remains affected by quantum confinement. Absorption at 335 nm could also not be used, as it is influenced by residual precursor species in the crude solution and by detector saturation. Therefore, absorbance at 375 nm was selected, as it is unaffected by both

limitations. The intrinsic absorption coefficient at this wavelength was estimated as $\mu_{375\text{ nm}} = 2.67\cdot10^4$ cm^{-1} in *n*-hexane based on the data reported in Ref.[9] The molar concentration of $CsPbBr_3$ (C_{mat}) was then estimated using the following equation:

$$C_{\text{mat}} = \frac{A_\lambda\, \rho \ln 10}{\mu_\lambda l M}$$

Where A_λ is the absorbance at 375 nm, μ_λ the intrinsic absorption coefficient at the same wavelength, l the optical path (1 cm), ρ the density (4.73 g.cm^3) and M the molar mass (579.3 g.mol^{-1}) of $CsPbBr_3$.

The concentration of QD produced C_{QD} is:

$$C_{QD} = \frac{A_\lambda\ \ln 10}{\mu_\lambda l N_a V_{QD}}$$

where V_{NC} is the volume of a single QD and N_a is the Avogadro constant. We assume a spherical shape of growing QDs following Ref.[10]

4.5. Mass Spectrometry (MS)

MS was conducted at the Molecular and Biomolecular Analysis Service (MoBiAS) of ETH Zurich.

4.6. Nuclear Magnetic Resonance Spectroscopy (NMR)

Solution-state ^{1}H and ^{31}P NMR spectra for acid characterization were recorded in deuterated solvents at room temperature on a Bruker Avance III spectrometer operating at a proton frequency of 300.13 MHz and equipped with a 5 mm BBO probe. Chemical shifts (δ) are reported in ppm and referenced to the residual ^{2}H signal of the deuterated solvent. ^{1}H spectra were acquired using a 30° excitation pulse and a recycle delay of 5 s. For ^{31}P NMR measurements, a recycle delay of 1.2 s was used while maintaining a 30° excitation pulse.

Diffusion Ordered Spectroscopy: ^{31}P DOSY experiments were conducted on an AVANCE III HD (Bruker) spectrometer at 11.7 T (500 MHz) using a 5 mm PABBO probe with 50 G/cm maximum Z-gradient. The recycle delay was set to 1.3 times T_1, as previously determined from a saturation recovery experiment. Diffusion measurements were carried out using a double-stimulated echo sequence with two spoil gradients as proposed by Jerschow and Mueller.[11, 12] The length of the gradient pulses δ is kept constant at 2.5 ms while the amplitude is varied for a diffusion time Δ of 200 ms. The diffusion characteristics were then determined by fitting the signal attenuation as a function of the gradient strength using the Stejskal-Tanner equation.[13]

$$I = e^{-D_{measured}}\gamma^2\delta^2 g^2\Delta'$$

With D being the diffusion coefficient, γ being the gyromagnetic ratio and g being the gradient strength. The only fitted parameter is the diffusion coefficient, as all other variables are either experimental parameters or physical constants.

4.7. Small-angle X-ray scattering (SAXS) and wide-angle X-ray scattering (WAXS)

SAXS and WAXS patterns of colloidal $FAPbBr_3$ QDs in cyclohexane were collected using a XENOCS Xeuss 3.0 instrument equipped with a Cu-Kα source (1.54 Å) and a Pilatus1M detector. Samples were exposed for 30 min (SAXS) and 10 min (WAXS). The sample-to-detector distances were set to 300 mm for SAXS and 50 mm for WAXS, calibrated using silver behenate. Samples were loaded into 1 mm borosilicate capillaries. The data

processing steps, including masking, azimuthal integration, and background subtraction, were performed using the instrument software.

4.7.1. Size determination by SAXS

The size of $FAPbBr_3$ QDs was determined following the procedure described by Maes *et al.*[14] The sample concentration was optimized to minimize interparticle interactions and maximize scattering intensity.

A cubic shape of QDs was assumed, as indicated by TEM images. Accordingly, the data were fitted using a cubic form factor with a Gaussian size distribution, implemented in the SasView software. The scattering length densities of $FAPbBr_3$ and cyclohexane are $29.2 \cdot 10^6$ Å^{-2} and $7.55 \cdot 10^6$ Å^{-2}, respectively. The fit results are summarized in Table S3.

For a Gaussian size distribution with mean value $\bar{x}$ and standard deviation σ, the polydispersity PD is defined as $PD = \sigma/\bar{x}$.

4.7.2. Sizing curve

The sizes determined by SAXS and TEM were related to the 1st excitonic absorption transition determined from the minimum of the second derivatives of the UV-Vis absorption spectra. Available literature data was included in the TEM dataset. Sizes obtained by SAXS and TEM were fit independently to the semi-empirical function proposed by Aubert *et al.*[15] with a dielectric constant fixed to either 8.6 (from magneto-optical experiments),[16] or 6.0 (from ellipsometry).[17] In both cases fitting is nearly identical (Figure S27). The fitting parameters are reported in Table S4. Reduced effective mass μ was calculated from the fitted Bohr radius to be compared with the literature data. Fitted bandgap, Bohr radius, and reduced effective mass are in good agreement with literature: 2.30 eV,[17] 3.9 nm,[18] and $0.13m_0$ respectively.[16]

5. Supporting figures

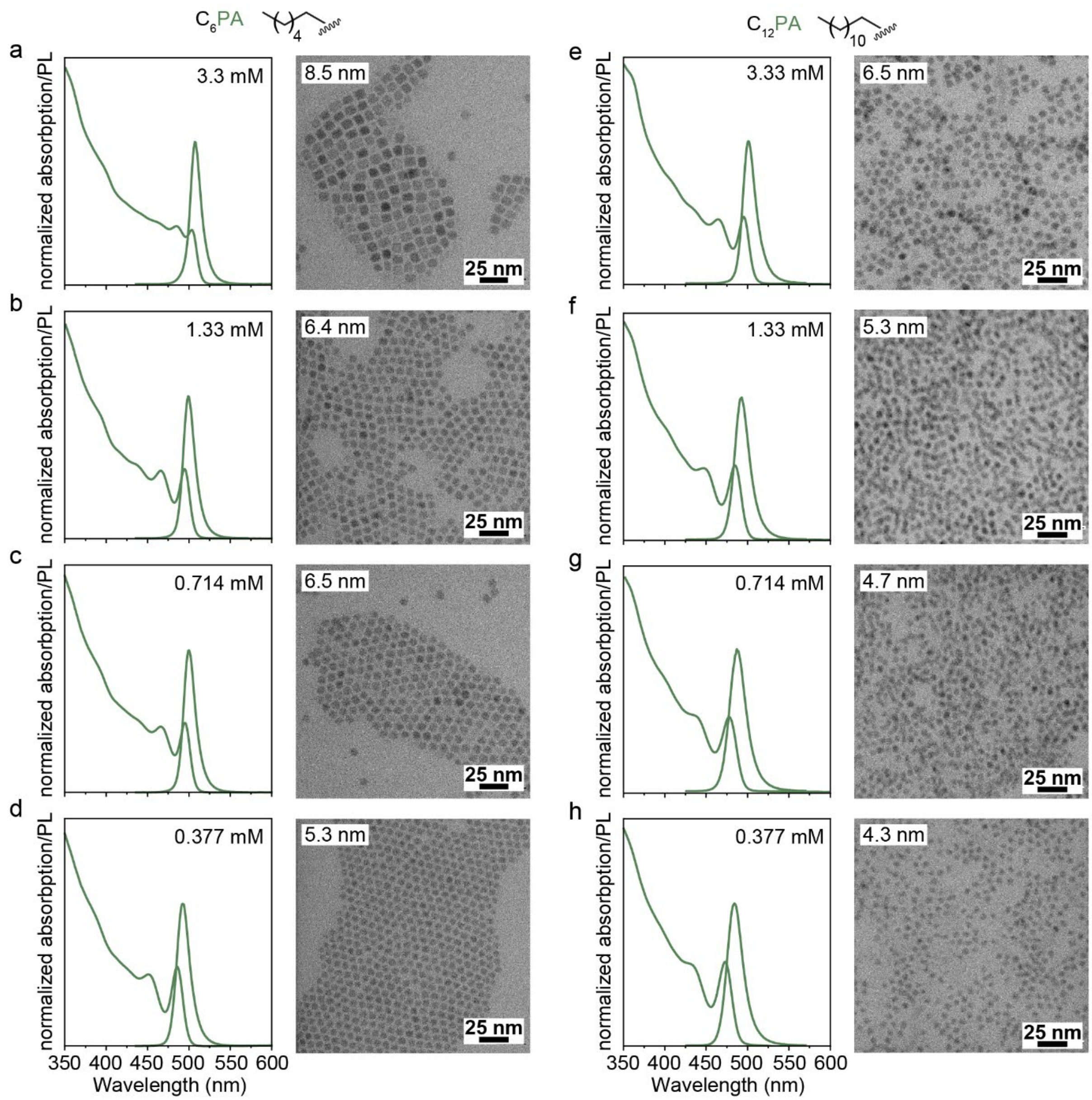

Figure S1. The representative normalized absorption and photoluminescence (PL) spectra along with the corresponding bright-field STEM images of $CsPbBr_3$ QDs synthesized at different dilutions (3.33 mM/1.33 mM/0.714 mM/0.377 mM) using Cs monoalkylphosphinates with different alkyl tail length: **(a-d)** Cs n-hexylphosphinate (Cs-C_6PA), **(e-h)** Cs dodecylphosphinate (Cs-C_{12}PA).

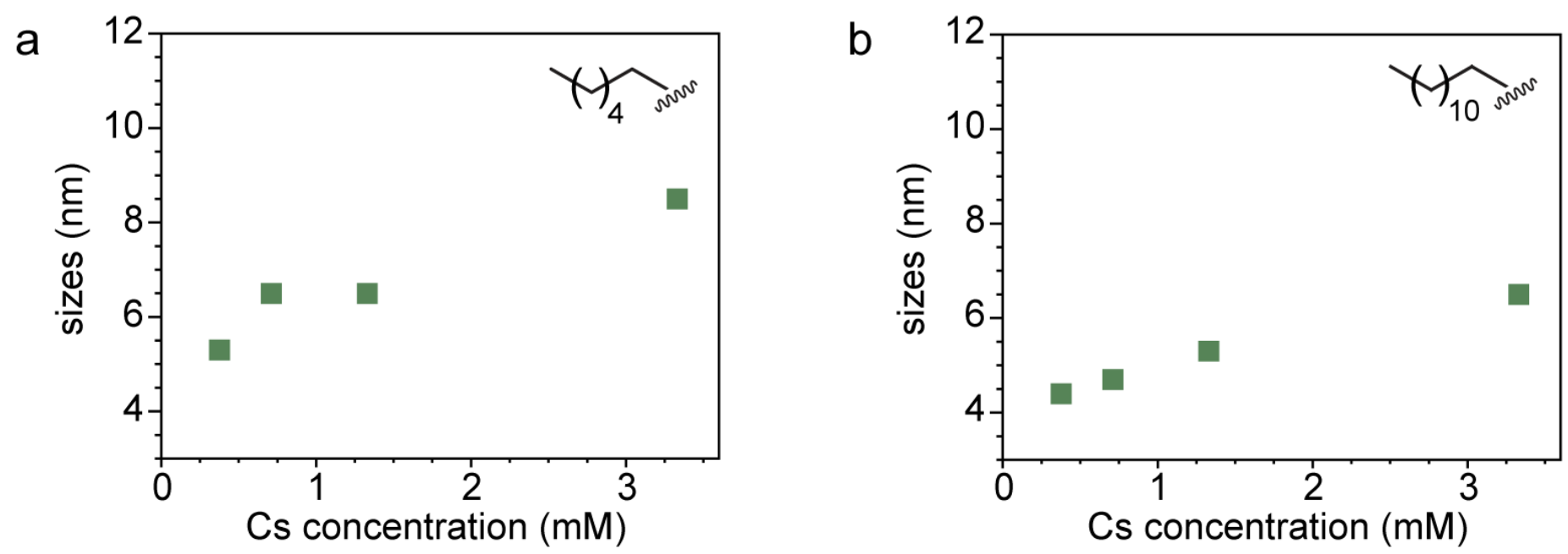

Figure S2. The size charts of $CsPbBr_3$ QDs synthesized at different dilutions using Cs monoalkylphosphinates Cs-RPA with different alkyl tail length: **(a)** Cs n-hexylphosphinate (Cs-C_6PA), **(b)** Cs dodecylphosphinate (Cs-C_{12}PA).

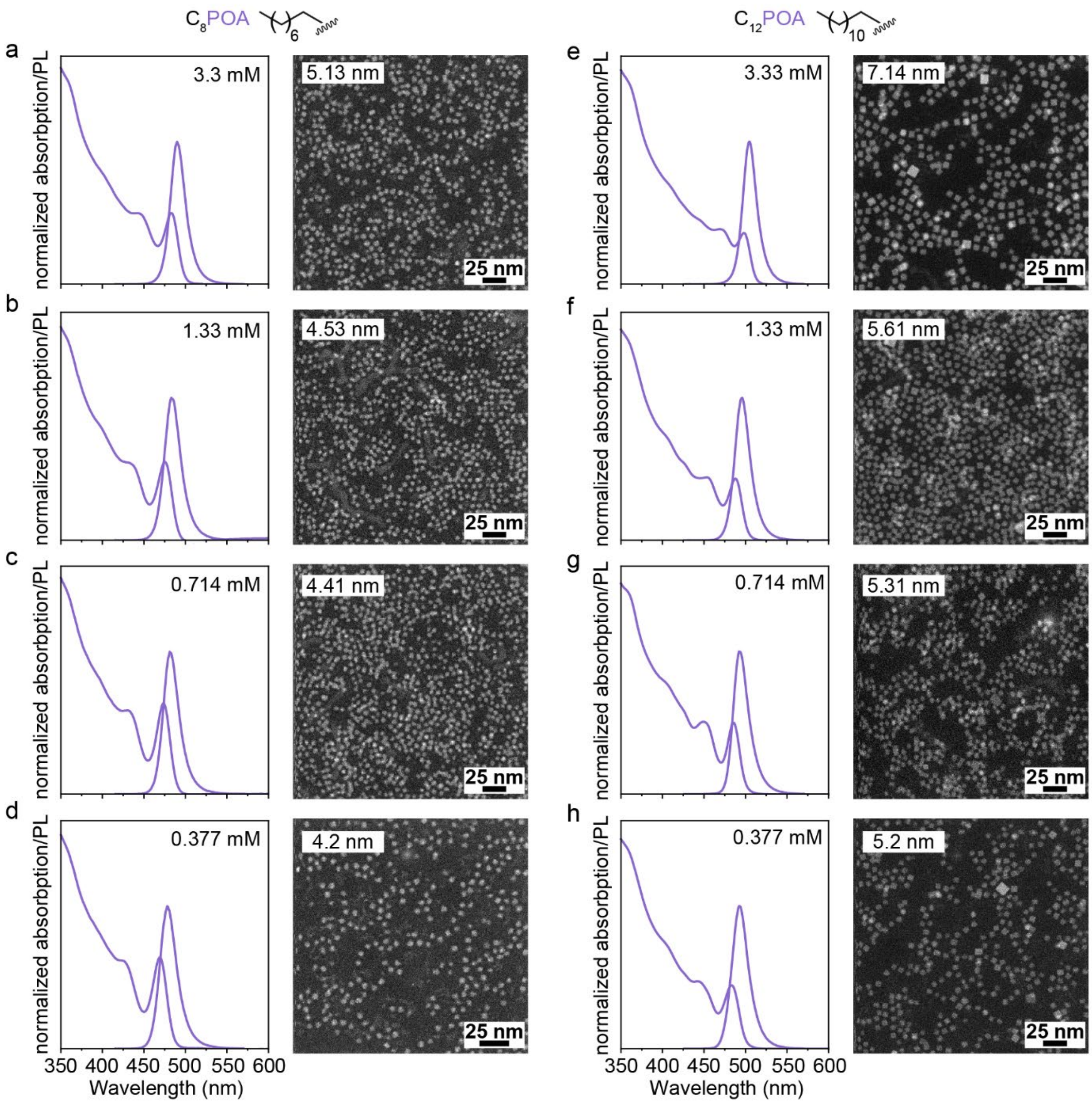

Figure S3. The representative normalized absorption and photoluminescence (PL) spectra along with the corresponding TEM images of $CsPbBr_3$ QDs synthesized at different dilutions (3.33 mM/1.33 mM/0.714 mM/0.377 mM) using Cs monoalkylphosphonates with different alkyl tail length: **(a-d)** Cs n-octylphosphonate (Cs-C_8POA), **(e-h)** Cs n-dodecylphosphonate (Cs-C_{12}POA) .

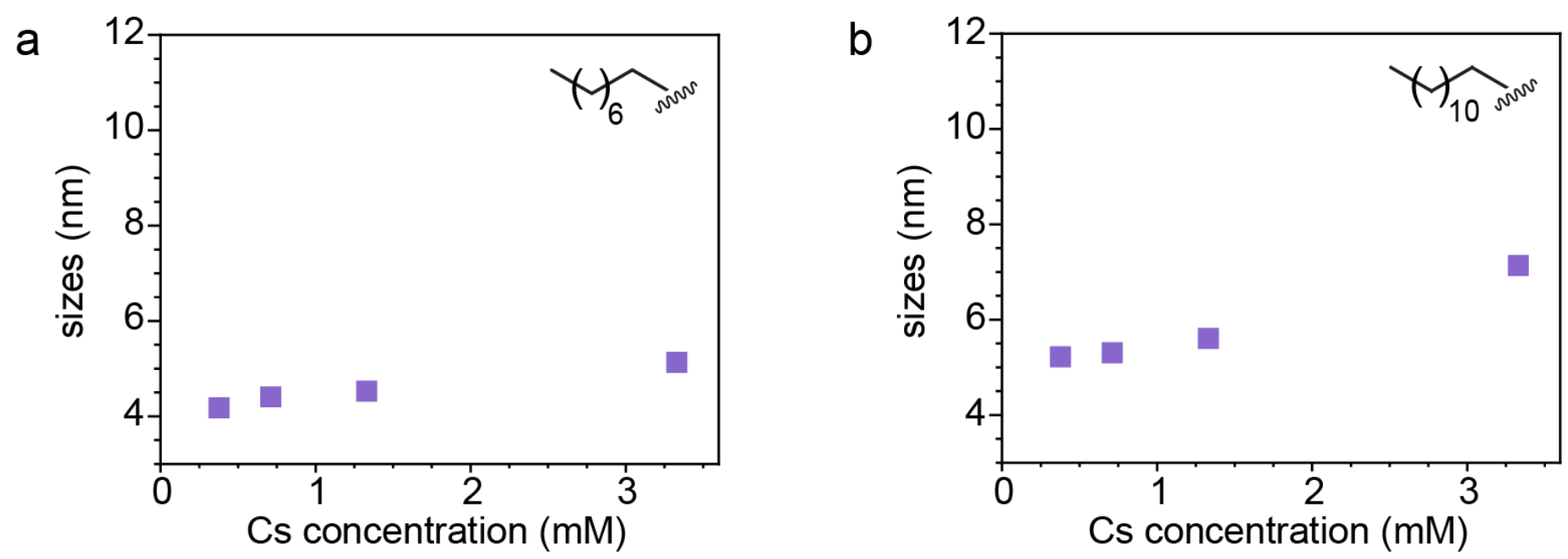

Figure S4. The size charts of $CsPbBr_3$ QDs synthesized at different dilutions using Cs monoalkylphosphonates Cs-RPOA with different alkyl tail length: **(a)** Cs n-octylphosphonate (Cs-C_8POA), **(b)** Cs n-dodecylphosphonate (Cs-C_{12}POA).

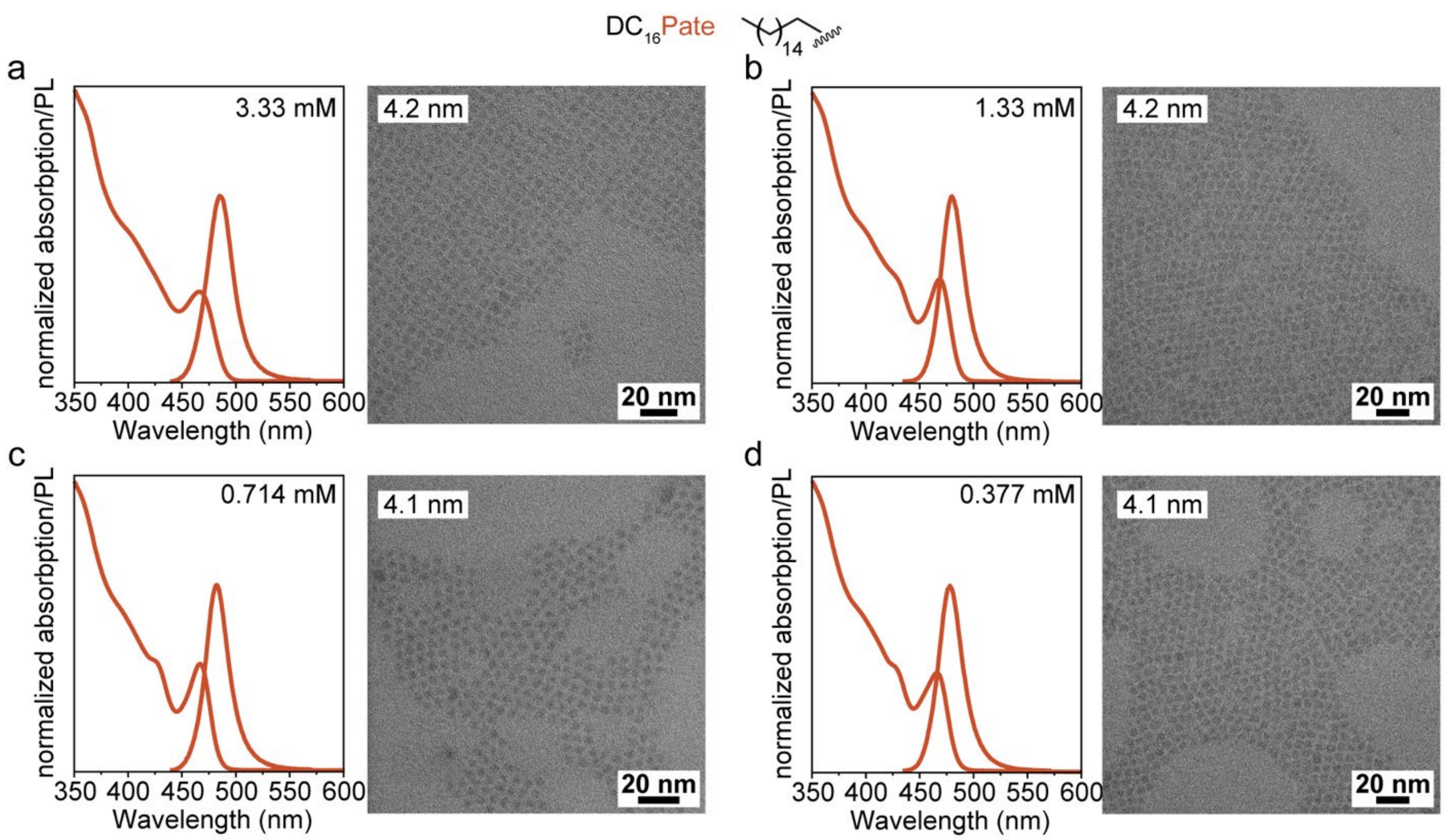

Figure S5. The representative normalized absorption and photoluminescence (PL) spectra along with the corresponding TEM images of $CsPbBr_3$ QDs synthesized using Cs dihexadecylphosphate (Cs-DC$_{16}$Pate) at different dilutions: **(a)** 3.33 mM, **(b)** 1.33 mM, **(c)** 0.714 mM, **(d)** 0.377 mM.

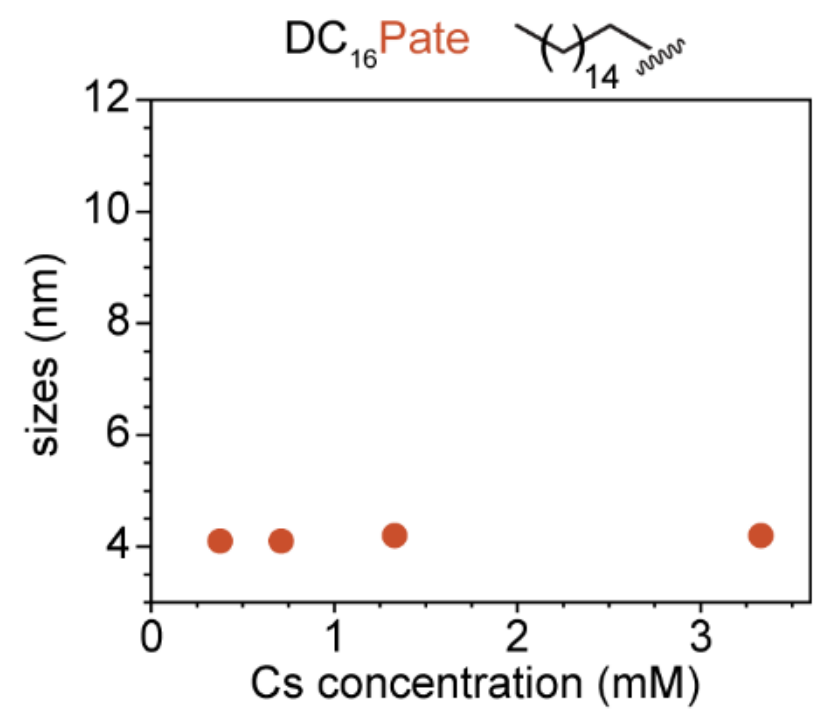

Figure S6. The size chart of $CsPbBr_3$ QDs synthesized at different dilutions using Cs-DC_{16}Pate.

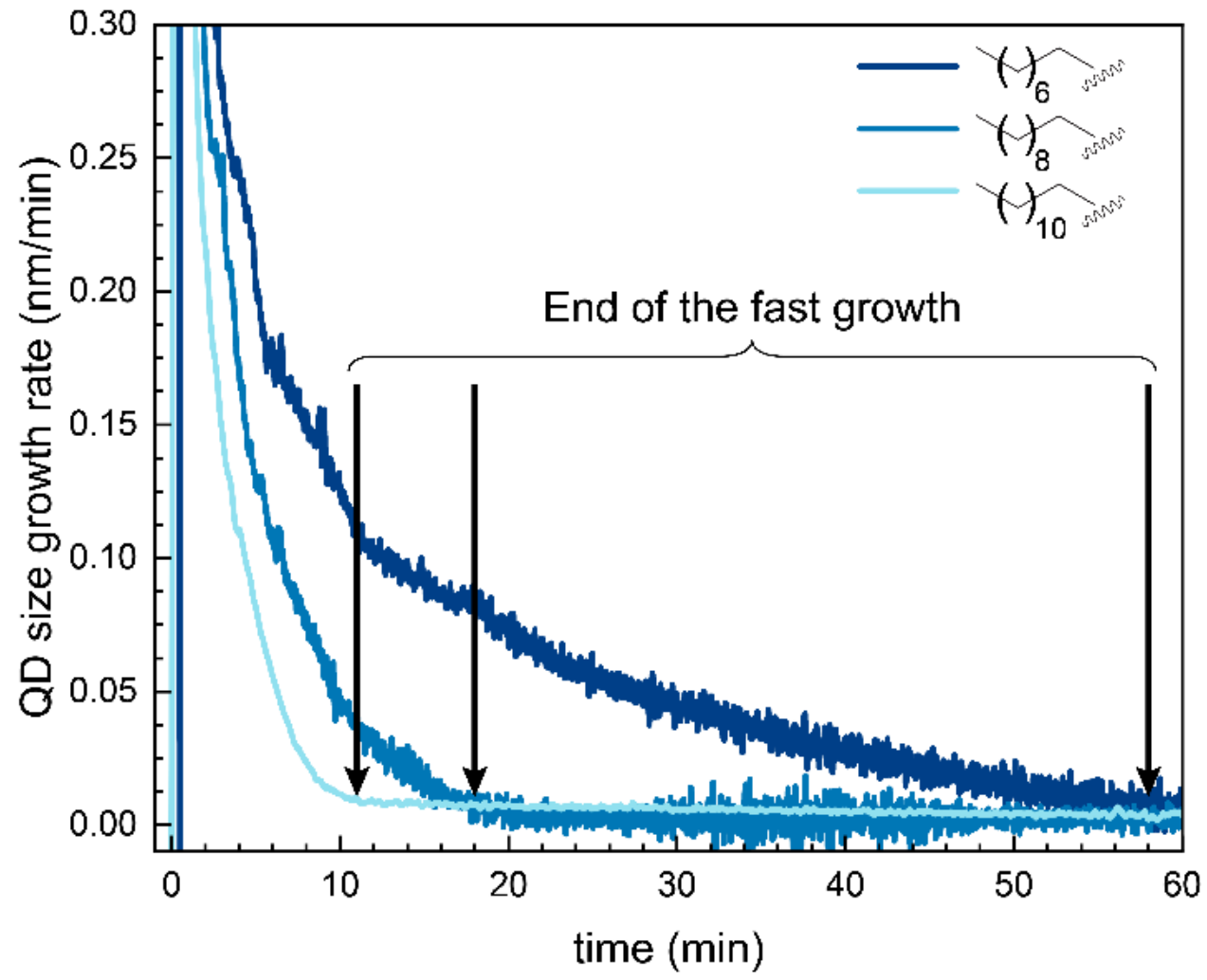

Figure S7. QD size growth rates, defined as the first derivative of size evolution for $CsPbBr_3$ QDs synthesized at 0.377 mM Cs concentration using Cs-DC_8PA, Cs-DC_8PA, and Cs-DC_8PA precursors. Vertical lines mark the end of fast growth and the system transition to ripening.

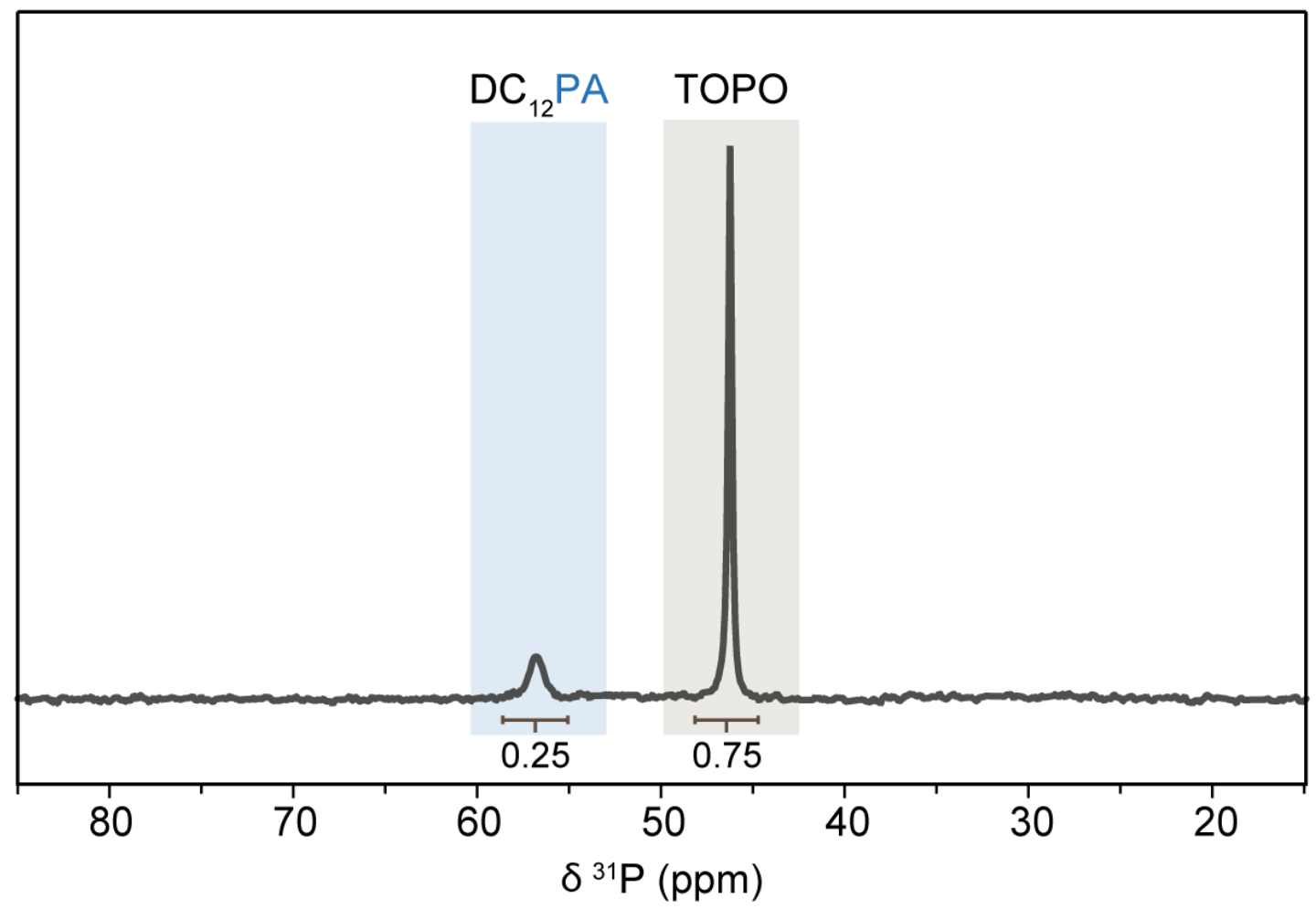

Figure S8. ^{31}P NMR of a colloidal solution of $CsPbBr_3$ QDs synthesized with Cs-DC_{12}PA precursor and capped with a minimum amount of OGB, which allowed purification of the synthesized QDs. After ligand exchange and two purification steps, residual signals corresponding to both TOPO (brown highlight) and DC_{12}PA (blue highlight) are still observed, indicating their incomplete substitution and persistence at the QD surface. The observed chemical shifts differ slightly from those of the free ligands in solution, suggesting surface binding. These species are therefore assigned to dynamically bound ligands in equilibrium with free molecules in solution, rather than residual unbound compounds remaining after purification.

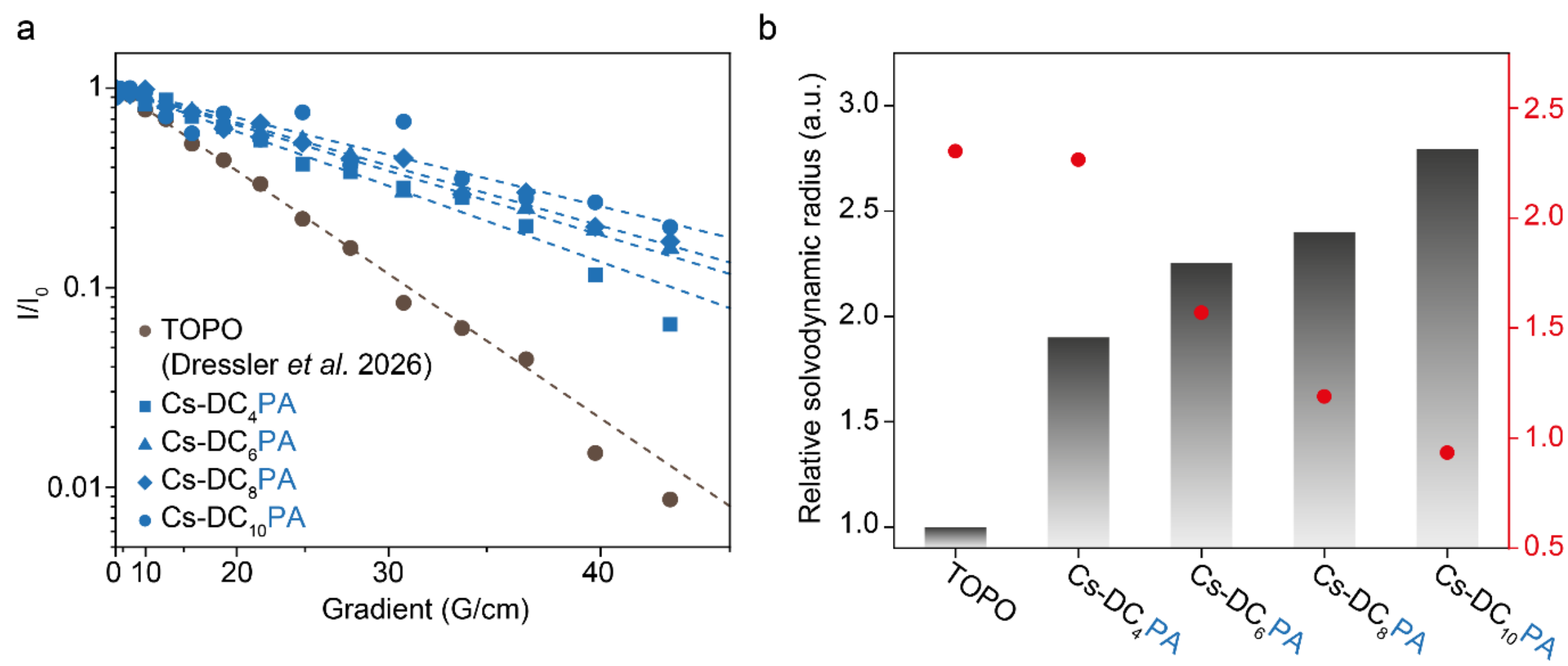

Figure S9. **(a)** Measured DOSY NMR traces for solutions of TOPO (brown) and Cs dialkylphosphinates with varying alkyl chain lengths (Cs-DC_4PA, Cs-DC_6PA, Cs-DC_8PA, Cs-DC_{10}PA; blue shades). All solutions were prepared at a synthetically relevant concentration of 3.3 mM. Dashed lines represent the corresponding fits. TOPO exhibits markedly faster signal attenuation, indicating a smaller solvodynamic radius compared to the cesium dialkylphosphinates. **(b)** Relative solvodynamic sizes estimated from the inverse relationship between the diffusion coefficient and particle size according to the Stokes–Einstein equation (grey bars), together with the corresponding ^{31}P T_1 relaxation times (red dots). The observed decrease in T_1 with increasing size is in qualitative agreement with Bloembergen–Purcell–Pound (BPP) theory.

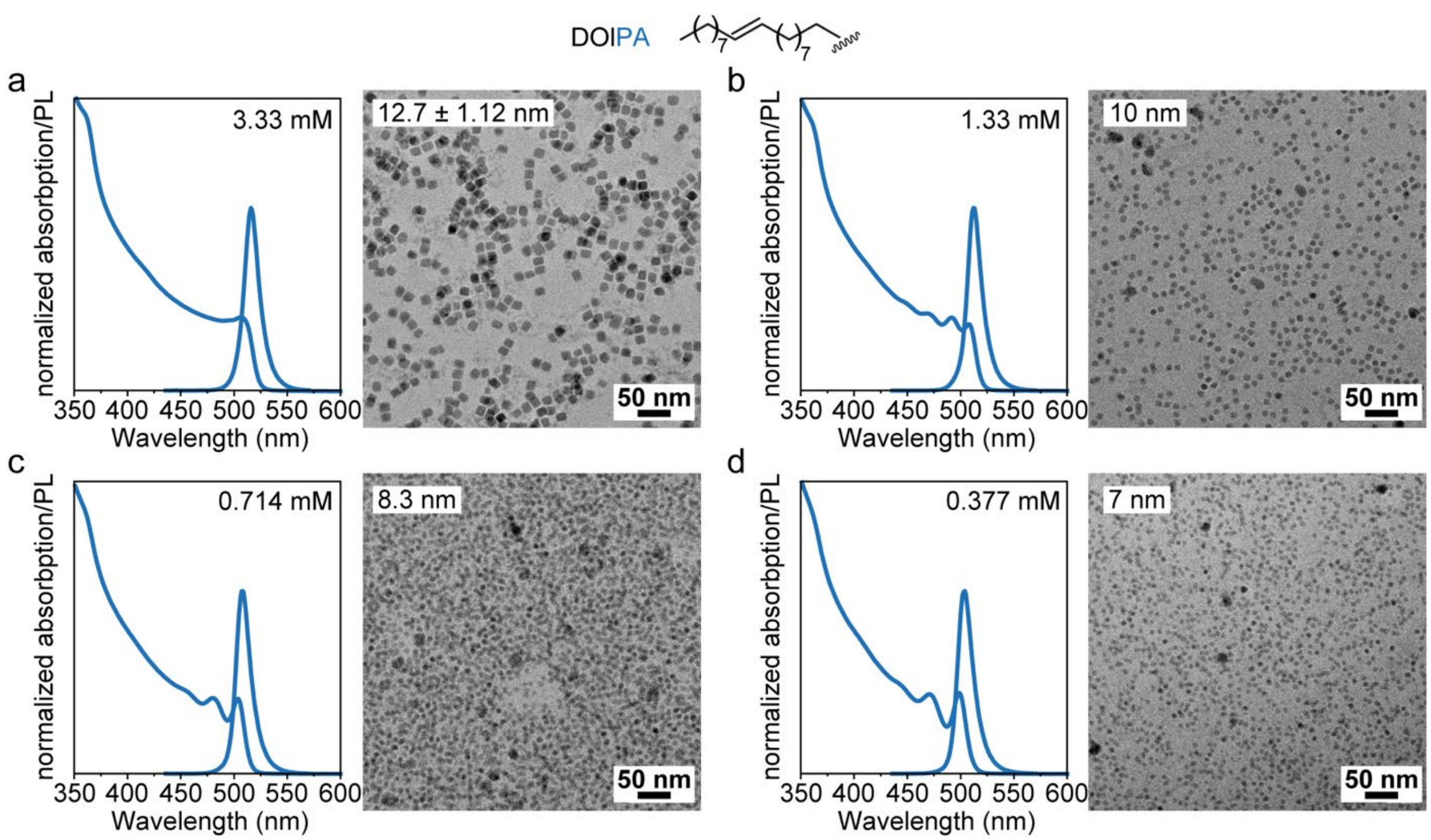

Figure S10. The representative normalized absorption and photoluminescence (PL) spectra along with the corresponding TEM images of $CsPbBr_3$ QDs synthesized using Cs dioleylphosphinic acid (Cs-DOlPA) at different dilutions: **(a)** 3.33 mM, **(b)** 1.33 mM, **(c)** 0.714 mM, **(d)** 0.377 mM.

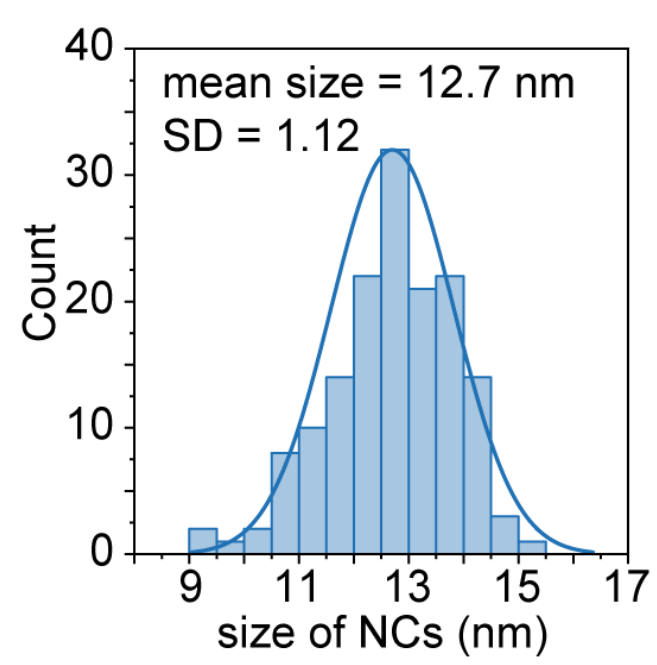

Figure S11. Size distribution histogram for $CsPbBr_3$ QDs synthesized using Cs dioleylphosphinic acid (Cs-DOlPA) at 3.3 mM dilution.

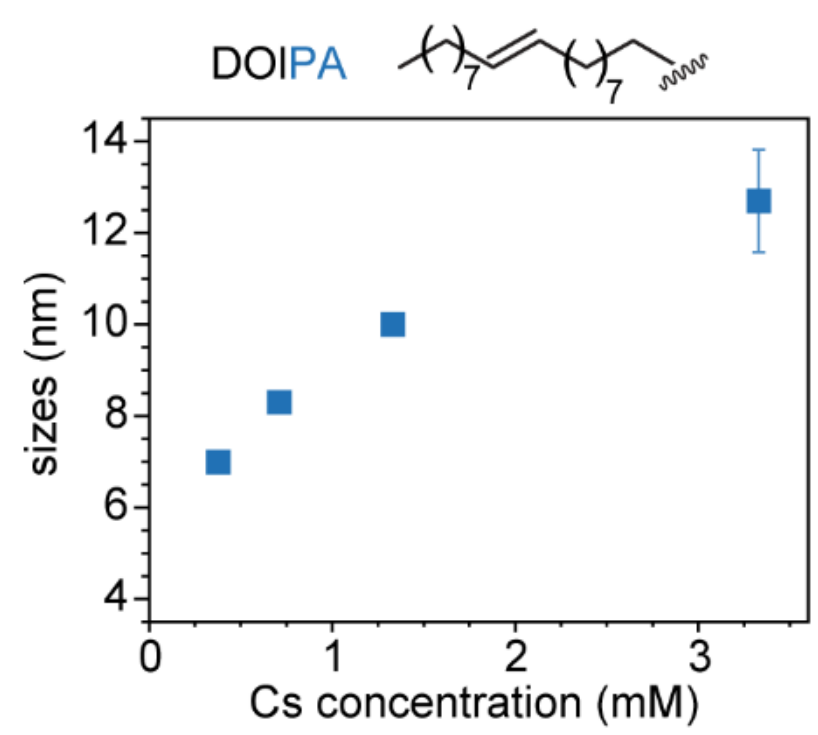

Figure S12. The size charts of $CsPbBr_3$ QDs synthesized at different dilutions using Cs-DOlPA.

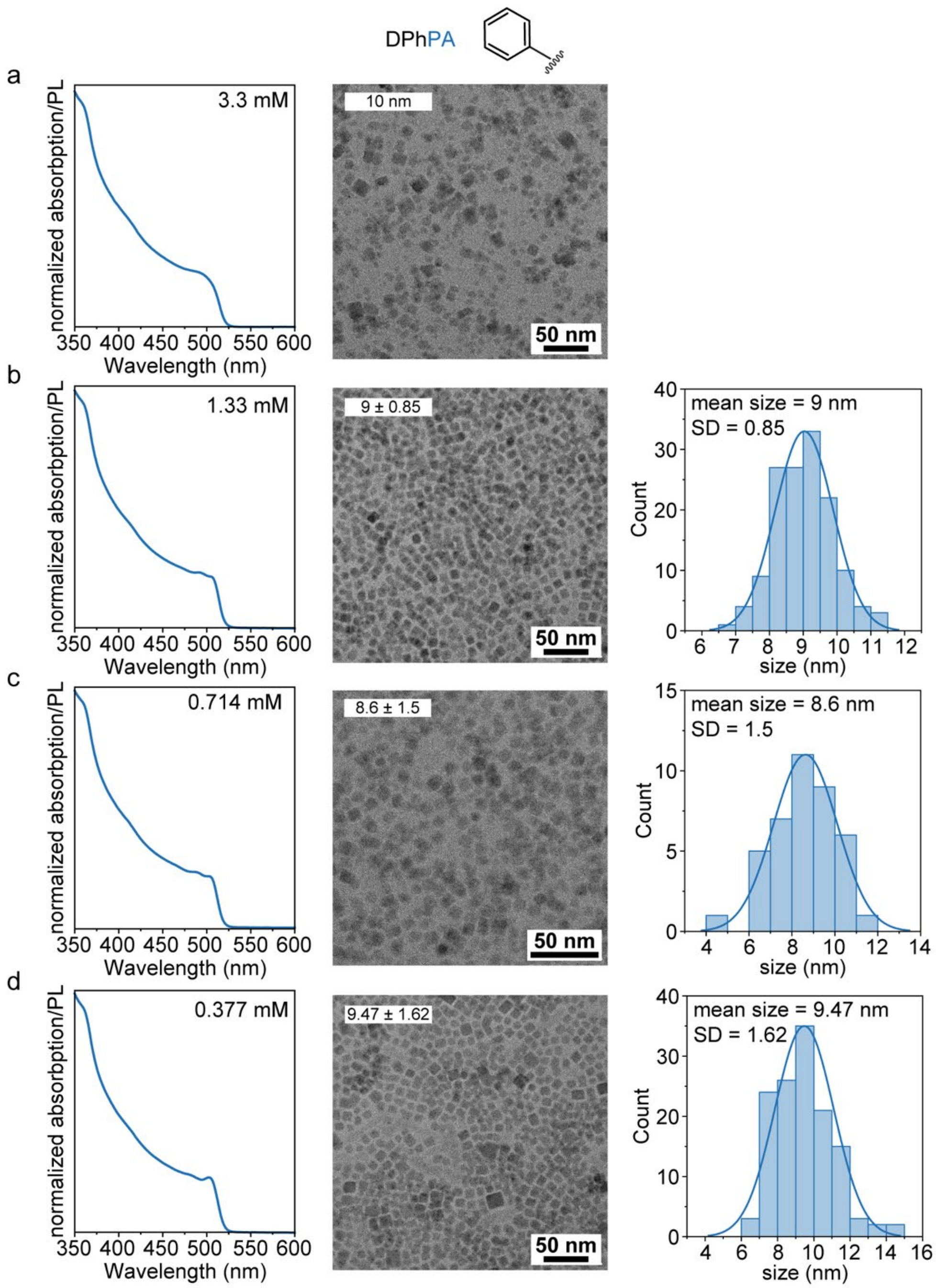

Figure S13. The representative normalized absorption and photoluminescence (PL) spectra of $CsPbBr_3$ QDs synthesized using Cs diphenylphosphinic acid (Cs-DPhPA) at different **(a)** 3.33 mM, **(b)** 1.33 mM, **(c)** 0.714 mM, **(d)** 0.377 mM dilutions (left panel) alongside corresponding TEM images (middle panel) and size distribution histograms (right panel).

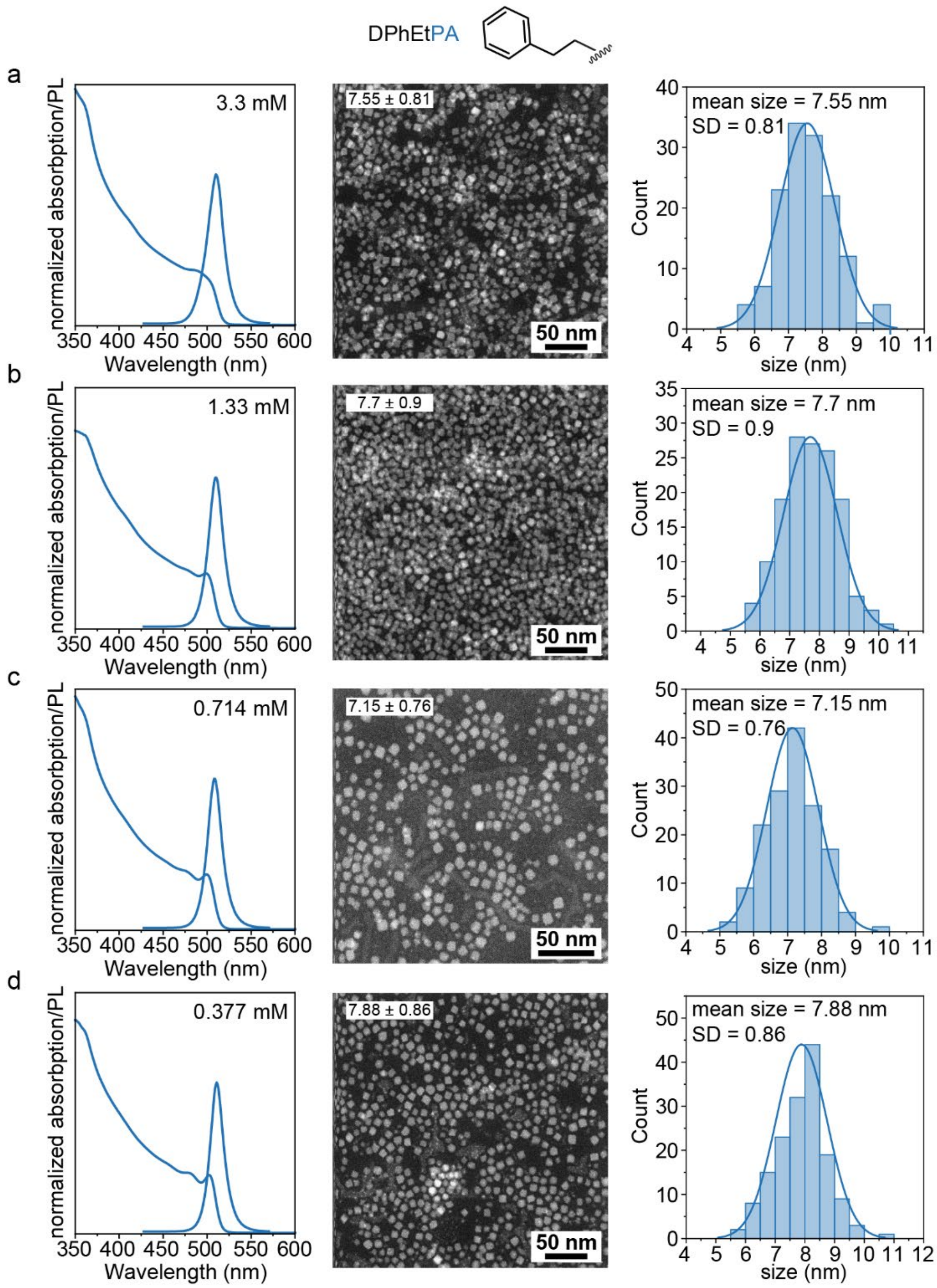

Figure S14. The representative normalized absorption and photoluminescence (PL) spectra (left panel) of $CsPbBr_3$ QDs synthesized using Cs diphenylethylphosphinic acid (Cs-DPhEtPA) at different dilutions: **(a)** 3.33 mM, **(b)** 1.33 mM, **(c)** 0.714 mM, **(d)** 0.377 mM. Corresponding TEM images and size distribution histograms are shown in the middle and right panels.

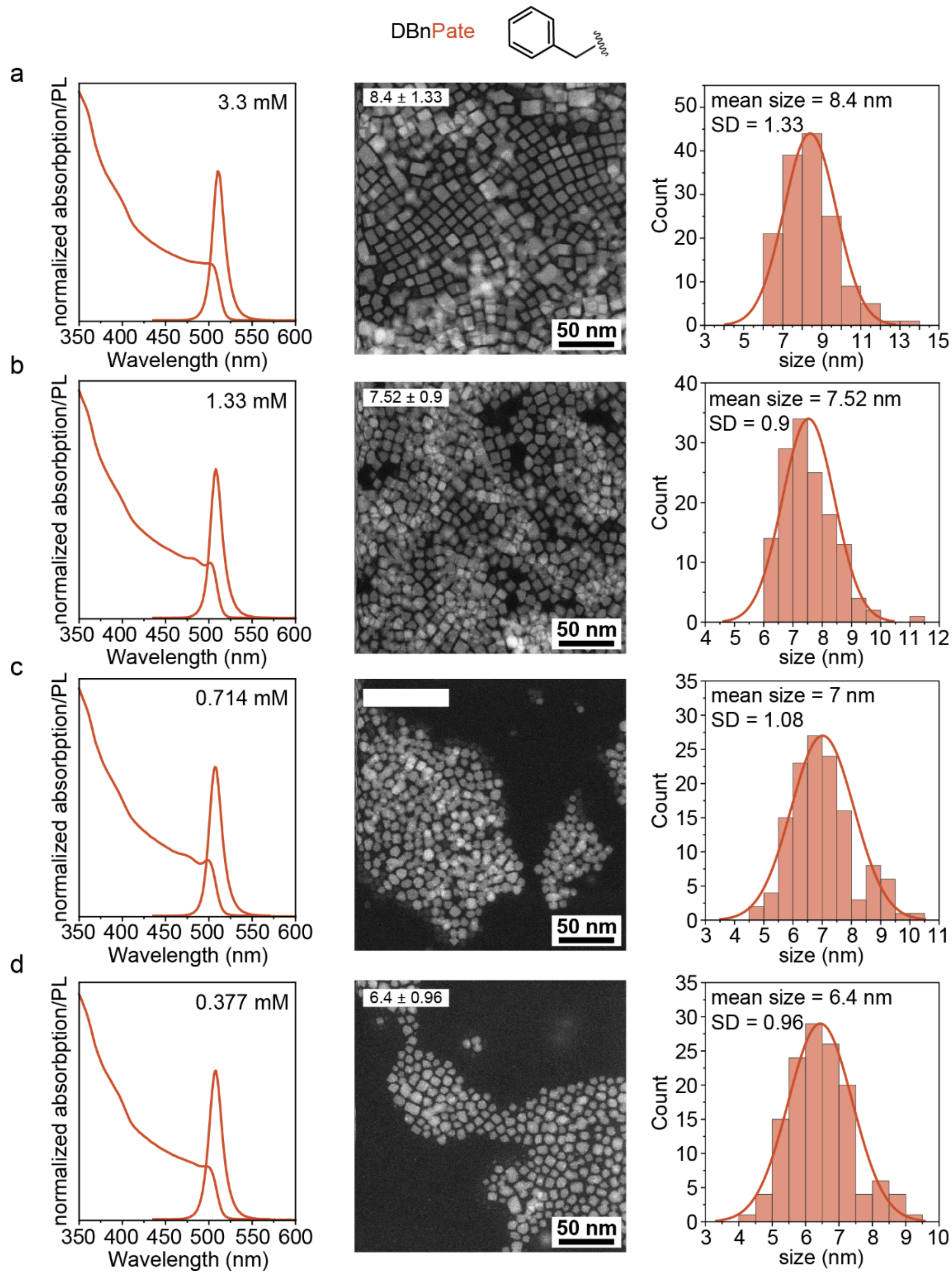

Figure S15. The representative normalized absorption and photoluminescence (PL) spectra (left panel) of $CsPbBr_3$ QDs synthesized using Cs dibenzylphosphate (Cs-DBnPate) at different dilutions **(a)** 3.33 mM, **(b)** 1.33 mM, **(c)** 0.714 mM, **(d)** 0.377 mM. Corresponding TEM images and size distribution histograms are shown in the middle and right panels.

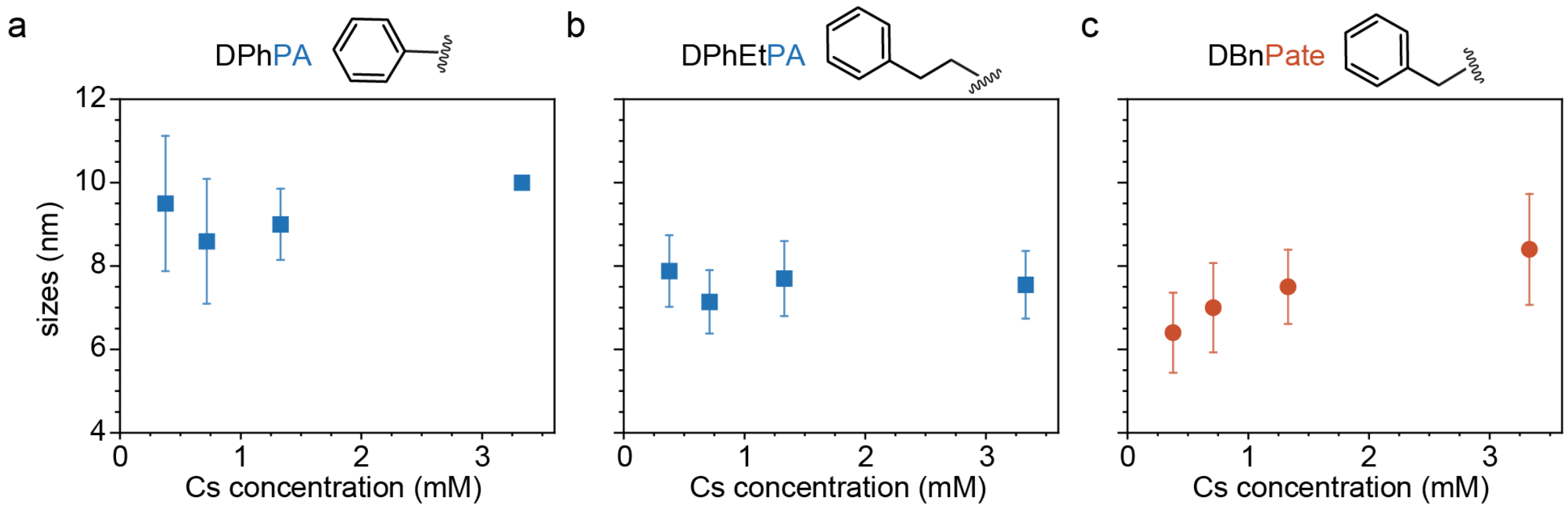

Figure S16. The size charts of $CsPbBr_3$ QDs synthesized at different dilutions using different aromatic phosphinates and phosphates: **(a)** Cs diphenylphosphinic acid (Cs-DPhPA), **(b)** Cs diphenylethylphosphinic acid (Cs-DPhEtPA), **(c)** Cs dibenzylphosphate (Cs-DBnPate).

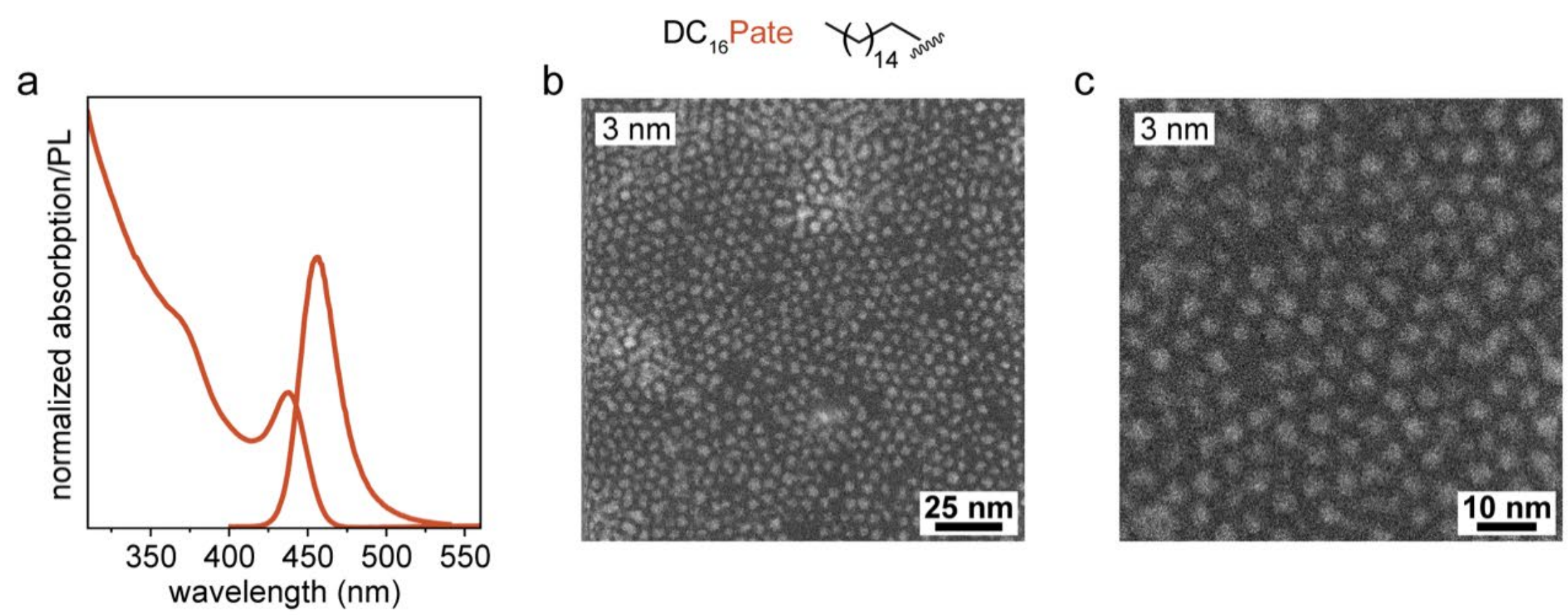

Figure S17. **(a)** The representative normalized absorption and photoluminescence (PL) spectra of 3 nm $CsPbBr_3$ QDs synthesized using Cs-DC_{12}PA. **(b-c)** Low- and high-resolution dark-field STEM images of 3 nm $CsPbBr_3$ QDs.

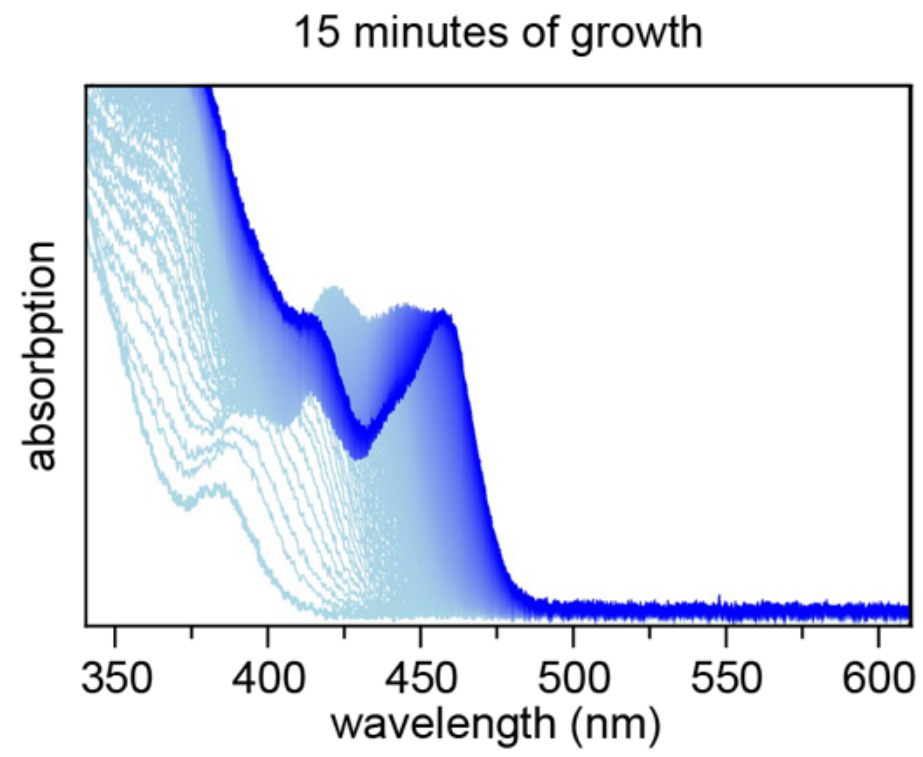

Figure S18. Evolution of the absorption spectra of $CsPbBr_3$ QDs synthesized using a Cs-DC_{16}Pate precursor over the course of the reaction (15 min). Under in situ conditions, smaller QDs are observed, with excitonic peak positions down to ~380 nm (3.26 eV), corresponding to estimated sizes of ~2.0-2.5 nm. This enables direct monitoring of QD formation at very early stages, corresponding to sizes of approximately 3-4 unit cells.

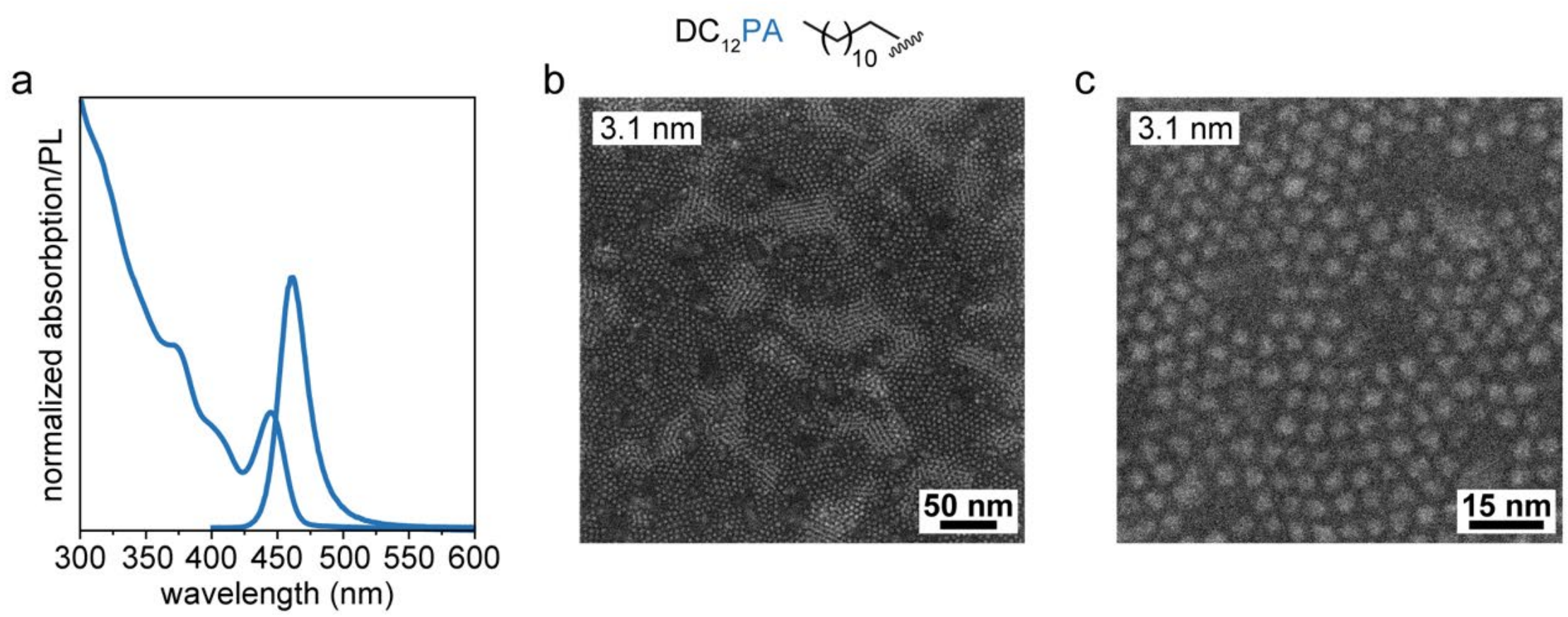

Figure S19. (a) The representative normalized absorption and photoluminescence (PL) spectra of 3.1 nm $CsPbBr_3$ QDs synthesized using Cs-DC_{12}PA. **(b-c)** Low- and high-resolution dark-field STEM images of 3.1 nm $CsPbBr_3$ QDs.

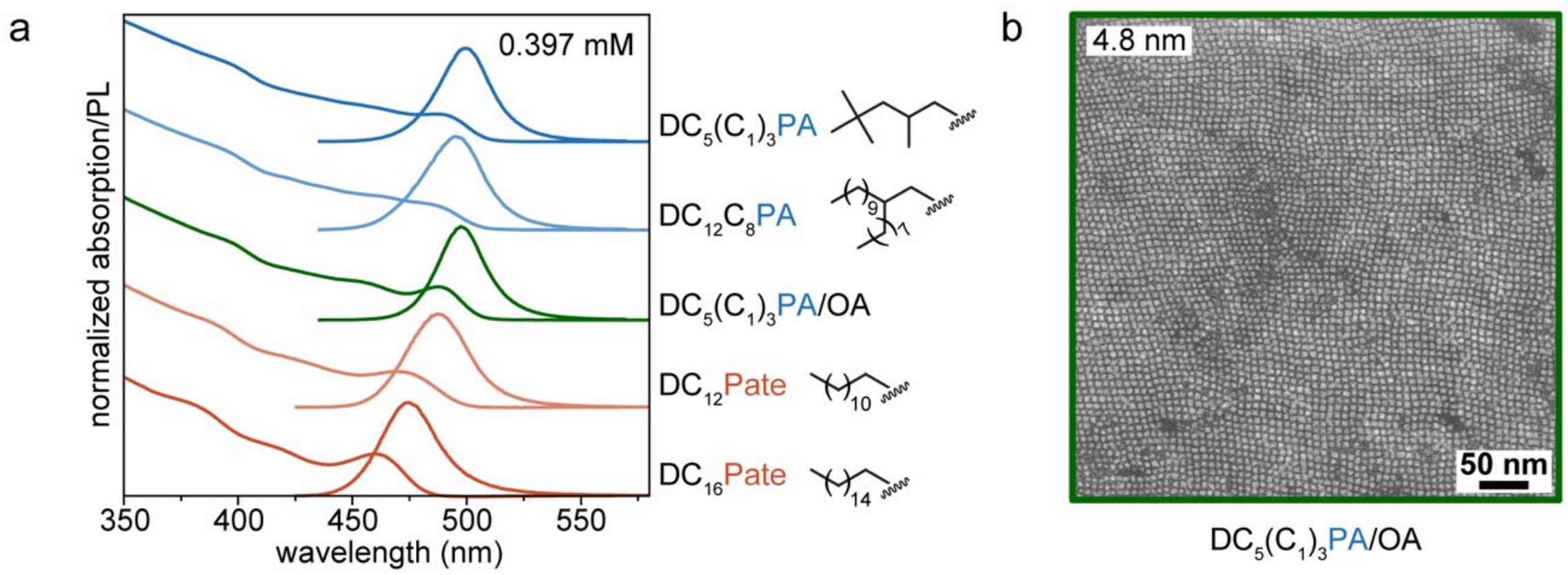

Figure S20. (a) The representative normalized absorption and photoluminescence (PL) spectra of $FAPbBr_3$ QDs synthesized at 0.397 mM dilution using FA-DC_{16}Pate (orange), FA-DC_{12}Pate (pale orange), FA-$DC_5(C_1)_3$PA/OAc used as the closest analogue to the previously reported precursor FA-DOPA/OA (green), FA-$DC_{12}C_8$PA (pale blue), and FA-$DC_5(C_1)_3$PA (blue). **(b)** Dark-field STEM image of $FAPbBr_3$ QDs synthesized at 0.397 mM dilution using FA-DOPA/OA precursor.

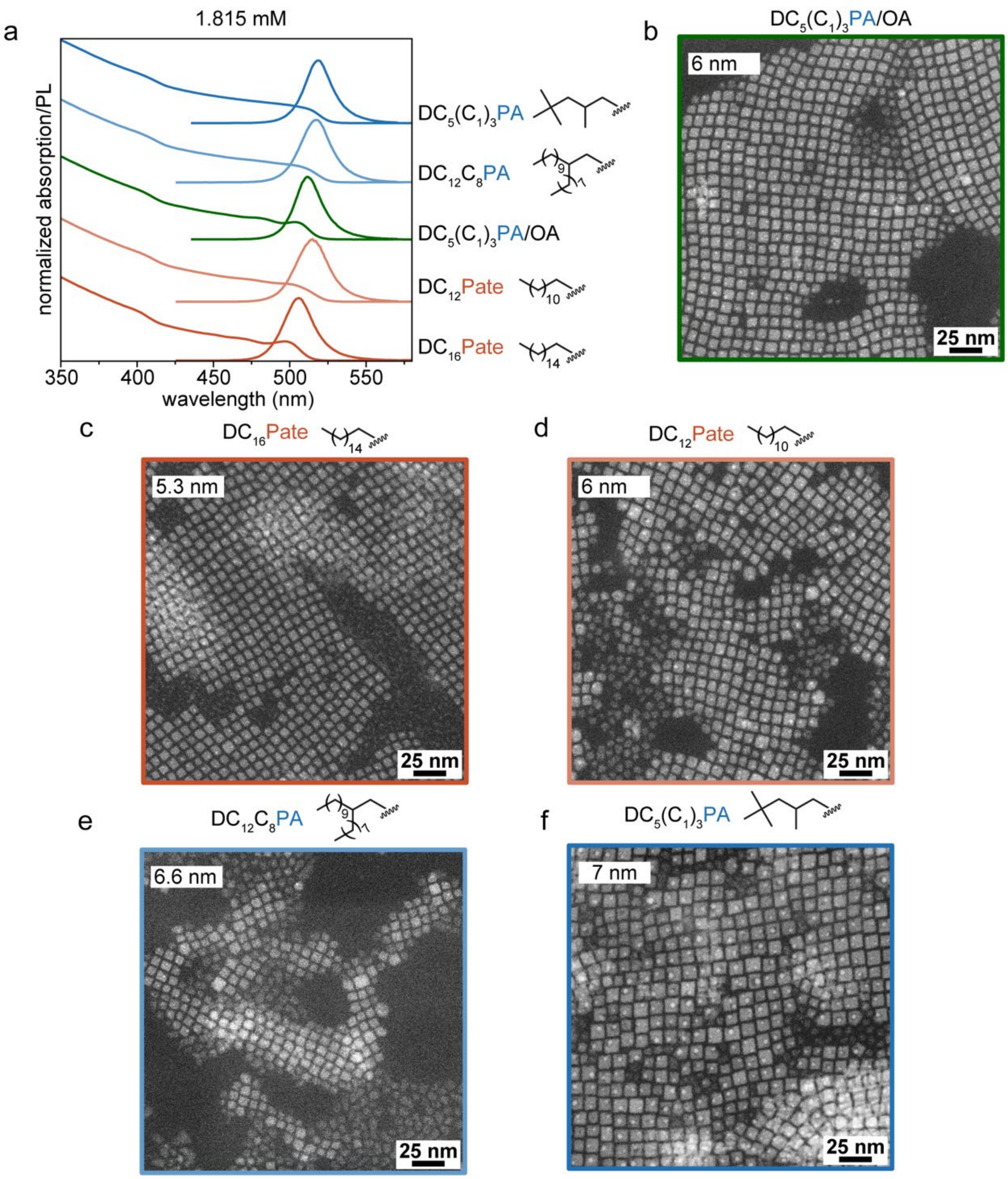

Figure S21. (a) The representative normalized absorption and photoluminescence (PL) spectra of $FAPbBr_3$ QDs synthesized at 1.815 mM dilution using FA-DC_{16}Pate (orange), FA-DC_{12}Pate (pale orange), FA-$DC_5(C_1)_3$PA/OAc used as the closest analogue to the previously reported FA-DOPA/OA (green), FA-$DC_{12}C_8$PA (pale blue) and FA-$DC_5(C_1)_3$PA (blue) precursors. **(b-f)** Dark-field STEM images of $FAPbBr_3$ QDs synthesized at 1.815 mM dilution using FA (**b**; green frame), FA-DC_{16}Pate (**c**; orange frame), FA-DC_{12}Pate (**d**; pale orange frame), FA-$DC_{12}C_8$PA (**e**; pale blue frame) and FA-$DC_5(C_1)_3$PA (**f**; blue frame) precursors.

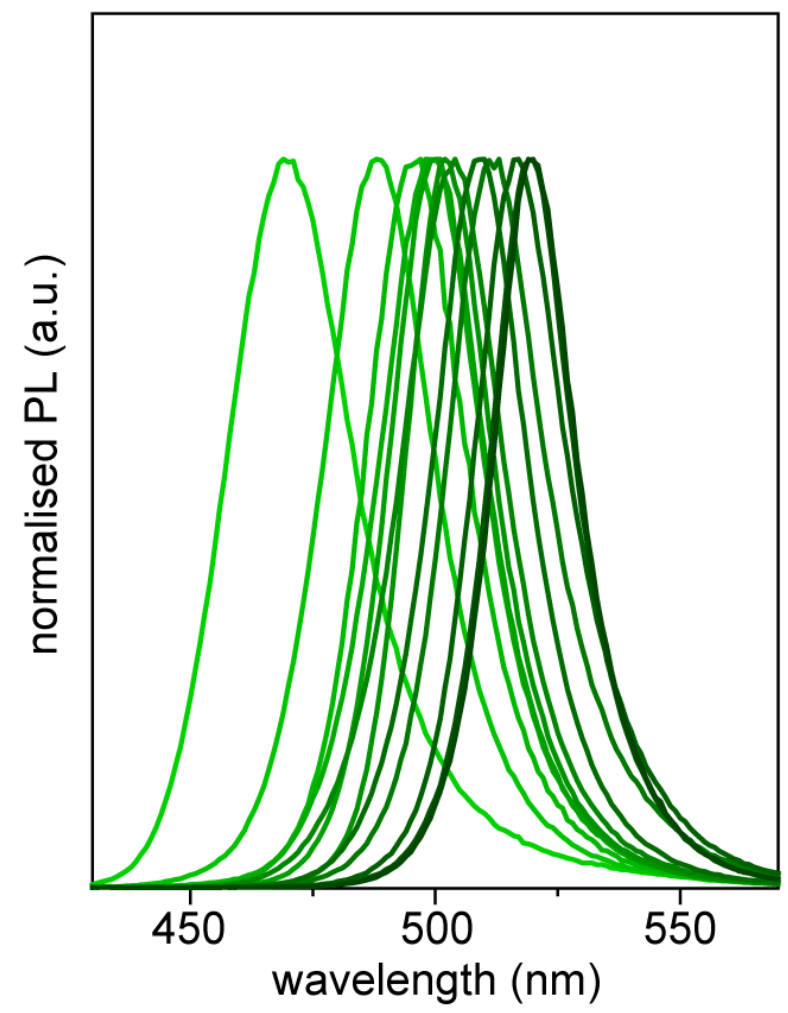

Figure S22. PL spectra of purified $FAPbBr_3$ QDs with sizes ranging from 3.78 to 8.5 nm.

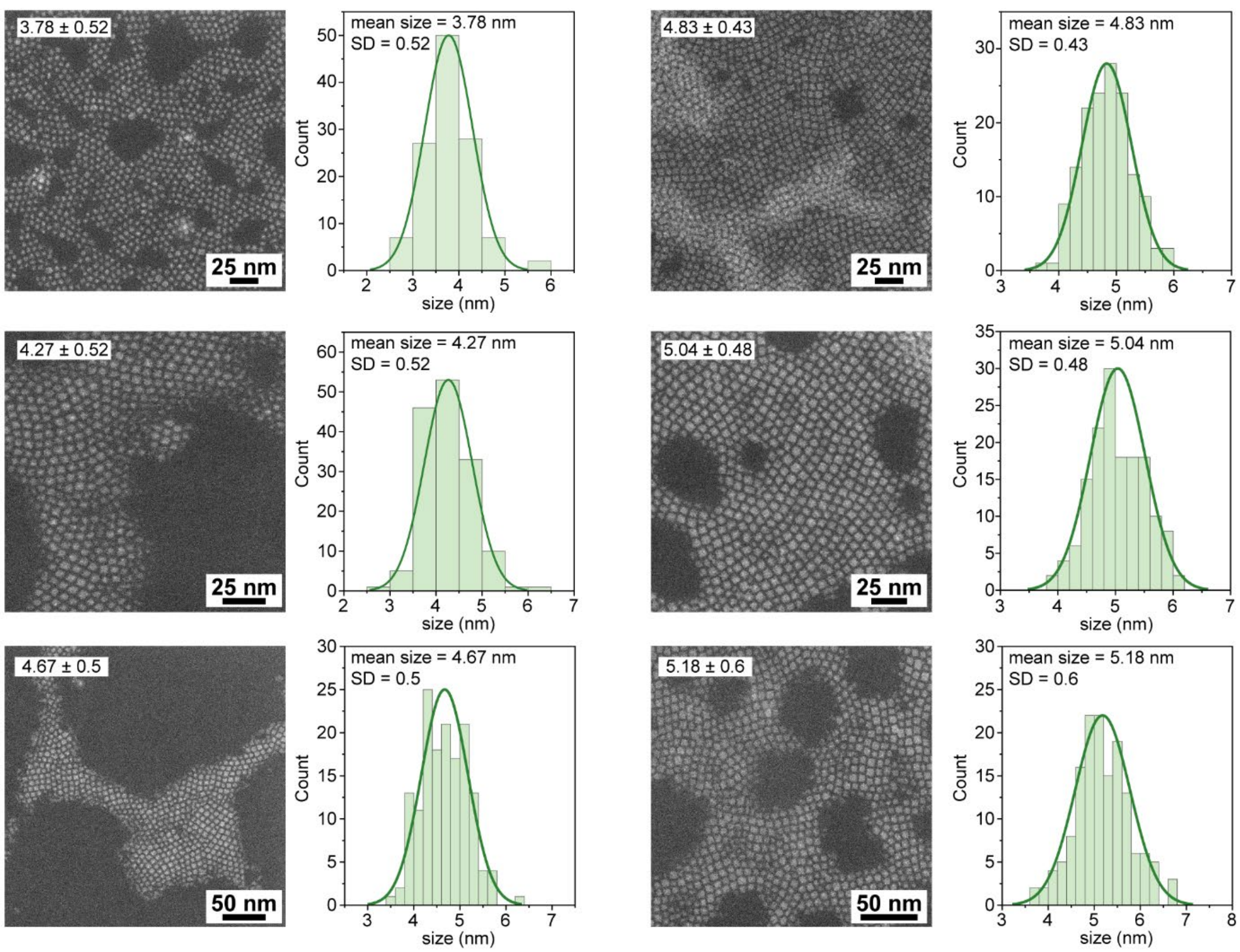

Figure S23. Dark-field STEM images of various sizes of $FAPbBr_3$ QDs ranging from 3.78 to 5.18 nm and corresponding size distribution histograms. The synthetic conditions for each sample are summarized in Table S2.

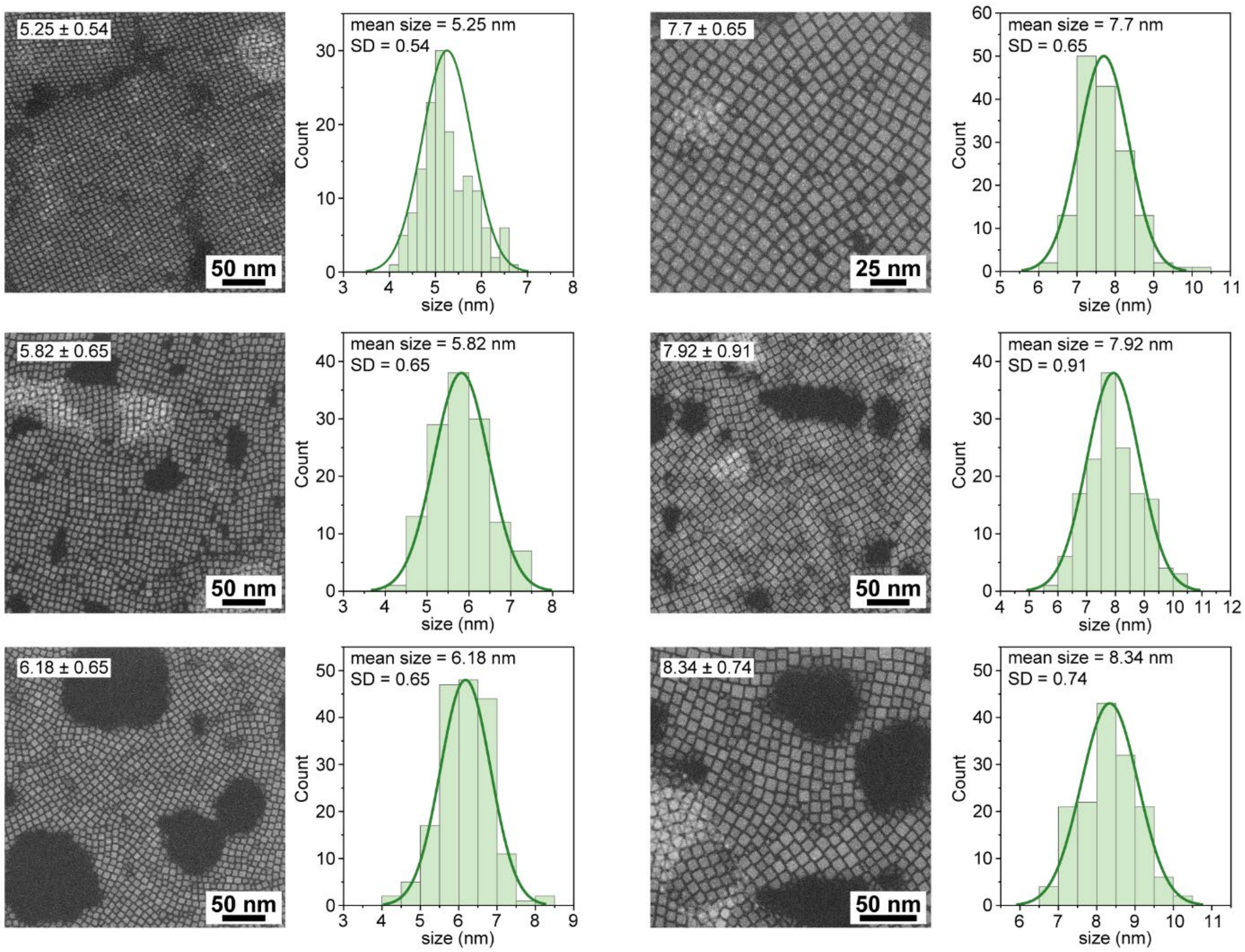

Figure S24. Dark-field STEM images of various sizes of $FAPbBr_3$ QDs from 5.25 to 8.34 nm and corresponding size distribution histograms. The synthetic conditions for each sample are summarized in Table S2.

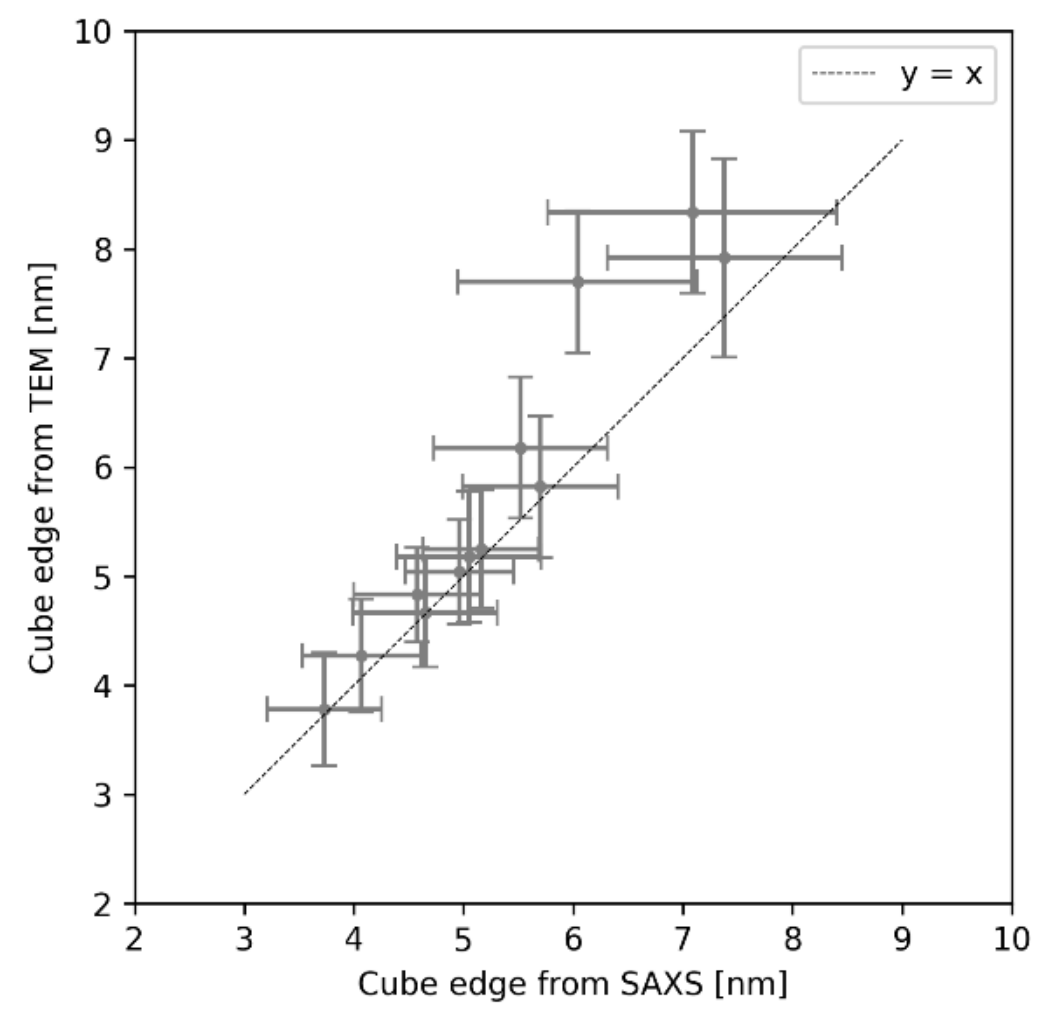

Figure S25. Comparison of sizes determined by TEM and SAXS. The sizes are in good agreement, with a minor overestimation of the sizes by TEM for QDs larger than 6 nm.

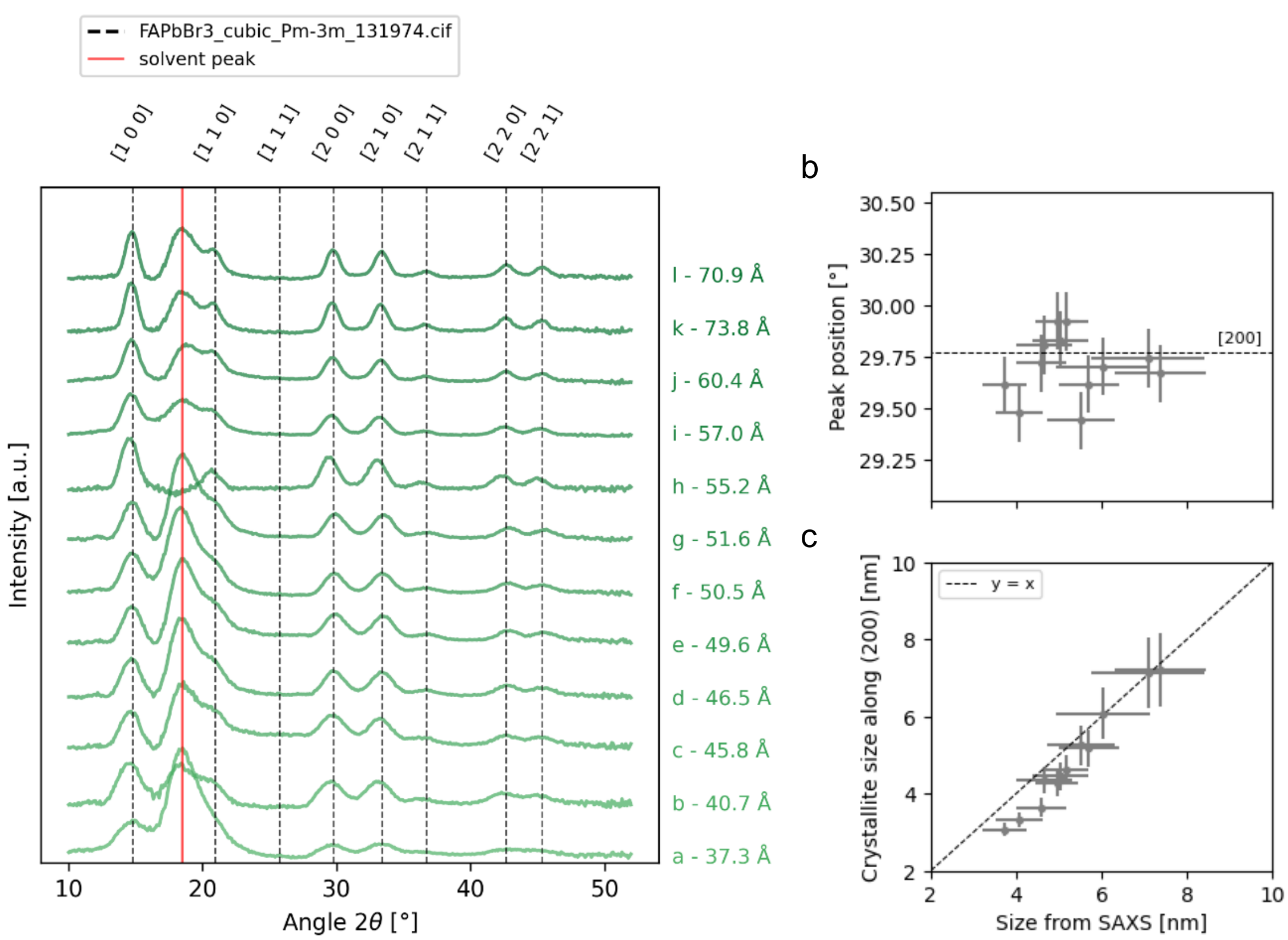

Figure S26. (a) WAXS patterns for the various sizes of cubic $FAPbBr_3$ QDs match the reported cubic structure (ISCD 131974). The peak at 18.4° arises from the residual solvent signal. **(b)** Peak position of the (200) reflection as a function of QD size. No measurable shift indicates the absence of significant lattice expansion

with decreasing size. **(c)** Crystallite size along the (200) direction – equivalent to the cube edge – showing a good agreement with the measured SAXS size.

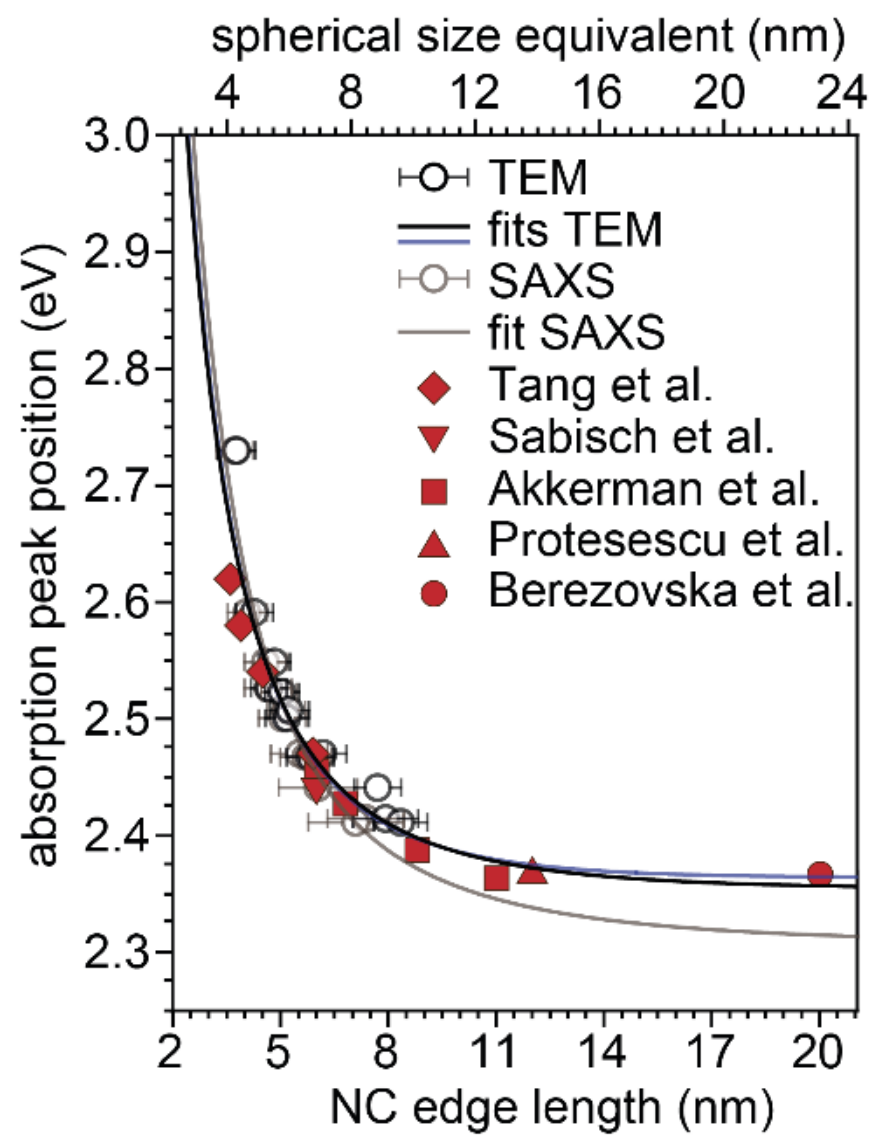

Figure S27. The sizing curve as presented in Figure 6d of the manuscript, but showing additionally the spherical size equivalent calculated as described in Ref.[15] Here, the size-dependent absorption peak position for $FAPbBr_3$ QDs is shown as a function of QD size determined by TEM (black) and SAXS (pale brown). Solid lines represent the fit to the semi-empirical expression from Ref.[15] using both experimental data (open circles, with error bars indicating standard deviations) and literature data (orange filled markers)[7, 8, 19-21] for the TEM dataset and experimental data (open circles, with error bars indicating standard deviations) for the SAXS dataset. Solid black and blue lines represent fits obtained with dielectric constant measured by magneto-optical technique (8.6)[16] and ellipsometry (6.0).[17]

6. Supporting tables

Table S1. Reaction conditions for all $CsPbBr_3$ QD samples reported in the main text and supporting information.

Figures	acid used	dilution of the synthesis expressed as [Cs] (mM)	solvent	time of reaction (min)	final size of QDs (nm)	Final number of QDs* (10^{15})	Yield of reaction*
Fig.1a, 2c, 4a	Cs-DC_{12}PA	3.33	hexane	10	6.52	1.30	7.72%
Fig. 1a, 2c	Cs-DC_{12}PA	1.33	hexane	15	5.31	1.94	6.19%
Fig. 1a, 2c	Cs-DC_{12}PA	0.714	hexane	30	4.63	3.26	6.92%**
Fig. 1a, 2c, 3, 4a	Cs-DC_{12}PA	0.377	hexane	60	4.08	5.35	7.73%
Fig. 1a, 4a	Cs-DC_{12}Pate	3.33	hexane	10	7.45	9.74	85.95%
Fig. 1a	Cs-DC_{12}Pate	1.33	hexane	15	5.84	22.21	94.41%
Fig. 1a	Cs-DC_{12}Pate	0.714	hexane	30	5.31	28.39	90.73%
Fig. 1a, 4a	Cs-DC_{12}Pate	0.377	hexane	60	4.67	34.47	74.88%
Fig. 1b, 4a, 5a	Cs-DC_6C_2PA	3.33	hexane	10	10.54	2.94	73.32%
Fig. 1b, 5a-c	Cs-DC_6C_2PA	1.33	hexane	15	6.85	8.76	60.09%
Fig. 1b, 5a	Cs-DC_6C_2PA	0.714	hexane	30	6.09	14.93	71.98%
Fig. 1b, 4a, 5a	Cs-DC_6C_2PA	0.377	hexane	60	5.13	20.50	59.06%
Fig. 1b, 4a	Cs-DC_6C_2Pate	3.33	hexane	10	8.20	5.54	65.21%
Fig. 1b	Cs-DC_6C_2Pate	1.33	hexane	15	6.37	13.74	75.74%
Fig. 1b	Cs-DC_6C_2Pate	0.714	hexane	30	5.72	16.88	67.43%
Fig. 1b, 4a	Cs-DC_6C_2Pate	0.377	hexane	60	5.22	26.13	79.29%
Fig. 1c, 4	Cs-DC_4PA	3.33	hexane	10	17.80	1.92	67.12%
Fig. 1c, 4b	Cs-DC_4PA	1.33	hexane	15	9.80	3.95	79.37%
Fig. 1c, 4b	Cs-DC_4PA	0.714	hexane	30	7.04	7.90	58.75%
Fig. 1c, 4	Cs-DC_4PA	0.377	hexane	60	5.31	21.29	67.98%
Fig. 1c, 4a	Cs-DC_4Pate	3.33	hexane	10	10.9 ± 1.85	2.73	75.51%
Fig. 1c	Cs-DC_4Pate	1.33	hexane	15	8.82	6.04	88.32%
Fig. 1c	Cs-DC_4Pate	0.714	hexane	30	7.24	8.44	68.37%
Fig. 1c, 4a	Cs-DC_4Pate	0.377	hexane	60	9.58	4.63	86.82%
Fig. 2a, 4	Cs-DC_8PA	3.33	hexane	10	14.4	1.43	91.40%

Figures	acid used	dilution of the synthesis expressed as [Cs] (mM)	solvent	time of reaction (min)	final size of QDs (nm)	Final number of QDs* (10^{15})	Yield of reaction*
Fig. 2a, 4	Cs-DC_8PA	1.33	hexane	15	10.54	2.89	72.18%
Fig. 2a, 4	Cs-DC_8PA	0.714	hexane	30	9.58	5.07	95.14%
Fig. 2a, 3, 4	Cs-DC_8PA	0.377	hexane	60	7.34	13.13	99.67%
Fig. 2b, 4	Cs-$DC_{10}PA$	3.33	hexane	10	9.58	4.59	86.23%
Fig. 2b, 4a	Cs-$DC_{10}PA$	1.33	hexane	15	7.68	8.31	80.36%
Fig. 2b, 4a	Cs-$DC_{10}PA$	0.714	hexane	30	6.22	17.27	88.73%
Fig. 2b, 3, 4	Cs-$DC_{10}PA$	0.377	hexane	60	4.96	35.49	92.42%
Fig. 4, 5a	Cs-DC_6PA	3.33	hexane	10	10.03	3.82	82.36%
Fig. 4b, 5a-c	Cs-DC_6PA	1.33	hexane	15	7.04	11.81	87.90%
Fig. 4b, 5a	Cs-DC_6PA	0.714	hexane	30	6.09	19.55	94.25%
Fig. 4, 5a	Cs-DC_6PA	0.377	hexane	60	4.96	38.01	98.94%
Fig. 4a, S5a, S6	Cs-$DC_{16}Pate$	3.33	hexane	10	4.18	41.93	65.30%
Fig. S5b, S6	Cs-$DC_{16}Pate$	1.33	hexane	15	4.21	51.05	81.31%
Fig. S5c, S6	Cs-$DC_{16}Pate$	0.714	hexane	30	4.08	48.95	70.90%
Fig. 4a, S5d, S6	Cs-$DC_{16}Pate$	0.377	hexane	60	4.11	54.06	80.05%
Fig. 5a	Cs-$DC_5(C_1)_3PA$	3.33	hexane	10	10.54	3.85	96.24%
Fig. 5a-c	Cs-$DC_5(C_1)_3PA$	1.33	hexane	15	7.04	10.55	78.56%
Fig. 5a	Cs-$DC_5(C_1)_3PA$	0.714	hexane	30	6.22	17.06	87.61%
Fig. 5a	Cs-$DC_5(C_1)_3PA$	0.377	hexane	60	5.45	26.65	92.06%
Fig. 5d	Cs-$DC_{12}C_8PA$	3.33	hexane	10	8.82	5.95	87.1%
Fig. 5d	Cs-$DC_{12}C_8PA$	1.33	hexane	15	6.37	10.16	56.1%
Fig. 5d	Cs-$DC_{12}C_8PA$	0.714	hexane	30	5.72	17.79	71%
Fig. 5d-f	Cs-$DC_{12}C_8PA$	0.377	hexane	60	5.13	24.75	71.3%
Fig. S1a, S2a	Cs-C_6PA	3.33	hexane	10	8.49	5.12	66.92%
Fig. S1b, S2a	Cs-C_6PA	1.33	hexane	15	6.37	13.69	75.52%
Fig. S1c, S2a	Cs-C_6PA	0.714	hexane	30	6.52	13.12	77.6%
Fig. S1d, S2a	Cs-C_6PA	0.377	hexane	60	5.3	23.15	73.5%

Figures	acid used	dilution of the synthesis expressed as [Cs] (mM)	solvent	time of reaction (min)	final size of QDs (nm)	Final number of QDs* (10^{15})	Yield of reaction*
Fig. S1e, S2b	Cs-C_{12}PA	3.33	hexane	10	6.5	12.15	71.2%
Fig. S1f, S2b	Cs-C_{12}PA	1.33	hexane	15	5.3	22.41	71.2%
Fig. S1g, S2b	Cs-C_{12}PA	0.714	hexane	30	4.7	33.85	75%
Fig. S1h, S2b	Cs-C_{12}PA	0.377	hexane	60	4.4	41.02	74.6%
Fig. S3a, S4a	Cs-C_8POA	3.33	hexane	10	5.13	26.27	75.65%
Fig. S3b, S4a	Cs-C_8POA	1.33	hexane	15	4.53	26.12	51.79%
Fig. S3c, S4a	Cs-C_8POA	0.714	hexane	30	4.41	24.63	45.12%
Fig. S3d, S4a	Cs-C_8POA	0.377	hexane	60	4.18	39.38	61.38%
Fig. S3e, S4b	Cs-C_{12}POA	3.33	hexane	10	7.14	12.32	95.71%
Fig. S3f, S4b	Cs-C_{12}POA	1.33	hexane	15	5.61	25.40	95.71%
Fig. S3g, S4b	Cs-C_{12}POA	0.714	hexane	30	5.31	21.40	68.37%
Fig. S3h, S4b	Cs-C_{12}POA	0.377	hexane	60	5.22	20.99	63.68%
Fig. S10a, S11-12	Cs-DOlPA	3.33	hexane	10	12.7 ± 1.12	1.74	75.9%
Fig. S10b, S12	Cs-DOlPA	1.33	hexane	15	10	4.32	92.3%
Fig. S10c, S12	Cs-DOlPA	0.714	hexane	30	8.3	7.62	92.9%
Fig. S10d, S12	Cs-DOlPA	0.377	hexane	60	7	11.32	82.8%
Fig. S13a, S16a	Cs-DPhPA	3.33	toluene	10	10	3.13	66.7%
Fig. S13b, S16a	Cs-DPhPA	1.33	toluene	15	9 ± 0.85	5.57	86.6%
Fig. S13c, S16a	Cs-DPhPA	0.714	toluene	30	8.6 ± 1.5	5.64	76.6%
Fig. S13d, S16a	Cs-DPhPA	0.377	toluene	60	9.47 ± 1.62	4.73	85.7%
Fig. S14a, S16b	Cs-DPhEtPA	3.33	toluene	10	7.55 ± 0.81	8.55	78.6%
Fig. S14b, S16b	Cs-DPhEtPA	1.33	toluene	15	7.7 ± 0.9	7.25	70.6%
Fig. S14c, S16b	Cs-DPhEtPA	0.714	toluene	30	7.15 ± 0.76	5.13	40%
Fig. S14d, S16b	Cs-DPhEtPA	0.377	toluene	60	7.88 ± 0.86	6.09	63.6%
Fig. S15a, S16c	Cs-DBnPate	3.33	hexane	10	8.4 ± 1.33	5.34	67.6%
Fig. S15b, S16c	Cs-DBnPate	1.33	hexane	15	7.52 ± 0.9	6.84	62.5%
Fig. S15c, S16c	Cs-DBnPate	0.714	hexane	30	7 ± 1.08	7.03	51.5%

Figures	acid used	dilution of the synthesis expressed as [Cs] (mM)	solvent	time of reaction (min)	final size of QDs (nm)	Final number of QDs* (10^{15})	Yield of reaction*
Fig. S15d, S16c	Cs-DBnPate	0.377	hexane	60	6.4 ± 0.96	7.20	40.3%
Fig.S17	Cs-DC_{16}Pate	1.33	hexane	10 seconds	3	61.2	86.67%
Fig.S18	Cs-DC_{12}PA	0.15	hexane	5	3.1	46.92***	23.85%

* The final numbers of QDs and yields of the reactions were calculated after isolation of QDs.

** This sample was synthesized at half the scale without changing concentrations. When synthesized at the regular scale (corresponding to 2 mL Pb precursor), the final QD size is the same (4.63 nm) and the reaction yield is 9.12%.

*** This sample was synthesized at 2.5x the scale corresponding to 5 mL Pb precursor.

Table S2. Reaction conditions for $FAPbBr_3$ QDs reported in the main text and supporting information.

Figures	FA precursor used	Dilution of the synthesis (mM of FA)	Hexane (mL)	$PbBr_2$-TOPO stock solution (µL)	FA precursor solution (µL)	Growth time (s)	C8C12-PEA solution (µL)	Absorption peak position (nm)	Absorption peak position (eV)	Final size of QDs (nm)
Fig.6b-e, S20a, S22, S23, S25, S26	FA-DC_{16}Pate	0.397	15	260	100	10	50	454	2.731	3.78 ± 0.52
Fig.6b, S20a	FA-DC_{12}Pate	0.397	15	260	100	10	50	475	2.610	4.5
Fig.6b, S20a	FA-$DC_{12}C_8$PA	0.397	15	260	100	10	50	487	2.546	4.7
Fig.6b, S20a	FA-$DC_5(C_1)_3$PA	0.397	15	260	100	10	50	492	2.520	5
Fig.6c-e, S22, S23, S25, S26	FA-$DC_5(C_1)_3$PA /OAc	0.128	75	1000	160	10	100	478	2.594	4.27 ± 0.52
Fig.6c-e, S22, S23, S25, S26	FA-$DC_5(C_1)_3$PA /OAc	0.604	15	1000	160	10	100	491	2.525	4.67 ± 0.5
Fig.6c-e, S22, S23, S25, S26	FA-$DC_5(C_1)_3$PA /OAc	0.191	50	1000	160	10	100	486	2.551	4.83 ± 0.43
Fig.6c-e, S22, S23, S25, S26	FA-$DC_5(C_1)_3$PA /OAc	0.461	20	1000	160	10	100	492	2.520	5.04 ± 0.48
Fig.6c-e, S22, S23, S25, S26	FA-$DC_5(C_1)_3$PA /OAc	0.875	10	1000	160	10	100	496	2.500	5.18 ± 0.6
Fig.6c-e, S22, S24-26	FA-$DC_5(C_1)_3$PA /OAc	0.313	30	1000	160	10	100	494	2.510	5.25 ± 0.54
Fig.6c-e, S22, S24-26	FA-$DC_5(C_1)_3$PA /OAc	1.363	6	1000	160	10	100	503	2.465	5.82 ± 0.65
Fig.6c-e, S22, S24-26	FA-$DC_5(C_1)_3$PA /OAc	1.363	6	1000	160	10	100	502	2.470	6.18 ± 0.65
Fig.6b-d, S22, S24-26	FA-$DC_5(C_1)_3$PA /OAc	2.346	3	1000	160	10	100	508	2.441	7.7 ± 0.65
Fig.6c-e, S22, S24-26	FA-$DC_5(C_1)_3$PA /OAc	4.519	1	1000	160	10	100	514	2.412	7.92 ± 0.91
Fig.6c-e, S22, S24-26	FA-$DC_5(C_1)_3$PA /OAc	2.346	3	1000	160	20	100	514.3	2.411	8.34 ± 0.74
Fig. S20	FA-$DC_5(C_1)_3$PA /OAc	0.397	15	260	100	10	50	489	2.535	4.8

Figures	FA precursor used	Dilution of the synthesis (mM of FA)	Hexane (mL)	$PbBr_2$-TOPO stock solution (µL)	FA precursor solution (µL)	Growth time (s)	C8C12-PEA solution (µL)	Absorption peak position (nm)	Absorption peak position (eV)	Final size of QDs (nm)
Fig. S21 a,c	FA-DC_{16}Pate	1.815	3	260	100	10	50	499	2.485	5.3
Fig. S21 a,d	FA-DC_{12}Pate	1.815	3	260	100	10	50	505	2.455	6
Fig. S21 a,d	FA standard	1.815	3	260	100	10	50	505	2.455	6
Fig. S21 a,e	FA-$DC_{12}C_8$PA	1.815	3	260	100	10	50	510	2.431	6.8
Fig. S21 a,f	FA-$DC_5(C_1)_3$PA	1.815	3	260	100	10	50	512	2.421	7

Table S3: Fit results of the SAXS for the cubic model with a size polydispersity.

**Sample**	**a**	**b**	**c**	**d**	**e**	**f**	**g**	**h**	**i**	**j**	**k**	**l**
Vol. Frac. (10^{-3})	2.03	2.38	1.85	2.80	2.30	2.97	2.70	2.80	3.44	2.62	1.98	2.50
Edge length, Å	37.3	40.7	45.8	46.5	49.6	50.3	51.6	54.7	56.7	60.6	73.8	70.86
Polydispersity	0.140	0.133	0.127	0.141	0.100	0.140	0.102	0.158	0.132	0.176	0.145	0.186
χ^2	5.72	4.63	5.13	9.51	8.7	9.2	11.22	7.69	10.2	30	5.78	36

Table S4. Bandgap E_{bulk} and Bohr radius a_B fitted from the dependence of the excitonic absorption peak position on the sizes extracted from SAXS and TEM. Parameter α was fixed at 0.7.[15] Dielectric constant was taken either from ref.[16] (8.6) or from ref.[17] (6.0) with similar fitting results. Refined curves are shown in the main text and in Figure S27. Reduced effective mass μ was calculated from the fitted Bohr diameter to be compared with the literature data. Fitted bandgap, Bohr diameter, and reduced effective mass are in good agreement with literature: 2.30 eV,[17] 7.0-7.8 nm,[16,18] and 0.13m_0 respectively.[16]

	E_{bulk}, eV	Bohr diameter, nm	μ, m_0
SAXS	2.29±0.02	7.5±0.7	0.12
TEM (ε=8.6)	2.35±0.01	5.6±0.4	0.16
TEM (ε=6.0)	2.36±0.01	4.0±0.3	0.16

7. Supporting information references

1. E. Dhaene, R. Pokratath, O. Aalling-Frederiksen, K. M. Ø. Jensen, P. F. Smet, K. De Buysser and J. De Roo, ACS Nano, 2022, 16, 7361–7372.
2. Author*, China Pat.,* CN104004015A, 2014.
3. B. Verbelen, W. Dehaen and K. Binnemans, Synthesis, 2018, 50, 2019–2026.
4. J. De Roo, Z. Zhou, J. Wang, L. Deblock, A. J. Crosby, J. S. Owen and S. S. Nonnenmann, Chem. Mater., 2018, 30, 8034–8039.
5. R. A. Aitken, C. J. Collett and S. T. E. Mesher, Synthesis, 2012, 44, 2515–2518.
6. V. Morad, A. Stelmakh, M. Svyrydenko, L. G. Feld, S. C. Boehme, M. Aebli, J. Affolter, C. J. Kaul, N. J. Schrenker, S. Bals, Y. Sahin, D. N. Dirin, I. Cherniukh, G. Raino, A. Baumketner and M. V. Kovalenko, Nature, 2024, 626, 542–548.
7. Y. Berezovska, S. Sabisch, C. Bernasconi, Y. Sahin, F. Bertolotti, A. Guagliardi, M. I. Bodnarchuk, D. N. Dirin and M. V. Kovalenko, J. Am. Chem. Soc., 2025, 147, 35446–35455.
8. Q. A. Akkerman, T. P. T. Nguyen, S. C. Boehme, F. Montanarella, D. N. Dirin, P. Wechsler, F. Beiglböck, G. Rainò, R. Erni, C. Katan, J. Even and M. V. Kovalenko, Science, 2022, 377, 1406–1412.
9. J. Maes, L. Balcaen, E. Drijvers, Q. Zhao, J. De Roo, A. Vantomme, F. Vanhaecke, P. Geiregat and Z. Hens, J. Phys. Chem. Lett., 2018, 9, 3093–3097.
10. F. Montanarella, Q. A. Akkerman, D. Bonatz, M. M. van der Sluijs, J. C. van der Bok, P. T. Prins, M. Aebli, A. Mews, D. Vanmaekelbergh and M. V. Kovalenko, Nano Lett., 2023, 23, 667–676.
11. A. Jerschow and N. Müller, J. Magn. Reson., 1997, 125, 372–375.
12. A. Jerschow and N. Müller, J. Magn. Reson., Ser. A, 1996, 123, 222–225.
13. E. O. Stejskal and J. E. Tanner, J. Chem. Phys., 1965, 42, 288–292.
14. J. Maes, N. Castro, K. De Nolf, W. Walravens, B. Abécassis and Z. Hens, Chem. Mater., 2018, 30, 3952–3962.
15. T. Aubert, A. A. Golovatenko, M. Samoli, L. Lermusiaux, T. Zinn, B. Abécassis, A. V. Rodina and Z. Hens, Nano Lett., 2022, 22, 1778–1785.
16. K. Galkowski, A. Mitioglu, A. Miyata, P. Plochocka, O. Portugall, G. E. Eperon, J. T.-W. Wang, T. Stergiopoulos, S. D. Stranks, H. J. Snaith and R. J. Nicholas, Energy Environ. Sci., 2016, 9, 962–970.
17. G. Mannino, I. Deretzis, E. Smecca, A. La Magna, A. Alberti, D. Ceratti and D. Cahen, J. Phys. Chem. Lett., 2020, 11, 2490–2496.
18. G. Ammirati, D. Catone, P. O'Keeffe, F. Toschi, S. Turchini, F. Matteocci, J. Barichello, A. Di Carlo and F. Martelli, Phys. Rev. B, 2023, 108, 195201.
19. X. Tang, M. Khurana, D. Rossi, L. Luo, A. V. Akimov and D. H. Son, J. Phys. Chem. C, 2022, 126, 18366–18373.
20. S. Sabisch, O. F. Dressler, O. Ortikova, M. Svyrydenko, S. Elveren, L. G. Feld, L. Dubenska and M. V. Kovalenko, ACS Nano, 2025, 19, 41100–41109.
21. L. Protesescu, S. Yakunin, M. I. Bodnarchuk, F. Bertolotti, N. Masciocchi, A. Guagliardi and M. V. Kovalenko, J. Am. Chem. Soc., 2016, 138, 14202–14205.
